# Supplementary material for: Pilot cluster randomised trial of an evidence-based intervention to reduce avoidable hospital admissions in nursing home residents (Better Health in Residents of Care Homes with Nursing—BHiRCH-NH Study)
Source: BMJ Open. 2020 Dec 13;10(12):e040732. doi: 10.1136/bmjopen-2020-040732 (PMC7737107; doi:10.1136/bmjopen-2020-040732)
Supplement: Supplementary data [file bmjopen-2020-040732supp001.pdf]

Better Health in Residents of Care Homes with Nursing - BHiRCH-NH study

Appendix 1: Pre-intervention characteristics of participating nursing homes

Characteristic	All care homes	TAU	BHiRCH-NH
	n/N or median (% or IQR)		
**Residents and beds**			
Beds available to new residents	1 (1,3)	2 (0, 2)	1 (1, 3)
Resident places	60 (36, 71)	67 (47, 84)	49 (28, 64)
Number of residents present in home	50 (34, 68)	50 (47, 83)	46 (25, 63)
Number of residents with dementia	20 (15, 33)	33 (15, 44)	20 (13, 23)
Number of residents in hospital	0 (0, 1)	1 (0, 2)	0 (0, 1)
**Medical attendances**			
Number hospital admissions	3 (2, 5)	4 (2, 7)	3 (2, 4)
Number of ambulances called	3 (2, 6)	4 (2, 9)	3 (2, 6)
Unscheduled (out of hours) GP visits or telephone contacts	1 (1, 3)	2 (1, 3)	1 (1, 3)
Accident and Emergency attendances	3 (2, 5)	3 (1, 4)	3 (2, 6)
**Staffing**			
Qualified nursing staff rostered on during the day	3 (2, 5)	3 (2, 5)	3 (2, 5)
Care staff rostered on during the day	11 (9, 13)	11 (9, 14)	12 (6, 13)
Qualified nursing staff rostered on during the night	2 (1, 3)	2 (1, 3)	2 (1, 3)
Care staff rostered on during the night	3 (3, 7)	3 (3, 7)	4 (2, 7)
Number of agency/ bank staff in 24 hour period	1 (0, 3)	1 (0, 3)	1 (0, 3)
Number of permanent registered nursing staff (including those on sick/carer/compassionate leave)	10 (8, 13)	11 (10, 13)	8 (7, 15)
Number of permanent other care staff (including those on sick/carer/compassionate leave)	26 (24, 57)	26 (24, 70)	33 (23, 57)
Number of registered nursing staff from those above on sick/carer/ compassionate leave	0 (0, 1)	0 (0, 1)	0 (0, 1)
Number of other care staff from those above on sick/carer/ compassionate leave	0 (0, 2)	1 (0, 6)	0 (0, 2)
**Nursing home**			
Privately managed	12/13 (92)	6/7 (86)	6/6 (100)
Local Authority managed	1/13 (8)	1/7 (14)	0/6 (0)
Nursing	6/13 (46)	4/7 (57)	2/6 (33)
Nursing and personal care	7/13 (54)	3/7 (43)	4/6 (67)
Dementia registered	8/11 (73)	5/6 (83)	3/5 (60)
Dementia specialist	2/12 (17)	2/6 (33)	0/6 (0)
**Regular nursing home attendance from**			
Physiotherapist	7/12* (58)	4/7 (57)	3/5 (60)
Geriatrician	3/12 (25)	2/7 (29)	1/5 (20)
District Nurse	5/13 (38)	3/7 (43)	2/6 (33)
Tissue viability nurses	7/12 (58)	3/7 (43)	4/5 (80)
Dietitian	9/13 (69)	4/7 (57)	5/6 (83)
Speech and language therapist	8/13 (62)	4/7 (57)	4/6 (67)
Optician	11/13 (85)	5/7 (71)	6/6 (100)
Ophthalmologist	4/13 (31)	1/7 (14)	3/6 (50)
Chiropodist	13/13 (100)	7/7 (100)	6/6 (100)
Occupational therapist	7/13 (54)	3/7 (43)	4/6 (67)
Dentist	8/12 (67)	5/7 (71)	3/5 (60)
Audiologist	2/12 (17)	1/7 (14)	1/5 (20)
Dedicated GP for all residents	13/13 (100)	7/7 (100)	6/6 (100)

***for some variables N=12 where this data was not available for a particular home**

Better Health in Residents of Care Homes with Nursing - BHiRCH-NH study

Appendix 2. Family carer recruitment and retention

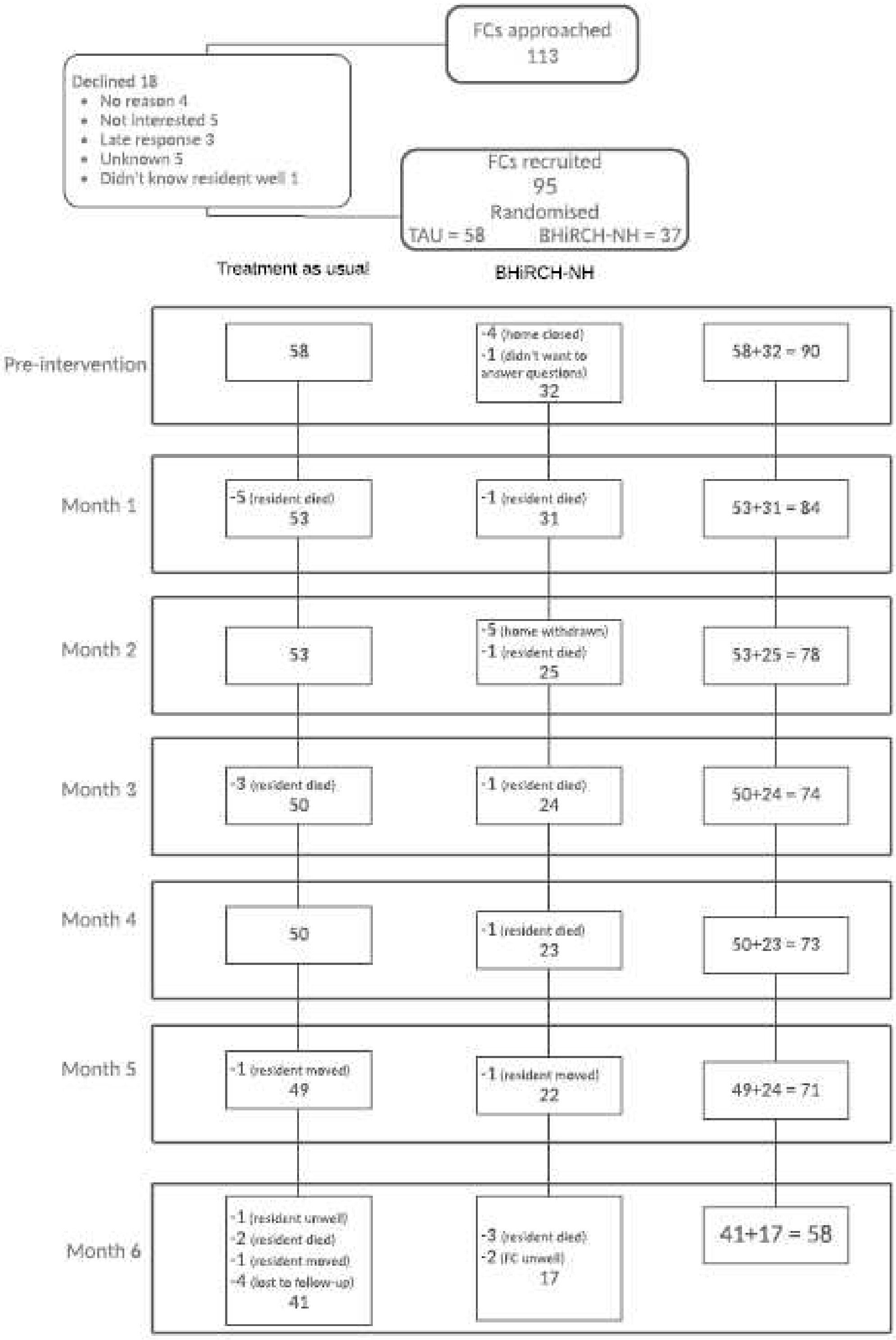

Better Health in Residents of Care Homes with Nursing - BHiRCH-NH study

Appendix 3. Staff recruitment and retention flowchart

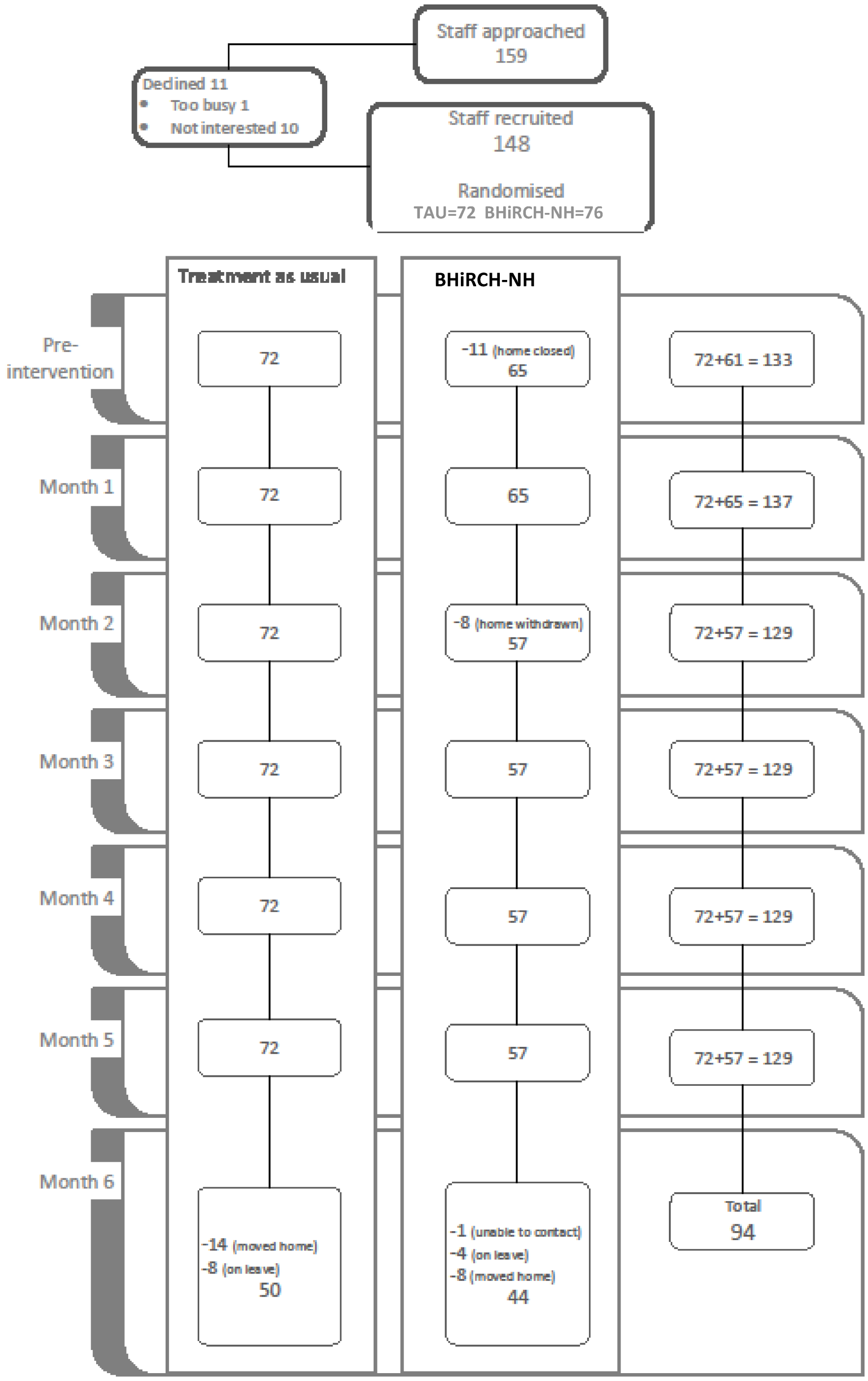

Better Health in Residents of Care Homes with Nursing - BHiRCH-NH study

Appendix 4: Staff participant characteristics pre-intervention

Characteristic	Cohort	TAU	Intervention
	n/N or median (% or IQR)		
**Demographics**	N=130	N=70	N=60
Male	16 (12)	9 (13)	7 (12)
Age	42 (30, 53)	42(32, 53)	39 (27, 50)
**Ethnicity**	N=129	N=69	N=60
White	76 (59)	32 (46)	44 (73)
Black	29 (22)	21 (30)	8 (13)
Asian	16 (12)	9 (13)	7 (12)
Other	8 (16)	7 (10)	1 (2)
**Marital status**	N=129	N=69	N=60
Married or cohabiting	71 (55)	40 (58)	31 (52)
Single	47 (36)	22 (32)	25 (42)
Divorced or widowed	11 (9)	7 (10)	4 (7)
**Education**	N=128	N=69	N=59
Completed years of education	11(11, 12)	12 (11, 12)	11 (11, 12)
No qualifications or GCSE or equivalent	16 (13)	9 (13)	7 (12)
A Level/NVQ/ HNC/ HND or equivalent	46 (36)	18 (26)	28 (47)
Degree or higher degree	49 (38)	28 (41)	21 (36)
Other qualification	17 (13)	14 (20)	3 (5)
**Type of staff**	N=125	N=67	N=58
Registered Nurse	24 (19)	9 (13)	7 (12)
Manager/ Matron/ Charge Nurse	12 (10)	8 (12)	4 (7)
Staff Nurse	2 (2)	5 (7)	3 (5)
Agency Nurse	87 (70)	1 (1)	1 (2)
Not a nurse		44 (66)	43 (74)
**Nurses only**	N=40	N=24	N=16
Works days	28 (70)	16 (67)	12 (75)
Works evenings	2 (5)	1 (4)	1 (6)
Works nights	9 (23)	4 (17)	5 (31)
Works mornings/early 12-hour shifts	12 (30)	5 (21)	7 (44)
Works afternoons/late 12-hour shifts	3 (8)	0 (0)	3 (19)
Works weekends	3 (8)	1 (4)	2 (13)
**Number of hours/week working in this nursing home**	N=40	N=24	N=16
Less than 8	1 (3)	0 (0)	1 (6)
8 to 12	0 (0)	0 (0)	0 (0)
13 to 40	13 (33)	8 (33)	5 (31)
More than 40	26 (65)	16 (67)	10 (63)
**How long have you worked at this nursing home?**	N=40	N=24	N=16
Less than 2 months	1 (3)	1 (4)	0
2 to 6 months	4 (10)	3 (13)	1 (6)
7 to 11 months	7 (18)	4 (17)	3 (19)
1 to 5 years	17 (43)	7 (29)	10 (63)
6 to 10 years	6 (15)	6 (25)	0
More than 10 years	5 (13)	3 (13)	2 (13)
**How long have you worked in nursing homes?**	N=39	N=24	N=15
7 to 11 months	2 (5)	1 (4)	1 (7)
1 to 5 years	14 (36)	6 (25)	8 (53)
6 to 10 years	7 (18)	7 (29)	0
More than 10 years	16 (41)	10 (42)	6 (40)
**How long have you been a registered nurse?**	N=40	N=24	N=16
1 to 5 years	8 (20)	3 (13)	5 (31)
6 to 10 years	10 (25)	7 (29)	3 (19)
More than 10 years	22 (55)	14 (58)	8 (50)

Better Health in Residents of Care Homes with Nursing - BHiRCH-NH study

**English is first language**	20 (50)	12 (50)	8 (50)
**PCAT Score**	49 (I46, 53)	50 (47,54)	48 (44,51)

Better Health in Residents of Care Homes with Nursing - BHiRCH-NH study

Appendix 5: Nurse – General Practitioner Communication Tool scores

	Pre-intervention			6 month follow up	
	Cohort	TAU	BHiRCH-NH n/N (%)	TAU	BHiRCH-NH
Have difficulty understanding what GP means due to medical jargon	2/40 (5)	2/24 (8)	0/16 (0)	0/14 (0)	0/7 (0)
A GP's language or accent makes it hard for you to understand what they are saying	2/40 (5)	1/24 (4)	1/16 (16)	0/15 (0)	0/7 (0)
A GP has difficulty understanding what you are saying due to your language or accent	2/40 (5)	2/24 (8)	0/16 (0)	0/15 (0)	0/7 (0)
GPs interrupt you before you have finished reporting on a resident	4/40 (10)	2/24 (8)	2/16 (13)	1/14 (7)	0/7 0)
GPs consider nurses' views when making decisions about residents	29/39 (10)	18/23 (78)	11/16 (69)	12/15 (80)	5/7 (71)
GPs are rude to you when you call them about a resident	2/39 (5)	2/23 (9)	0/16 (0)	1/15 (7)	0/7 (0)
You feel respected after an interaction with a GP?	29/40 (73)	19/24 (79)	10/16 (63)	12/15 (80)	4/7 (57)
You feel frustrated after an interaction with a GP?	4/40 (10)	3/24 (13)	1/16 (6)	1/15 (7)	0/7 (0)
Difficulty reaching the GP	9/40 (23)	5/24 (21)	4/16 (25)	2/15 (13)	2/7 (29)
Uncertainty about what to tell the GP	1/38 (3)	1/23 (4)	0/15 (0)	0/15 (0)	0/7 (0)
Feeling that the GP doesn't want to deal with the problem	3/40 (8)	1/24 (4)	2/16 (13)	1/15 (7)	1/7 (14)
Finding time to make the call	11/40 (28)	8/24 (33)	3/16 (19)	3/15 (20)	2/7 (29)
Finding a quiet place to make the call	10/40 (25)	7/24 (29)	3/16 (19)	4/14 (29)	0/7 (0)
Anticipating that the GP will be rude or unpleasant	1/40 (3)	0/24 (0)	1/16 (6)	1/15 (7)	0/7 (0)
Feeling hurried by the GP	7/40 (18)	4/24 (17)	3/16 (19)	1/15 (7)	0/7 (0)
Feeling that I am bothering the GP	4/39 (10)	2/24 (8)	2/15 (13)	2/15 (13)	0/7 (0)
Worrying that the GP may order something inappropriate or unnecessary	4/40 (10)	2/24 (8)	2/16 (13)	1/15 (7)	0/7 (0)
Feeling that I don't have enough time to say everything that I need to say	3/39 (8)	1/24 (4)	2/15 (13)	1/15 (7)	0/7 (0)
Nurses get good training in this care home	28/39 (72)	16/24 (67)	12/15 (80)	14/15 (93)	5/7 (71)
Nurses in this care home are willing to try new protocols	33/39 (85)	20/24 (83)	13/15 (87)	11/15 (73)	5/7 (71)
Nurses in this care home sometimes have to ignore protocols to get everything done	6/39 (15)	4/24 (17)	2/15 (13)	3/15 (20)	1/7 (14)
When this care home makes changes to improve resident care, they follow up to see if the changes worked	31/39 (79)	18/24 (75)	13/15 (87)	15/15 (100)	6/7 (86)
It is hard to make changes to improve resident care in this care home	4/39 (10)	1/24 (4)	3/15 (20)	2/15 (13)	0/7 (0)
This care home often wants nurses to follow protocols that don't really help residents	3/39 (8)	1/24 (4)	2/15 (13)	2/15 (13)	0/7 (0)
New protocols often make it harder for nurses to do their job	4/39 (10)	2/24 (8)	2/15 (13)	4/15 (27)	0/7 (0)
The communication between nurses and GPs in this care home is open	34/40 (85)	20/24 (83)	14/16 (88)	13/15 (87)	7/7 (100)
Feel comfortable communicating with GPs?	36/40 (90)	23/24 (96)	13/16 (81)	15/15 (100)	7/7 (100)
Feel comfortable communicating with nurse practitioners?	34/40 (85)	23/24 (96)	11/16 (69)	14/15 (93)	6/6 (100)

Better Health in Residents of Care Homes with Nursing - BHiRCH-NH study

Appendix 6: Nurse self- assessed core competencies

Core competencies	Pre-intervention			6 month follow up	
	Cohort	TAU	BHiRCH-NH	TAU	BHiRCH-NH
			n/N (%)		
**I know how acute episodes of chronic heart failure may manifest in older people**					
Disagree	0/34 (0)	0/21 (0)	0/13 (0)	0/15 (0)	0/7 (0)
Neither agree nor disagree	2/34 (6)	0/21 (0)	2/13 (15)	1/15 (7)	0/7 (0)
Agree	18/34 (53)	12/21 (57)	6/13 (46)	7/15 (47)	4/7 (57)
Completely agree	14/34 (41)	9/21 (43)	5/13 (38)	7/15 (47)	3/7 (43)
**I know how respiratory infections may manifest in older people**					
Disagree	0/34 (0)	0/21 (0)	0/13 (0)	0/15 (0)	0/7 (0)
Neither agree nor disagree	0/34 (0)	0/21 (0)	0/13 (0)	0/15 (0)	0/7 (0)
Agree	13/34 (38)	7/21 (33)	6/13 (46)	6/15 (40)	3/7 (43)
Completely agree	21/34 (62)	14/21 (67)	7/13 (54)	9/15 (60)	4/7 (57)
**I know how dehydration may manifest in older people**					
Disagree	0/34 (0)	0/21(0)	0/13 (0)	0/15 (0)	0/7 (0)
Neither agree nor disagree	0/34 (0)	0/21 (0)	0/13 (0)	0/15(0)	0/7 V
Agree	14/34 (41)	7/21 (33)	7/13 (54)	5/15 (33)	1/7 (14)
Completely agree	20/34 (59)	14/21 (67)	6/13 (46)	10/15 (67)	6/7 (86)
**I know how urinary tract infections may manifest in older people**					
Disagree	0/34 (0)	0/21 (0)	0/13 (0)	0/15 (0)	0/7 (0)
Neither agree nor disagree	0/34 (0)	0/21 (0)	0/13 (0)	0/15 (0)	0/7 (0)
Agree	15/3 4 (44)	9/21 (43)	6/13 (46)	5/15	1/7 (14)
Completely agree	19/34 (56)	12/21 (57)	7/13 (54)	10/15 (67)	6/7 (86)
**I am able to identify changes in physiology or behaviour as compared to baseline or normal for that resident**					
Disagree	0/34 (0)	0/21 (0)	0/13 (0)	0/15 (0)	0/7 (0)
Neither agree nor disagree	0/34 (0)	0/21 (0)	0/13 (0)	0/15 (0)	0/7 (0)
Agree	14/34 (41)	8/21(38)	6/13 (46)	5/15 (33)	0/7 (0)
Completely agree	20/34 (59)	13/21 (62)	7/13 (54)	10/15 (67)	7/7 (100)
**I am able to identify changes in physiology or behaviour for people who cannot communicate verbally**					
Disagree	0/34 (0)	0/21	0/13 (0)	0/15 (0)	0/7 (0)
Neither agree nor disagree	1/34 (3)	0/21(0)	1/13 (8)	1/15 (7)	0/7 (0)
Agree	17/34 (50)	11/21 (52)	6/13 (46)	6/15 (40)	2/7 (29)
Completely agree	16/34 (47)	10/21 (48)	6/13 (46)	8/15 (53)	5/7 (71)
**I know each resident's existing medical conditions**					
Disagree	1/34 (3)	0/21 (0)	1/13 (8)	0/15 (0)	1/7 (14)
Neither agree nor disagree	2/34 (6)	1/21 (5)	1/13 (8)	1/15 (7)	0/7 (0)
Agree	22/34 (65)	15/21 (71)	7/13 (54)	8/15 (53)	6/7 (86)
Completely agree	9/34 (26)	5/21 (24)	4/13 (31)	6/15 (40)	0/7 (0)
**I know each resident's care plans with respect to these medical conditions**					
Disagree	1/34 (3)	0/21 (0)	1/13 (8)	0/15 (0)	0/7(0)
Neither agree nor disagree	2/34 (6)	1/21 (5)	1/13 (8)	1/15 (7)	1/7 (14)
Agree	17/34 (50	10/21 (48)	7/13 (54)	8/15 (53)	5/7 (71)

Completely agree	14/34 (41)	10/21 (48)	4/13 (31)	6/15 (40)	1/7 (14)
**I know what is normal (i.e. their baseline) for each resident in terms of physiology, abilities and behaviour**					
Disagree	0/34 (0)	0/21 (0)	0/13 (0)	0/15 (0)	0/7 (0)
Neither agree nor disagree	1/34 (3)	1/21 (5)	0/13 (0)	2/15 (13)	0/7(0)
Agree	19/34 (56)	11/21 (52)	8/13 (62)	7/15 (47)	6/7 (86)
Completely agree	14/34 (41)	9/21 (43)	5/13 (38)	6/15 (40)	1/7 (14)
**I am able to carry out immediate interventions (eg adjusting fluid levels)**					
Disagree	1/33 (3)	1/21 (5)	0/12 (0)	0/15 (0)	0/7 (0)
Neither agree nor disagree	0/33 (0)	0/21 (0)	0/12 (0)	0/15 (0)	1/7 (14)
Agree	21/33 (64)	10/21 (48)	11/12 (92)	6/15 (40)	6/7(86)
Completely agree	11/33 (33)	10/21 (48)	1/12 (8)	9/15 (60)	0/7 (0)
**I am able to recognise verbal and nonverbal signs of acute deterioration**					
Disagree	0/34 (0)	0/21 (0)	0/13 (0)	0/15 (0)	0/7 (0)
Neither agree nor disagree	0/34 (0)	0/21 (0)	0/13 (0)	1/15 (7)	0/7 (0)
Agree	19/34 (56)	10/21 (48)	9/13 (69)	6/15 (40)	4/7 (57)
Completely agree	15/34 (44)	11/21 (52)	4/13 (31)	8/15 (53)	3/7 (43)
**I am able to carry out bedside observations (vital signs etc)**					
Disagree	0/34 (0)	0/21 (0)	0/13 (0)	0/15 (0)	0/7 (0)
Neither agree nor disagree	0/34 (0)	0/21 (0)	0/13 (0)	0/15 (0)	0/7 (0)
Agree	11/34 (32)	6/21 (29)	5/13 (38)	5/15 (33)	4/7 (57)
Completely agree	23/34 (68)	15/21 (71)	8/13 (62)	10/15(67)	3/7 (43)
**I am able to utilise basic clinical skills (use of medication etc)**					
Disagree	0/34 (0)	0/21 (0)	0/13 (0)	0/15 (0)	0/7 (0)
Neither agree nor disagree	0/34 (0)	0/21 (0)	0/13 (0)	0/15 (0)	0/7 (0)
Agree	11/34 (32)	5/21 (24)	6/13	5/15	3/7
Completely agree	23/34 (68)	16/21 (76)	7/13 (54)	10/15 (67)	4/7 (57)
I **know when to seek additional support (eg. asking a nurse, calling a GP or ambulance)**					
Disagree	0/34 (0)	0/21 (0)	0/13 (0)	0/15 (0)	0/7 (0)
Neither agree nor disagree	0/34 (0)	0/21 (0)	0/13 (0)	0/15 (0)	0/7 (0)
Agree	10/34 (29)	4/21 (19)	6/13 (46)	4/15 (27)	2/7 (29)
Completely agree	24/34 (71)	17/21 (81)	7/13 (54)	11/15 (73)	5/7 (71)
**I am able to set and monitor staff work to ensure quality of health care is provided**					
Disagree	1/34 (3)	1/21 (5)	0/13 (0)	1/15 (7)	0/7(0)
Neither agree nor disagree	0/34 (0)	0/21 V	0/13 V	0/15 (0)	0/7 (0)
Agree	14/34 (41)	5/21 (24)	9/13 (69)	4/15 (27)	3/7 (43)
Completely agree	19/34 (56)	15/21 (71)	4/13 (31)	10/15 (67)	4/7 (57)
**I am able to encourage or support other staff in their care for acute deterioration in resident's health**					
Disagree	0/34 (0)	0/21 V	0/13 (0)	0/15 (0)	0/7 (0)
Neither agree nor disagree	2/34 (6)	2/21 (10)	0/13 (0)	0/15 (0)	0/7 (0)
Agree	14/34 (41)	5/21 (24)	9/13 (69)	6/15 (40)	3/ (43)7
Completely agree	18/34 (53)	14/21 (67)	4/13 (31)	9/15 (60)	4/7 (57)
**I am able to negotiate and plan a course of action with other staff**					
Disagree	0/34 (0)	0/21 (0)	0/13 (0)	0/15 (0)	0/7 (0)
Neither agree nor disagree	4/34 (12)	3/21 (14)	1/13 (8)	1/15 (7)	0/7 (0)
Agree	13/34 (38)	6/21 (29)	7/13 (54)	5/15 (33)	5/7 (71)

Completely agree	17/34 (50)	12/21 (57)	5/13 (38)	9/15 (60)	2/7 (29)
**I am able to record a plan of action, e.g. draft and update care plans**					
Disagree	0/34 (0)	0/21 (0)	0/13 (0)	0/15 (0)	0/ (0)7
Neither agree nor disagree	0/34 (0)	0/21 (0)	0/13 (0)	1/15 (7)	0/7 (0)
Agree	16/34 (47)	10/21 (48)	6/13 (46)	4/15 (27)	3/7 (43)
Completely agree	18/34 (53)	11/21 (52)	7/13 (54)	10/15 (67)	4/7 (57)
**I am able to ensure acute changes in a resident's health status are communicated to staff in written or oral form**					
Disagree	0/34 (0)	0/21 (0)	0/13 (0)	0/15 (0)	0/7 (0)
Neither agree nor disagree	0/34 (0)	0/21 (0)	0/13 (0)	1/15 (7)	0/7(0)
Agree	14/34 (41)	7/21 (33)	7/13 (54)	5/15 (33)	3/7 (43)
Completely agree	20/34 (59)	14/21 (67)	6/13 (46)	9/15 (60)	4/7 (57)
**I am able to ensure effectiveness of treatment for acute changes in a resident's health status are communicated to staff and carers in written or oral form**					
Disagree	0/34 (0)	0/21 (0)	0/13 (0)	0/15 (0)	0/7 (0)
Neither agree nor disagree	2/34 (6)	1/21 (5)	1/13 (8)	1/15 (7)	0/7 (0)
Agree	12/34 (35)	6/21 (29)	6/13 (46)	6/15 (40)	2/7 (29)
Completely agree	20/34 (59)	14/21 (67)	6/13 (46)	8/15 (53)	5/7 (71)
**I am able to convey information about changes in health accurately and sensitively to carers**					
Disagree	0/34 (0)	0/21 (0)	0/13 (0)	0/15 (0)	0/6 (0)
Neither agree nor disagree	1/34 (3)	0/21 (0)	1/13 (8)	1/15 (7)	0/6 (0)
Agree	16/34 (47)	10/21 (48)	6/13 (46)	6/15 (40)	2/6 (33)
Completely agree	17/34 (50)	11/21 (52)	6/13 (46)	8/15 (53)	4/6 (67)
**I am able to adapt assessment and communication for people with dementia or communication difficulties**					
Disagree	1/34 (3)	1/21 (5)	0/13 (0)	0/15 (0)	0/7 (0)
Neither agree nor disagree	1/34 (3)	1/21 (5)	0/13 (0)	1/15 (7)	0/7 (0)
Agree	17/34 (50)	11/21 (52)	6/13 (460	6/15 (40)	3/7 (43)
Completely agree	15/34 (44)	8/21 (38)	7/13 (54)	8/15 (53)	4/7 (57)
**I am able to communicate the nature of change in a resident's health status**					
Disagree	0/34 (0)	0/21 (0)	0/13 (0)	0/15 (0)	0/7 (0)
Neither agree nor disagree	0/34 (0)	0/21 (0)	0/13 (0)	1/15 (7)	0/7 (0)
Agree	17/34 (50)	12/21 (57)	5/13 (38)	5/15 (33)	3/7 (43)
Completely agree	17/34 (50)	9/21 (43)	8/13 (62)	9/15 (60)	4/7 (57)

Better Health in Residents of Care Homes with Nursing - BHiRCH-NH study

Appendix 7. Serious adverse events categorised by type and potential relationship to the intervention

Type of serious adverse event (SAE)	TAU N=55* n/N (%)	Intervention N=41* n/N (%)
**Type of SAE**		
Death	17 (31)	14 (34)
Requires hospitalisation or prolonged hospital stay	37 (67)	25 (61)
Other medically significant	1 (2)	2 (5)
**"Expectedness" of SAE**		
SAE is expected side effect or possible outcome of the intervention as defined in the protocol (for example admission to hospital)	55 (100)	39 (95)
SAE is not expected	0 (0)	
Hernia		1 (2)
Choking		1 (2)
**Cause of SAE**		
SAE found by independent trial steering group not directly related to the intervention	55 (100)	41 (100)

Table footnote:

*denominator is number of serious adverse events not residents

†Serious adverse event is defined as per the UK Health Research Authority standard definition "an untoward occurrence that:

(a) results in death;

(b) is life-threatening;

(c) requires hospitalisation or prolongation of existing hospitalisation;

(d) results in persistent or significant disability or incapacity; and

(e) consists of a congenital anomaly or birth defect.

Better Health in Residents of Care Homes with Nursing - BHiRCH-NH study

Appendix 8. Resident retention flowchart

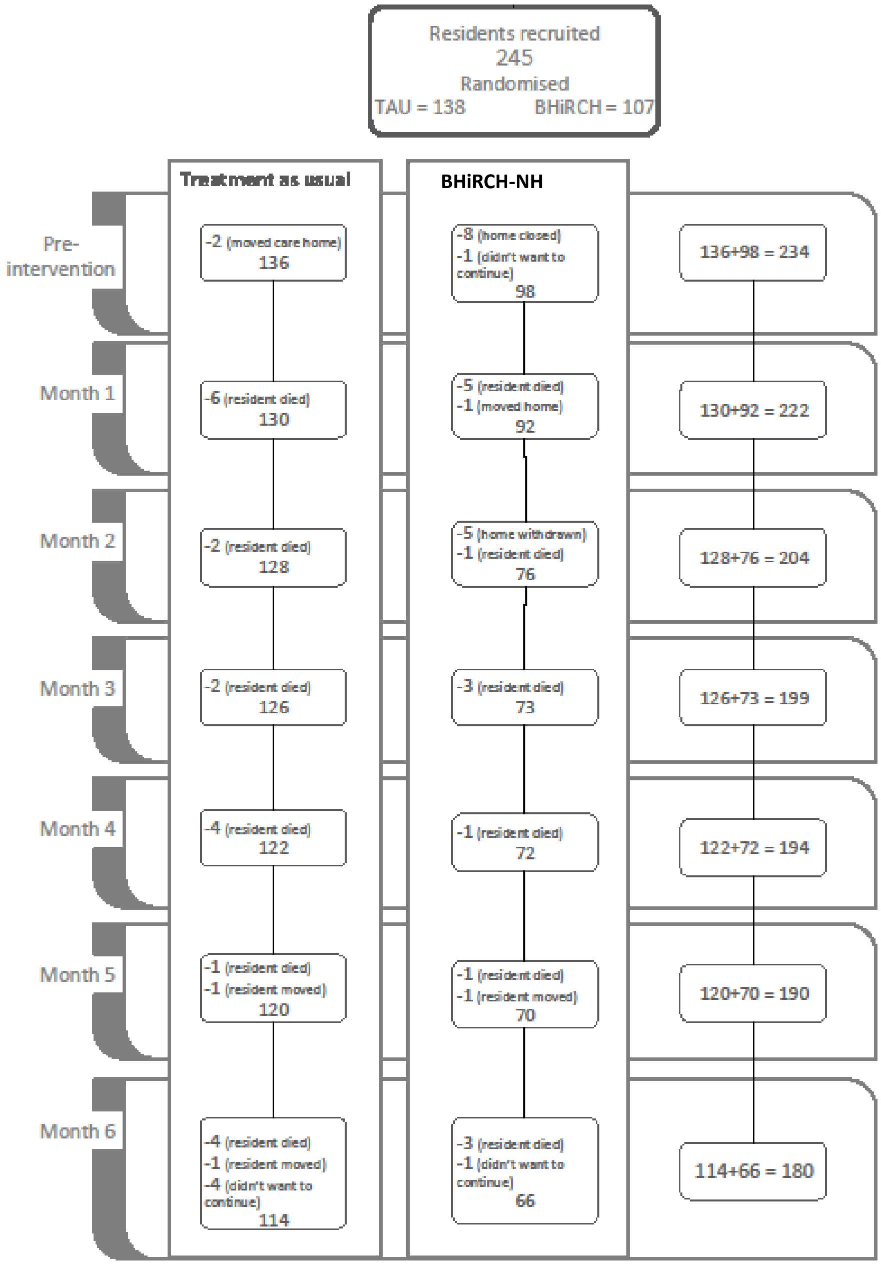

Better Health in Residents of Care Homes with Nursing - BHiRCH-NH study

Appendix 9. Mean healthcare resource use ± standard deviation (SD) and cost (unit costs at 2016/2017 values) per resident over the 6 months, complete data

Type of resource use (unit)	TAU	BHiRCH-NH	Difference (BHiRCH-NH vs TAU)		TAU	BHiRCH-NH	Incremental difference (BHiRCH-NH vs TAU)
	Mean ± SD	Mean ± SD	Mean	p-value	Mean cost	Mean cost	Mean cost (95% CI)
**Primary care, community health or emergency services (visits)**	8.3 ± 6.1 *(n=114)*	9.6 ± 7.6 *(n=71)*	1.3	0.20	£491.3	£694.9	£203.6 (-£67.4 to £474.6)
**Outpatient appointments (visits)**	0.4 ± 1.0 *(n=114)*	0.7 ± 1.5 *(n=68)*	0.3	0.11	£22.6	£49.9	£27.4 (£1.2 to £53.5)
**Inpatient services (admissions)**	0.2 ± 0.5 *(n=114)*	0.2 ± 0.6 *(n=68)*	0.001	0.99	£356.9	£426.6	£69.6 (-£333.8 to £472.9)
**Ambulance service (calls)**	0.2 ± 0.6 *(n=114)*	0.2 ± 0.5 *(n=68)*	0.04	0.61	£1.3	£1.6	£0.3 (-£0.9 to £1.4)
**Accident and Emergency (attendances)**	0.1 ± 0.5 *(n=114)*	0.3 ± 0.6 *(n=68)*	0.1	0.21	£23.1	£38.7	£15.6 (-£8.9 to £40.1)
**Prescriptions (packages)**	54.1 ± 23.8 *(n=114)*	60.5 ± 26.6 *(n=68)*	6.33	0.10	£325.7	£381.6	£55.9 (-£43.8 to £155.6)
**Length of stay in hospital (days)**	0.8 ± 3.9 *(n=114)*	0.9 ± 3.9 *(n=68)*	0.2	0.76			

Better Health in Residents of Care Homes with Nursing - BHiRCH-NH study

Appendix 10. Mean utility values and QALYs per resident (based on resident self-completed EQ-5D-5L questionnaire)

Utility values	TAU		BHiRCH-NH		Incremental difference	
	Mean	(95%CI)	Mean	(95%CI)	Mean	p-value
**Complete cases**						
Baseline	0.610 *(n=69)*	0.540 to 0.680	0.487 *(n=58)*	0.403 to 0.570	-0.123	0.02
6 months	0.685 *(n=59)*	0.610 to 0.759	0.614 *(n=32)*	0.501 to 0.727	-0.071	0.28
**QALYs**	0.337 *(n=46)*	0.297 to 0.377	0.259 *(n=26)*	0.196 to 0.321	-0.078	0.03
**With imputations**						
Baseline	0.617	0.607 to 0.628	0.504	0.491 to 0.518	-0.113	<0.001
6 months	0.652	0.641 to 0.662	0.649	0.637 to 0.661	-0.003	0.73
**QALYs**	0.317	0.312 to 0.322	0.289	0.283 to 0.294	-0.029	<0.001

Better Health in Residents of Care Homes with Nursing - BHiRCH-NH study

Appendix 11. Mean cost ± standard deviation (SD) of BHiRCH-NH and TAU of resource use per resident over 6 months

Type of costs	TAU	BHiRCH-NH	BHiRCH-NH vs. TAU
**BHiRCH-NH intervention costs per resident**			
Training	£0 ± £0	£39 ± £28	£39
Materials used in training	£0 ± £0	£0.20 ± £0.10	£0.20
Delivery of the intervention	£0 ± £0	£34 ± £32	£34
**Total costs (95%CI) per resident of healthcare resource use over 6 months**			
Complete cases	£1,250<br>(£900 to £1,599)<br>*(n=105)*	£1,560<br>(£912 to £2,209)<br>*(n=66)*	£310<br>(-£361 to £981)<br>*(n=171)*
With imputation	£1,233<br>(£1,171 to £1,295)	£1,458<br>(£1,351 to £1,566)	£225<br>(£108 to £342)
**Total costs (95%CI) per resident of BHiRCH-NH intervention cost + healthcare resource use over 6 months**			
Complete cases	£1,250<br>(£900 to £1,599)<br>*(n=105)*	£1,640<br>(£989 to £2,291)<br>*(n=66)*	£390<br>(-£283 to £1,063)<br>*(n=171)*
With imputation	£1,233<br>(£1,171 to £1,295)	£1,532<br>(£1,424 to £1,640)	£299<br>(£182 to £417)

Appendix 12. Cost-effectiveness acceptability curve showing the probability that BHiRCH-NH intervention is cost-effective versus TAU at different values of the WTP for a QALY, N=237

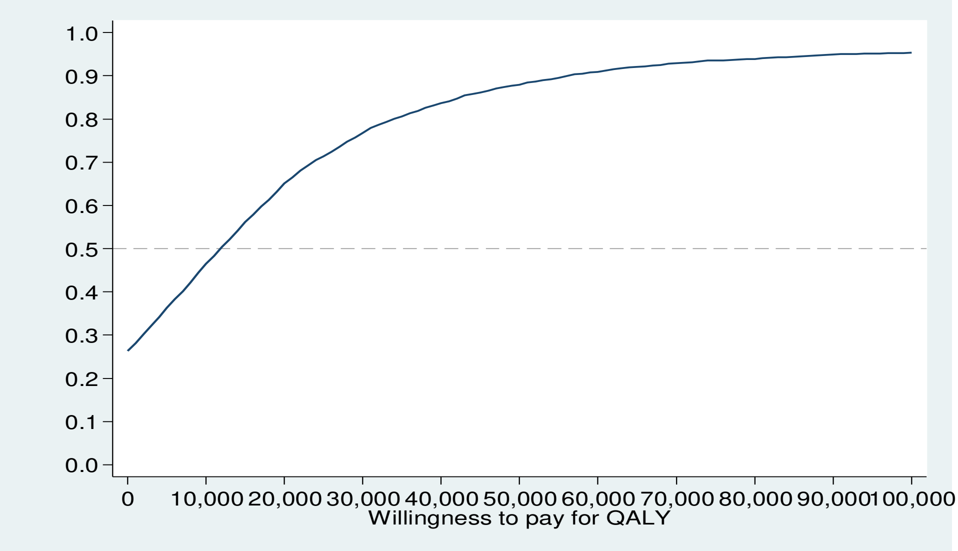

Figure 5 legend: Abbreviations: BHiRCH-NH-Better Health in Residents of Care Homes with Nursing study, QALY- Quality Adjusted Life year, TAU-Treatment as usual, WTP-Willingness to Pay
