# Supplementary material for: Pilot cluster randomised trial of an evidence-based intervention to reduce avoidable hospital admissions in nursing home residents (Better Health in Residents of Care Homes with Nursing—BHiRCH-NH Study)
Source: BMJ Open. 2020 Dec 13;10(12):e040732. doi: 10.1136/bmjopen-2020-040732 (PMC7737107; doi:10.1136/bmjopen-2020-040732)
Supplement: Author's manuscript [file bmjopen-2020-040732.draft_revisions.pdf]

BMJ Open is committed to open peer review. As part of this commitment we make the peer review history of every article we publish publicly available.

When an article is published we post the peer reviewers' comments and the authors' responses online. We also post the versions of the paper that were used during peer review. These are the versions that the peer review comments apply to.

The versions of the paper that follow are the versions that were submitted during the peer review process. They are not the versions of record or the final published versions. They should not be cited or distributed as the published version of this manuscript.

BMJ Open is an open access journal and the full, final, typeset and author-corrected version of record of the manuscript is available on our site with no access controls, subscription charges or pay-per-view fees (http://bmjopen.bmj.com).

If you have any questions on BMJ Open's open peer review process please email info.bmjopen@bmj.com

BMJ Open

A pilot cluster randomised trial of an evidence-based intervention to reduce avoidable hospital admissions in nursing home residents (Better Health in Residents of Care Homes with Nursing - BHiRCH-NH study)

Journal:	*BMJ Open*
Manuscript ID	bmjopen-2020-040732
Article Type:	Original research
Date Submitted by the Author:	21-May-2020
Complete List of Authors:	Sampson, Elizabeth; UCL, Division of Psychiatry<br>Feast, Alexandra; UCL, Division of Psychiatry<br>Blighe, Alan; University of Bradford Faculty of Health Studies, Ccentre for Applied Dementia Studies<br>Froggatt, Katherine ; Lancaster University Faculty of Health and Medicine, Faculty of Health and Medicine; Lancaster University<br>Hunter, Rachael; UCL, Research Dept of Primary Care and Population Health<br>Marston, Louise; UCL<br>McCormack, Brendan; Queen Margaret University School of Health Sciences, Nursing; Queen Margaret University School of Health Sciences,<br><br>Nurock, Shirley; UCL, Division of Psychiatry<br>Panca, Monica; University College London, Primary Care and Population Health<br>Powell, Catherine; University of Bradford Faculty of Health Studies, Centre for Applied Dementia Studies<br>Rait, Greta; UCL, Primary Care<br>Robinson, Louise; Newcastle University, Institute for Ageing, Institute for Health and Society<br>Woodward-Carlton, Barbara; University of Bradford Faculty of Health Studies, Ccentre for Applied Dementia Studies<br>Young, John; University of Leeds, Academic Unit of Elderly Care and Rehabilitation<br>Downs, Murna; University of Bradford, Bradford Dementia Group
Keywords:	GERIATRIC MEDICINE, PRIMARY CARE, HEALTH ECONOMICS

SCHOLARONE™
Manuscripts

BMJ

I, the Submitting Author has the right to grant and does grant on behalf of all authors of the Work (as defined in the below author licence), an exclusive licence and/or a non-exclusive licence for contributions from authors who are: i) UK Crown employees; ii) where BMJ has agreed a CC-BY licence shall apply, and/or iii) in accordance with the terms applicable for US Federal Government officers or employees acting as part of their official duties; on a worldwide, perpetual, irrevocable, royalty-free basis to BMJ Publishing Group Ltd ("BMJ") its licensees and where the relevant Journal is co-owned by BMJ to the co-owners of the Journal, to publish the Work in this journal and any other BMJ products and to exploit all rights, as set out in our licence.

The Submitting Author accepts and understands that any supply made under these terms is made by BMJ to the Submitting Author unless you are acting as an employee on behalf of your employer or a postgraduate student of an affiliated institution which is paying any applicable article publishing charge ("APC") for Open Access articles. Where the Submitting Author wishes to make the Work available on an Open Access basis (and intends to pay the relevant APC), the terms of reuse of such Open Access shall be governed by a Creative Commons licence – details of these licences and which Creative Commons licence will apply to this Work are set out in our licence referred to above.

Other than as permitted in any relevant BMJ Author's Self Archiving Policies, I confirm this Work has not been accepted for publication elsewhere, is not being considered for publication elsewhere and does not duplicate material already published. I confirm all authors consent to publication of this Work and authorise the granting of this licence.

For peer review only

A pilot cluster randomised trial of an evidence-based intervention to reduce avoidable hospital admissions in nursing home residents (Better Health in Residents of Care Homes with Nursing - BHiRCH-NH study)

Elizabeth L Sampson,[1,2] Alexandra Feast,[1] Alan Blighe,[3] Katherine Froggatt,[4] Rachael Hunter,[5] Louise Marston,[5] Brendan McCormack,[6] Shirley Nurock,[1] Monica Panca,[5] Catherine Powell,[7] Greta Rait,[5] Louise Robinson,[8] Barbara Woodward-Carlton,[3] John Young,[9] Murna Downs[3]

Corresponding author
Elizabeth L. Sampson
UCL Division of Psychiatry
6th floor, Wing B, Maple House
149 Tottenham Court Road
London
W1T 7NF
02076799500/internal 09500
e.sampson@ucl.ac.uk

[1] Marie Curie Palliative Care Research Department, Division of Psychiatry, University College London, UK.
[2] Barnet Enfield and Haringey Mental Health Trust Liaison Psychiatry Team, North Middlesex University Hospital, London, UK.
[3] Centre for Applied Dementia Studies, University of Bradford, Bradford, BD7 1DP, UK.
[4] International Observatory on End of Life Care, Lancaster University, Lancaster, UK.
[5] Department of Primary Care and Population Health and Priment Clinical Trials Unit, University College London, London, UK.
[6] Divisions of Nursing, Occupational Therapy & Arts Therapies, School of Health Sciences, Queen Margaret University, Edinburgh, UK.
[7]School of Pharmacy and Medical Sciences, University of Bradford, Bradford, UK
[8] Newcastle University Institute for Ageing and Institute for Health & Society, Newcastle University, Newcastle upon Tyne, UK.
[9]Academic Unit of Elderly Care and Rehabilitation, University of Leeds, Bradford Institute for Health Research, Bradford, UK.

Word count: 5113

For peer review only

ABSTRACT

Objectives: To pilot a complex intervention to support healthcare and improve early detection and treatment for common health conditions experienced by nursing home (NH) residents.

Design: Pilot cluster randomised controlled trial.

Setting: 14 NHs (7 intervention, 7 control) in London and West Yorkshire.

Participants: NH residents, their family carers and staff.

Intervention: Complex intervention to support healthcare and improve early detection and treatment of urinary tract and respiratory infections, chronic heart failure and dehydration, comprising: 1) "stop and watch (S&W)" early warning tool for changes in physical health, 2) condition-specific care pathway and 3) Situation, Background, Assessment and Recommendation tool to enhance communication with primary care. Implementation was supported by Practice Development Champions, a Practice Development Support Group and regular telephone coaching with external facilitators.

Outcome measures: Data on NH (quality ratings, size, ownership), residents, family carers and staff demographics during the month prior to intervention and subsequently, numbers of admissions, accident and emergency visits and unscheduled GP visits monthly for 6 months during intervention. We collected data on how the intervention was used, healthcare resource use and quality of life data for economic evaluation. We assessed recruitment and retention and whether a full trial was warranted.

Results: We recruited 14 NHs, 148 staff, 95 family carers and 245 residents. We retained the majority of participants recruited (95%). 15% of residents had an unplanned hospital admission for one of the four study conditions. We were able to collect sufficient questionnaire data (all over 96% complete). No NH implemented intervention tools as planned. Only 16 S&W forms and 8 Care Pathways were completed. There was no evidence of harm.

Conclusions: Recruitment, retention and data collection processes were effective but the intervention not implemented. A full trial is not warranted.

For peer review only - http://bmjopen.bmj.com/site/about/guidelines.xhtml

Trial registration: ISRCTN74109734 (registered 6/11/2017)

https://doi.org/10.1186/ISRCTN74109734

Original protocol: *BMJ Open*. 2019;9(5):e026510. doi:10.1136/bmjopen-2018-026510

Keywords: cost analysis; dehydration; dementia; geriatric medicine; heart failure; nursing homes; primary care; respiratory infections; urinary tract infections

ARTICLE SUMMARY

Strengths and limitations of this study

- The intervention was adapted from a successful USA programme, co-designed with residents, family carers and end-users to fit with the UK nursing home context
- We recruited and retained study participants and were able to collect sufficient data during the study
- The intervention was not implemented in practice, so we were not able to assess effectiveness

For peer review only - http://bmjopen.bmj.com/site/about/guidelines.xhtml

INTRODUCTION

Currently in the United Kingdom (UK) more than 420,000 people aged over 65 years live in residential care, of which approximately 220,000 reside in care home with nursing (referred to in this paper as “nursing homes” (NHs)).[1] Older people living in NHs have complex healthcare needs with high levels of multi-morbidity, frailty and dementia. The King’s Fund[2] and British Geriatrics Society[3] have raised concerns about the quality of healthcare provision to NHs.

There has been a 63% increase in all-cause hospital admissions from NHs between 2011-2015.[4] As well as causing distress to residents, their families and staff, hospitalisation is expensive for health and social care systems, costing an estimated £1.2 billion per annum in the UK.[5] Hospital admission increases the risk of decline in functional ability, delirium, adverse events and prolonged stays.[6 7]

Ambulatory Care Sensitive Conditions (ACSCs) are “conditions that can lead to unplanned hospital admissions that may have been avoidable or manageable by timely access to medical care in the community”.[8] The conditions include angina, asthma, cellulitis, chronic obstructive pulmonary disease (COPD), congestive heart failure (CHF), dehydration, diabetes mellitus, gastroenteritis, epilepsy, hypertension, hypoglycaemia, urinary tract infections (UTI), pneumonia, severe ear, nose, and throat infections.[9] Four ACSCs contribute to a 30% of hospitalisations from NHs:[5] respiratory infections;[10 11-13] acute exacerbation of chronic heart failure;[14 15] urinary tract infections;[12 16] and dehydration.[12]

A number of interventions have been developed to enhance healthcare in NHs, falling broadly into two categories; single component interventions (predominantly advance care planning or single disease care pathways, e.g. for pneumonia) and multicomponent interventions which include enhancing staff knowledge and skills,[17] clinical guidance and decision-support tools (care pathways), engaging with families,[18] and input from geriatricians

For peer review only - http://bmjopen.bmj.com/site/about/guidelines.xhtml

or nurse practitioners.[19] Research highlights the importance of collaborative development of interventions with staff[20], residents and families,[18] and using local champions to support implementation.[15]

"INTERACT" (Interventions to Reduce Acute Care Transfers), developed in the USA, focusses on managing acute changes in residents' condition and reduces transfers to acute hospitals.[15] We worked with stakeholders including staff and our family Carer Reference Panel (CRP), to develop and adapt INTERACT for use in the UK[21] (paper in preparation). The Promoting Action in Health Services (PARiHS) framework underpinned our co-design of implementation support and guidance[22] for the new "BHiRCH-NH" intervention.

Aim and objectives

Our aim was to indicate whether a definitive study of the BHiRCH-NH intervention is warranted.

Primary objective

To indicate whether the intervention was acceptable (including monitoring for serious adverse events (SAEs)) and feasible.

Secondary objectives

1. Establish whether consent procedures facilitate collection of sufficient individual-level data
2. Assess intervention fidelity
3. Assess the effectiveness of the implementation strategy and level of staff engagement with the intervention
4. Indicate whether the intervention would be sustainable outside the trial context
5. Assess potential primary and secondary outcome measures for a definitive trial

For peer review only - http://bmjopen.bmj.com/site/about/guidelines.xhtml

For peer review only

6. Measure completeness of data collection, documentation, return rate of questionnaires and assess potential primary and secondary outcomes for a definitive trial
7. Assess feasibility of collecting data for economic evaluation.

METHODS

Trial design

A pilot cluster randomised trial in NHs, conducted and reported as per the CONSORT guidance.[23] Detailed methods are described in the trial protocol paper.[21] The NH was the unit of allocation (seven intervention and seven control sites).

Patient and public involvement in research

The project was developed in collaboration with UK Dementia and Neurodegenerative Diseases Network (DeNDRoN) and Patient and Public Involvement representatives (SN and BW-C) who were grant co-applicants. Two Carer reference Panels (CRPs), ensured public involvement at all stages. Each comprised eight family carers of people with dementia and a person living with dementia, supported by Alzheimer's Society research volunteer network. They worked collaboratively on intervention design, recruitment and consent processes, accessibility of information leaflets, data collection, interpretation and dissemination.

Study population and eligibility criteria

Nursing homes

We recruited 14 NHs (8 in West Yorkshire and 6 in London) with adequate staffing to implement the intervention and support research. These were identified via local Clinical Research Networks, and the ENRICH (Enabling Research in Care Homes) Network, purposively selected to include a range of providers (large and small chains, independent providers), urban, suburban and rural. Managers, regional managers, or owners gave written permission. The intervention was an enhanced version of "usual care" implemented at NH

For peer review only

level. Therefore, individual consent was not required and all staff were involved in delivering the intervention to all care home residents.

Individual participants

We invited all English-speaking staff and residents over 65 years and their carers (family members or friends) to participate in individual-level data collection until we had recruited approximately 20 residents and 10 staff from each NH. We excluded residents receiving end-of-life care or those who did not wish to be involved in research.

Consent procedures

Ethical approval was given by the Queen Square London Research Ethics Committee (reference 17/LO/1542).

Residents

The care home manager or deputy manager identified all potentially eligible residents. If necessary, we conducted a capacity assessment regarding trial participation, adhering to the UK Mental Capacity Act (2005). If the resident lacked capacity, we used a personal consultee (friend or family), or if not available, a professional consultee (health or social care staff with a professional relationship to the resident but no connection with the project). If a resident lost capacity during the study a consultee was found.

Family carers and staff

We invited a family carer and NH staff associated with residents recruited to the study to answer questionnaires and they gave informed consent for this.

INTERVENTION

The pilot trial ran for 10 months (November 2017-August 2018). Sites were set up in months one and two, in month three we collected pre-intervention (baseline) data over four weeks

For peer review only - http://bmjopen.bmj.com/site/about/guidelines.xhtml

before the intervention was implemented. The intervention ran for 6 months with final follow up data collection and site closure occurring in month 10. We planned for the trial to run for 16 months from November 2017 but had to reduce follow-up due to delays in obtaining ethical approvals. Thus, timing of data collection differs from our protocol.[21]

Implementation support

Practice Development Champions' workshop

The research team delivered a one-day workshop to two PDCs from each NH comprising an introduction to four key ACSCs (respiratory and urinary tract infections, dehydration and acute exacerbation of chronic heart failure) and elements of how to bring about organizational change. We gave an overview of intervention materials.

Ongoing implementation support

1. We supported PDCs with a project handbook and the *Practice Development Workbook for Nursing, Health and Social Care Teams: Resources for Health and Social Care Teams*.[24]
2. We expected PDCs to establish a Practice Development Support Group (PDSG) to support their work in the NH. This "quality collaborative" approach[25] involves diverse stakeholders working together to close the gap between actual and potential practice.
3. We offered monthly telephone coaching to the PDCs by senior nurse researchers on our team (BMcC and KF).

The intervention (BHiRCH-NH)

This consisted of three key components, adapted from the INTERACT programme[26] and paper-based as UK NHs have variable use of electronic records:

1. *Stop and Watch Early Warning Tool*: Care assistants or nurses used this when they noted a change in a resident's condition. They circled observed changes, notified the nurse and placed the tool in the resident's NH records.

For peer review only - http://bmjopen.bmj.com/site/about/guidelines.xhtml

2. *Care Pathway*: This was a two-step clinical guidance and decision support system, focussing on symptoms and signs of four key ACSC conditions (acute exacerbation of chronic heart failure, urinary tract infections, dehydration). The initial “Primary” assessment comprised screening questions with the potential to trigger a more detailed “Secondary” assessment. If the Primary or Secondary Assessment result was ambiguous, the *Care Pathway* was to be administered at 6-hour intervals, until concerns had resolved and/or appropriate intervention was instigated. The nurse recorded the outcome of the Primary and Secondary Assessment and their care plan in the resident’s records and decided on the next course of action. This may have included further monitoring using the *Stop and Watch Early Warning Tool*, treatment initiated in the NH, or communication with primary care using the *SBAR*. Copies of the completed *Care Pathway* were kept with the resident’s record.
3. *The SBAR method:* A structured method for communicating critical information to primary care used by nurses to seek primary care intervention for the resident after the *Care Pathway* indicated a risk of decline.

Treatment as usual (TAU) nursing homes

Residents received usual care according to existing local policy and practice. We permitted all medications and treatments.

Data collection

Nursing homes

A Research Facilitator, employed by the NH provided pseudo-anonymised data at the NH level. We collected data for four weeks pre-intervention (baseline) including staff turnover and the number of beds available to new residents. For four weeks pre-intervention and then monthly for 6 months we documented the total number of contacts with GPs, ambulances, A&E attendances and hospital admissions.

For peer review only

Residents

We collected data pre-intervention on age, gender, ethnicity, marital status and highest level of education. Functional status was measured using the Barthel Index[27] pre-intervention, and at 6 months. We collected data for the month pre-intervention and then for the following 6 months on healthcare use and QoL data for health economic analysis, hospital admission overall and admissions for the four ACSCs of interest, ambulances called, out-of-hours GP visits or telephone contacts, A&E attendances and deaths.

Family carers

Pre-intervention we collected data on socio-demographics including age, gender, ethnicity and marital status and preferred role, i.e. how involved they would like to be with the resident's medical care.

Staff

For contextual understanding we documented pre-intervention; staff age, gender, and education level and characteristics of their work (qualifications, role, length of employment, shift pattern and first language). Pre-intervention and at 6 months we measured the extent to which they perceived the organisation supported person centred care (Organisational Support for Person-centred Care[28] (P-CAT)) and the Nurse Ratings of Communication with Primary Care Questionnaire.[29]

Outcomes

We collected data in three domains: 1) individual-level data on NH residents, their carers and staff where consent had been obtained, 2) system-level data collected by a Research Facilitator to provide pseudo-anonymised data at the NH level, and 3) process data collected by the study team (table 1).

For peer review only - http://bmjopen.bmj.com/site/about/guidelines.xhtml

Table 1: Summary of data collected, outcome measures and time schedule

Data collected and tool used		Pre-intervention	Monthly	6 months
Resident				
Socio-demographics	Age, gender, ethnicity, marital status, highest level of education	S	-	-
Service use in the prior month	Client Service Receipt Inventory (CSRI). Calculates service and total care costs	S	S	-
Functional status	The Barthel Index	S	-	S
Resident quality of life-self rated	EQ-5D-5L self-rated health index and visual analogue scale of current health state	P	-	P
Resident quality of life-proxy rated	EQ-5d-Proxy family carer or staff member view of the resident's QoL	FC/S	-	FC/S
Family carer				
Socio-demographics	Age, gender, ethnicity, marital status, years of schooling, highest level of education	FC	-	-
Quality of life	EQ-5D-5L	FC	-	FC
Preferred role	How much and how they like to be involved in the residents' care	FC	-	-
Staff				
Staff socio-demographics	Age, gender, ethnicity, number of years of education,	R	-	-
Staff work characteristics	Highest qualification, role in nursing home, length of service, shift pattern, first language	R	-	-
Organisational support for person-centered care	The Person-Centred Care Assessment Tool (P-CAT)	S	-	S
Communication with primary care	Nurse-GP Communication Needs Assessment Questionnaire	S	-	S
Perceived knowledge and skills for early detection in changes in health	Developed from feasibility study. Assesses key knowledge and skills needed to implement the intervention. Rated on 5-point Likert scale.	S	-	S
**System-level data**				
Number of hospital admissions	Respiratory infection, exacerbation of CHF, UTI and dehydration	S	S	-
"Avoidability" of admissions	Structured Implicit Record Review (SIRR; Saliba et al., 2000)	S	S	-
Use of Primary assessment tool	Respiratory infection, exacerbation of CHF, UTI and dehydration?	S	S	-
Use of Secondary assessment	Respiratory infection, exacerbation of CHF, UTI and dehydration	S	S	-
Out of hours GP contacts	GP visits or telephone contact	S	S	-
Ambulances and hospital use	Number of hospital admissions, A&E attendances and readmissions	S	S	-
Deaths in the last calendar month		S	S	-
Staff turnover		S	-	-
Nursing home occupancy level	Number of available beds to new residents	S	-	-

Measure assessed by: P, participant; FC family carer; R researcher; S, nursing home staff

For peer review only

Primary objective

To ascertain whether the intervention was acceptable and feasible we collected data monthly from the NH on intervention use in practice; the number of "Stop and Watch" Early Warning Tools and Primary (initial screening) and Secondary (more detailed) assessment tools completed. Where we had permission to collect individual-level resident data, we monitored participants monthly for SAEs defined as "any untoward occurrence that resulted in death, was considered life-threatening at the time of the event, required hospitalisation or prolongation of existing hospitalisation, resulted in persistent or significant disability or incapacity or was any other important medical condition".

Secondary objectives

1. *Establish whether consent procedures facilitate collection of sufficient individual-level data*

We collected data on consent and recruitment rates of residents, carers and staff.

2. *Assess intervention fidelity*

To explore intervention fidelity two nurse researchers aimed to review a convenience sample of five records for residents admitted to hospital, or received treatment in the NH, for ACSCs. They used a free-text review sheet to record references to trial intervention tools (Stop & Watch, the Care Pathway and SBAR) and assessed compliance with the care pathways. We noted where NHs made amendments to the structure or content of the care pathway.

3. *Assess the effectiveness of the implementation strategy and level of staff engagement with the intervention*

PDCs were offered a monthly support phone call with the research team to reflect on activities and achievements. They kept an activity log of work with Practice Development Support Groups to document the level of facilitation required to support the implementation process. Qualitative interviews were conducted to better understand the barriers and facilitators to implementation. Learning from the implementation strategy will be presented in a separate paper.

For peer review only - http://bmjopen.bmj.com/site/about/guidelines.xhtml

4. Indicate whether the intervention would be sustainable outside the trial context

Data from objectives 2-3 were considered by the independent project steering group at the end of the study.

5. Assess potential primary and secondary outcomes for a definitive trial

To explore the impact of enhanced healthcare in the NH we documented hospital admissions for the four key ACSCs (acute exacerbation of chronic heart failure, urinary tract infections, dehydration). Number (%) of residents requiring one or more ambulance calls, one or more unscheduled out of hours GP visits or phone calls and having one or more accident and emergency department visits were documented as potential secondary outcomes.

We tested the assumption that a hospitalisation for an ACSC was a proxy for an avoidable hospital admission using the Structured Implicit Record Review (SIRR) tool.[30] Two independent experts (geriatrician and community nurse) used the SIRR, which takes account of the resident's pre-existing health, any advance directives and the care options available at the time to assess the "avoidability" of the admission.

6. *Measure completeness of data collection and documentation, return rate of questionnaires*

We assessed completeness of outcome measures, data collection and return rate of questionnaires.

7. Assess feasibility of collecting data for economic evaluation

We collected data on resident service use with the Client Service Receipt Inventory[31] (CSRI), completed pre-intervention and then monthly for 6 months. Data on quality of life (QoL) was collected using the self-completed EQ-5D-5L[32] questionnaire where participants had the capacity to do this. Where a resident could not complete the EQ-5D-5L questionnaire, the proxy version was completed by the carer or a member of staff. We also collected data on carer QoL using the EQ-5D-5L questionnaire. All QoL data were collected pre-intervention and at 6 months.

For peer review only - http://bmjopen.bmj.com/site/about/guidelines.xhtml

Sample size

This was a pilot study so no sample size calculation was conducted. The number of care homes was chosen on pragmatic grounds to allow testing of study procedures and variability in settings.

Randomisation

NHs were randomised prior to intervention; 4 in West Yorkshire and 3 in Greater London (7 total) to the intervention and 4 in Yorkshire and 3 in Greater London (7 total) to "usual care", stratified by location. We used the SAS statistical programme to generate a randomisation list drawn up by a clinical trials unit statistician not otherwise involved in the study.

Blinding

This was not feasible for staff collecting data. Statisticians and health economists were blinded to allocation. The randomisation variable was supplied to them unlabelled, and the main analysis was completed using this.

Data management

All data was entered onto paper case report forms (CRFs) and then into an encrypted password protected database in accordance with the UK Data Protection Act and General Data Protection Regulation (GDPR). We followed a standardised process for database lock.

Statistical methods

As a pilot study, analyses are mainly descriptive (counts, means, standard deviation (SD), medians with interquartile range (IQR)) focussing on recruitment, participant characteristics, other baseline and outcome variables, loss to follow-up and tabulation of SAEs. We summarised completeness of data on outcome measures and questionnaire response rates.

For peer review only - http://bmjopen.bmj.com/site/about/guidelines.xhtml

Economic evaluation

Our aim was to provide a preliminary estimate of the costs, effects and relative cost-effectiveness of the intervention compared to TAU using accepted economic evaluation methods. We calculated costs associated with the intervention, including costs of staff training and implementation. Resource use associated with hospital admissions, primary care and other NHS and social care services were collected using the CSRI and costed[33-35] from the NHS/ personal social services (PSS) perspective. All costs are reported in 2016/2017 British Pounds (£). Quality-adjusted life years (QALYs) for residents were calculated using the EQ-5D-5L questionnaire and associated algorithms[36 37] mapping the 5L descriptive system data onto the 3L valuation set as recommended by the National Institute for Health and Care Excellence (NICE).[38 39] We report only results based on QALYs calculated using the resident self-completed EQ-5D-5L questionnaire as these had higher return. There was no discounting of costs or QALYs given that they were reported over 6 months only. Multivariate imputation by chained equation for missing data, generating 20 imputed data sets were used. For each of these, we ran 1,000 bootstrap replications using non-parametric bootstrapping. Bootstrap results were combined to calculate the mean values for costs and utilities and the standard errors around the imputed values, used to calculate 95% CI around point estimates. To report the probability that the intervention is cost-effective compared to TAU for a range of values of willingness-to-pay (WTP) for a QALY gained, the bootstrap results have been used to generate a cost-effectiveness acceptability curve (CEAC)[40] and the probability that the intervention is cost-effective compared to TAU at a £20,000 for a QALY gained reported.

RESULTS

Here, we give trial results with full data given in the supplementary file appendices to ensure transparency of reporting. In depth evaluation of implementation will be published separately.

For peer review only - http://bmjopen.bmj.com/site/about/guidelines.xhtml

Recruitment

Nursing homes

We recruited the target number of 14 NHs and randomised as planned; seven intervention (3 London, 4 Yorkshire) and seven control (3 London and 4 Yorkshire). One Yorkshire intervention NH dropped out during pre-intervention as it was closed by its owners. A further intervention NH in London dropped out following PDC training as they were unable to implement the intervention. Most NHs were privately managed with a median 50 residents (IQR 34, 68). The majority (73%) were "Dementia Registered". In terms of their CQC ratings, one home (7%) was "outstanding", eleven (79%) "good" and two (14%) "required improvement" (supplementary file, appendix 1).

Residents

We recruited 237 residents (figure 1), two thirds were female, predominantly white (90%) with a median age of 86 years (IQR 80, 91).

Figure 1 here

The median Barthel Index score was low at 27 (IQR 9, 64) indicating a high level of dependency in activities of daily living. Only six residents (3%) had an admission for an ACSC in the pre-intervention period, 11 (5%) residents had at least one ambulance called and 12 (5%) had an unscheduled GP visit or telephone contact (table 2).

Family carers

We recruited 91 family carers, two thirds were female (supplementary file, appendix 2). Median age was 63 years (IQR 57, 71). Most (91%) wished to be involved in noticing early changes in the resident's health.

Table 2: Characteristics of residents and family carers

Characteristic	Cohort	TAU	BHiRCH-NH
	n or median (% or IQR)		
**RESIDENTS**			
**Demographics**	N=234	N=137	N=97
Male	73 (31)	46 (34)	27 (28)
Age	86 (80,91)	86 (80, 91)	84(78, 91)
**Ethnicity**	N=225	N=131	N=94
White	203 (90)	117 (89)	86 (91)
Black	14 (6)	9 (7)	5 (5)
Asian	5 (2)	4 (3)	1/(1)
Other	3 (1)	1 (1)	2(2)
**Marital status**	N=223	N=127	N=96
Married or cohabiting	49 (22)	28 (22)	21 (22)
Single	59 (26)	40 (32)	19 (20)
Divorced or widowed	115 (52)	59 (46)	56 (58)
**Education**	N=184	N=133	N=71
Completed years of education	11 (9,12)	11(10, 12)	11(9, 11)
No qualifications or GCSE or equivalent	107 (58)	63 (56)	44 (62)
A Level/NVQ/ HNC/ HND or equivalent	18 (10)	11 (10)	7 (10)
Degree or higher degree	23 (13)	14 (12)	9 (13)
Other qualification	36 (20)	25 (22)	11 (15)
**Function**			
Barthel Index score	27 (9, 64)	27(9, 66)	30(8, 63)
**CARERS**			
**Demographics**	N=91	N=56	N=35
Male	31 (34)	17 (30)	14 (40)
Age	63 (57, 71)	62(57, 71)	64(58, 74)
**Ethnicity**	N=87	N=52	N=35
White	72 (83)	43 (83)	29 (83)
Black	12 (14)	7 (13)	5 (14)
Asian	3 (3)	2 (4)	1(3)
**Marital status**	N=87	N=53	N=34
Married or cohabiting	65 (75)	36 (68)	29 (85)
Single	10 (11)	8 (15)	2 (6)
Divorced or widowed	12 (14)	9 (17)	3 (9)
**Education**	N=86	N=53	N=33
Completed years of education	11 (11,12)	12(11, 13)	11(11, 12)
No qualifications or GCSE or equivalent	35 (41)	21 (40)	14 (42)
A Level/NVQ/ HNC/ HND or equivalent	13 (15)	9 (17)	4 (12)
Degree or higher degree	26 (30)	14 (26)	12 (36)
Other qualification	12 (14)	9 (17)	3 (9)
**Preferred role**	N=87	N=52	N=35
Noticing early signs of changes in health	79 (91)	49 (94)	30 (86)
Informing staff about early signs of changes in health	77 (89)	48 (92)	29 (83)
Educating staff about how early signs of changes in health present	51 (59)	28 (54)	23 (66)
Educating care staff about health history of their family member	57 (66)	33 (63)	24 (69)
Prefer not to be involved	5 (6)	2 (4)	3 (9)
Other	18 (21)	9 (17)	9 (26)

For peer review only - http://bmjopen.bmj.com/site/about/guidelines.xhtml

Staff

We recruited 132 staff (supplementary file, appendix 3), with a median age of 42 years (IQR 30, 53), 12% were male, 50% of nurses spoke English as a first language and 59% of staff described themselves as white. Most staff (71%) had worked at the care home for a year or more and 30% were qualified nurses (supplementary file, appendix 4). Scores on the P-CAT scale (possible range 13-65) were generally positive with a median score of 49 (IQR 46, 53) (supplementary file, appendix 4). Most nurses were positive about the quality of communications they had with GPs (supplementary file, appendix 5) and their self-rated knowledge and skills (supplementary file, appendix 6).

Primary objective-feasibility and acceptability

Use of the intervention

Across the five intervention NHs, only sixteen Stop and Watch forms were completed of which eleven came from a single NH. Eight Care Pathways were reported as completed but only three were located by the study team. There was a median of one Stop and Watch form (IQR 0,3) and a median of zero Care Pathways (IQR 0,2) completed per month. In a few cases, routine clinical observations (e.g. temperature, blood pressure etc.) were carried out, but were not reported systematically or presented as part of a coherent assessment plan. One home had a policy of recording routine observations once per month.

Serious adverse events

There were no differences in SAEs between TAU and intervention groups. There were 104 SAEs in 74 residents during the study and 33 residents died (19 TAU and 14 intervention). Of the 104 SAEs, hospitalisation (any cause) was the most common (N= 50) (supplementary file, appendix 7).

For peer review only - http://bmjopen.bmj.com/site/about/guidelines.xhtml

Secondary objectives

1. Resident consent procedures and collection of sufficient individual level data

We screened 680 residents, 557 met eligibility criteria and 245 were recruited (35% recruitment). Taking into account that two NHs dropped out, leaving 12 in the study, our target of 240 residents (20 residents per care home) was reached. Most eligible residents (364, 65%) did not have capacity to consent to participate in the study. Of recruited residents 73% completed the study (supplementary file, appendix 8).

2. Assess intervention fidelity

It was not possible to assess fidelity to the intervention as the intervention was not implemented as intended and the documentation required to assess fidelity was not available.

3. Assess the effectiveness of the implementation strategy and level of NH staff engagement with the intervention

The implementation strategy was not effective and NH staff did not engage with or use the intervention tools. Data and learning from implementation will be presented in a separate paper.

4. Investigate whether the intervention would be sustainable outside the context of a trial

The intervention was not widely used; 16 Stop and Watch forms were completed and we found eight completed Care Pathway documents. We concluded the intervention in its current form was not sustainable.

5. Assess potential primary and secondary outcome measures for a definitive trial

Rates of hospitalisation for ACSCs

At baseline the rates of hospitalisation for ACSCs (respiratory infection, exacerbation of CHF, UTI and dehydration), our potential primary outcome, were low and 0.4% of the cohort

For peer review only - http://bmjopen.bmj.com/site/about/guidelines.xhtml

had an admission for respiratory infections, 1% for UTI, none for dehydration and 0.4% had an admission for chronic heart failure in the month before the trial started. There were six admissions in total from the 235 residents in the study. Considering the whole six-month study period, 25 study participants (15%) had an unplanned hospital admission for one of the ACSCs. The low rates of unplanned hospital admission (per 100 person months) for these ACSCs suggest this is not an optimal primary outcome measure for a future study (table 3). Considering secondary outcome measures for a definitive trial, we found a slightly higher proportion of participants (n=38, 16%) had an accident or emergency attendance during the follow up period, 42 (18%) had at least one ambulance called, 29 (12%) had an unscheduled (out-of-hours) GP visit and 21 (11%) died. The incidence of these events was still relatively low and not sufficiently frequent for a definitive study outcome.

Hospitalisation for an ACSC as a proxy for avoidable hospital admission

We intended to use the Structured Implicit Record Review (SIRR) tool to assess the appropriateness of 30 resident admissions for one of the four ACSCs, but it was not always possible to identify the reasons for admission and these were low overall. We therefore expanded our sample to include any hospital admission for any cause. One NH had no eligible residents, because those admitted to hospital had died and/or their care records were no longer available. We therefore also included residents who died in hospital, as long as their records were still available. We were able to assess 10 admissions from a total of 24 which occurred during the study period. Using the SIRR tool, three of the ten admissions assessed were deemed to be potentially avoidable. None of the NH care records provided a complete picture of residents' health in the period leading up to admission. From this review an ACSC admission was not a reliable proxy measure for an "avoidable admission".

For peer review only

Table 3. System-level outcome data

	Pre-intervention			Over 6 month follow up period	
	Whole cohort	TAU	BHiRCH-NH	TAU	BHiRCH-NH
	n or median (% or (IQR)				
**Study cohort**	N=235	N=139	N=96	N=139	N=96
At least one admission in the last month	6 (3)	2 (1)	4 (4)	19 (14)	16/96
Respiratory infection admission	1 (0.4)	0	1 (1)	8 (6)	5/96
Urinary tract infections admission	2 (1)	1 (1)	1 (1)	5 (4)	2/96
Dehydration admission	0	0	0	0	1/96
Congestive heart failure admission	1 (0.4)	0	1 (1)	0	1/96
At least one ambulance called	11(5)	4 (3)	7 (7)	20 (14)	22/96
At least one out of hours GP visit or telephone contact	12 (5)	11 (8)	1 (1)	14 (10)	15/96
At least one Accident and Emergency attendance	10 (4)	6 (4)	4 (4)	17 (12)	21/96
Died	1 (0.4)	1 (1)	0		
**Care home data**					
Number hospital admissions	3 (2, 5)	4 (2, 7)	3 (2, 4)	12 (12, 16)	12 (7, 18)
Number of ambulances called	3 (2,6)	4 (2,9)	3 (2, 6)	12 (11,17)	19 (7, 22)
Unscheduled (out of hours) GP visits or telephone contacts	1 (1,3)	2 (1,3)	1 (1, 3)	8 (7,13)	9 (4, 25)
Accident and Emergency attendances	3 (2,5)	3 (1,4)	3 (2, 6)	12 (11,13)	8 (7, 13)
Rate of hospital admissions per 100 person months		-	-	5.3 (2.3, 8.3)	5.9 (1.7, 7.1)
Rate of ambulances called per 100 person months		-	-	5.7 (2.3, 8.0)	6.0 (2.0, 9.3)
Rate of unscheduled (out of hours) GP visit or contacts per 100 person months		-	-	2.5 (1.8, 4.0)	5.1 (1.1, 6.0)
Rate of Accident and Emergency attendances per 100 person months		-	-	4.3 (2.5, 8.3)	3.9 (2.0, 5.6)

6. Completeness of data collection and documentation, return rate of questionnaires

Care staff-related data

We collected data on most recruited care staff at baseline (N=132). For example, 129 care staff gave demographic detail (98%) and 127 (96%) completed the P-CAT scale. Attrition in response to these scales was secondary to NHs dropping out of the study, rather than staff being unwilling or unable to complete the forms.

Resident-related data

Demographic and functional ability data were available on most residents at baseline (N=235) including gender (99%), ethnicity (95%), marital status (98%) and functional ability (Barthel Index) (98%). We cannot be sure that no admissions or visits to acute hospital were

For peer review only - http://bmjopen.bmj.com/site/about/guidelines.xhtml

missed as NHs did not have centralised systems for collecting these data.

7. Cost and outcome data for use in an economic evaluation, key cost components and probability of cost effectiveness

One NH withdrew after randomisation and therefore, we considered the cost of intervention to be £0 (as "per randomised" approach). Assuming the intervention is offered to all 89 residents randomised to the intervention group, the mean cost per resident would be £74 (95%CI £64 to £84). During follow-up, there were no significant differences in the majority of the components of healthcare resource use between the intervention and TAU groups (supplementary file, appendices 9and 10). Differences in mean utility values and QALYs were not statistically significant when carers assessed their QoL and therefore, no further analysis has been performed. The mean total cost of healthcare resource use per resident over 6 months was £1,458 (95%CI £1,351 to £1,566) in the intervention group and £1,233 (95%CI £1,171 to £1,295) in the TAU group (supplementary file, appendix 11). Non-parametric bootstrapping after multiple imputation produced a mean total cost per resident in the intervention group of £1,479 (95%CI £757 to £2,200), compared with £1,271 (95%CI £975 to £1,566) in the TAU group. The mean difference in cost between the BHiRCH-NH and TAU group was £208 (95%CI -£561 to £977). Non-parametric bootstrapping after multiple imputation produced 0.315 (95%CI 0.304 to 0.326) QALYs in the intervention group and 0.298 (95%CI 0.290 to 0.307) QALYs in the TAU group, generating a mean difference in QALYs of 0.016 (95%CI 0.003 to 0.300) which was statistically significant (table 4).

For peer review only - http://bmjopen.bmj.com/site/about/guidelines.xhtml

Table 4. Cost-effectiveness of BHiRCH-NH intervention versus TAU: complete case and imputed data analyses

	Incremental cost		QALY gained	
	Mean	(95% CI)	Mean	(95% CI)
Base case*	£208	-£561 to £977	0.016	0.003 to 0.300
Complete case^	£352	-£745 to £1,448	0.018	-0.012 to 0.048

*Data include values imputed using multiple imputation with standard errors corrected to account for uncertainty in the imputed values. QALYs gained are adjusted for baseline utility values and care home clustering. The incremental costs are adjusted for costs in the 1 month period prior to baseline and care home clustering. ^As for the base case analysis except there is no multiple imputation for missing data

The incremental cost per QALY gained of BHiRCH-NH versus TAU was £12,633. Residents receiving the intervention accrued a non-significantly higher cost, and a very small increase in QALYs; the intervention has a 65% probability of being cost-effective at a WTP of £20,000 (supplementary file, appendix 12).

DISCUSSION

Our cluster randomised pilot trial of the BHiRCH-NH intervention in 12 NHs found our study processes were effective. We successfully recruited, retained and obtained individual-level data from residents, staff and family carers. Data on adverse events did not suggest the intervention caused harm. Our Carer Reference Panel worked with us throughout, advising on study set-up, engaging with homes and potential participants, data collection and contributing to data analysis and interpretation. However, despite excellent recruitment and retention, there was limited engagement with the intervention tools and the support offered for their implementation. The lack of use of the intervention coupled with the economic analysis means that we would not recommend a definitive randomised controlled trial of the BHiRCH-NH intervention. We focussed on four key ACSCs (pneumonia, dehydration, urinary tract infection and exacerbation of chronic heart failure) because they are common causes of potentially avoidable hospital admissions and a significant area of health policy focus. There is lack of national comparative data, but we found lower hospitalisation rates for

For peer review only - http://bmjopen.bmj.com/site/about/guidelines.xhtml

these conditions than we expected. Categorising admissions to hospital is complex and people may present with broader symptoms i.e. delirium or falls. ACSCs may be more of an administrative label to be used in large data analyses rather than a sensitive tool for assessing the “avoidability” or otherwise of acute hospital admissions.

Implementing practice change in NHs can be challenging. Whilst the INTERACT programme, from which our BHiRCH-NH intervention was adapted, demonstrated reductions in all-cause hospitalisations among actively participating NHs, a subsequent larger randomised controlled implementation trial in 85 USA NHs had no effect on emergency department visits or hospital admissions.[15] Resources, competing demands and instability of NH leadership were all barriers to successful to INTERACT implementation[41] and it is likely that these factors were also present in our study.

Our study has limitations. We recruited NHs, residents, staff and family carers to target, although two NHs dropped-out from the intervention arm. Our NHs may be atypical as 79% were rated as “good” by the CQC, compared to the UK national average of 73%. Residents in our sample were broadly representative of the UK NH population in terms of age and gender.[42] Monthly visits from research fieldworkers and the appointment of Research Facilitators who were existing NH staff fostered positive relationships between NHs and the research team and facilitated access to NH records. We cannot verify that data on hospital attendances, staffing and support from external health care services is complete, as most NHs did not routinely record hospital admissions or ambulance and GP call-outs. Staff self-rated their knowledge and skills regarding health conditions and their communication with primary care and research assistants collecting outcome data were not blind to NH allocation. We assumed we could use admission for ACSCs as a proxy for avoidable admissions, but even using the SIRR tool[43] it was difficult to identify whether an admission was for an ACSC or not. Future studies should include consent to access hospital records so this can be more thoroughly assessed.

For peer review only - http://bmjopen.bmj.com/site/about/guidelines.xhtml

Current UK policy focus on reducing hospitalisations and enhanced health care in NHs has led to significant levels of activity in local health and social care services. Research in such a fast-moving landscape is challenging. NHS England's demonstration projects "Vanguards" and increasing numbers of local quality improvement initiatives means "usual care" will be improving, making trials challenging to conduct. Although our intervention was co-designed with staff, this involved an extra burden of observation and documentation, which was difficult for staff who were already pressed for time. Recent studies conducted in care homes suggest that a higher level of support and facilitation may be required for successful implementation. The Palliative Care for Older People Steps to Success Programme (PACES), conducted in 78 NHs across seven countries, which involved a complex intervention to improve end of life care, did not improve resident comfort or staff knowledge despite using a train-the-trainer approach.[44] However, the Well-Being and Health for People with Dementia (WHELD) programme was successfully implemented in 69 UK care homes and showed a significant effect on reducing agitation possibly through a much higher intensity of external facilitation that may not be sustainable or cost effective.[45] Since we designed our intervention and conducted our pilot trial, knowledge on improving healthcare for care home residents has increased. Key components for resident health care, identified in the 2017 "Optimal" study were GP involvement supported by integrated external services.[46] These findings are reflected by emerging evidence that change to the wider health and social care system may be more effective in reducing the number of NH residents admitted to hospital.[47]

CONCLUSIONS

Our co-designed complex intervention of tools for early detection, monitoring and communication of change in residents' health does not warrant a future definitive trial because it was not implemented in practice. However, it was important that this pilot trial was conducted as this has avoided the risk of embarking on a full trial and subsequent waste of

For peer review only - http://bmjopen.bmj.com/site/about/guidelines.xhtml

resources and time.[48] Our study contributes to learning in the under-researched but vital field of NH research.

DECLARATIONS

Acknowledgements: The authors would like to thank the members of our carer reference panels and the Alzheimer's Society research network volunteers. We are grateful to the nursing homes that supported our feasibility study. We are grateful to the Clinical research Network staff; Mary-Jo Doyle, Deborah Cooper, Kim Williams, Sally Gordon and Helen Permain and others who assisted with data collection Justin Chan, Ayesha Dar, Luiza Grycuk, Dr Adam Hughes, Dr Maria Ivanov and Angela Richardson. We would like to thank the study steering group (Dr Najma Siddiqi (Chair), Prof Tom Dening, Bert Green, Mike Bradburn, Laura Mandefield and members of the international advisory group (Prof Finbarr Martin (Chair), Prof Barbara Bowers, Prof John Gladman, Prof Claire Goodman, Prof Martin Green, Prof Raymond Koopmans, Prof Julienne Meyer, Prof Des O'Neill, Prof Jo Ouslander, Dr Hilary Rhoden, Jenny Adams, Prof Lynn Chenoweth, Prof Graham Stokes, Louise Taylor, Gavin Terry, Steve Williams). .

Competing interests statement: none declared

Funding: This work was supported by the UK NIHR grant number RP-PG-0612-20010. The views expressed are those of the author(s) and not necessarily those of the NHS, the NIHR or the Department of Health and Social Care. The funding body had no role in the design of the study, collection, analysis, and interpretation of data and in writing the paper.

Author contributions: ELS, AF, AB, KF, RH, LM, BMcC, SN, CP, MP, FC, GR, LR, BW-C, JY, MD were involved in development of the intervention and made substantial contributions to the concept and design of the study and protocol. LM, MP and RH analysed the data.

w only

For peer review only - http://bmjopen.bmj.com/site/about/guidelines.xhtml

ELS and AF wrote the paper with assistance from CP. ELS, AF, AB, KF, RH, LM, BMcC, SN, CP, MP, FC, GR, LR, BW-C, JY, MD critically revised the manuscript and approved the final version.

Provenance and peer review: not commissioned; externally peer reviewed

Data sharing: The datasets generated and/or analysed during the current study are not publicly available, the small number of NHs means that data may be identifiable. Data are available from the corresponding author on reasonable request.

peer review only

For peer review only - http://bmjopen.bmj.com/site/about/guidelines.xhtml

REFERENCES

1. Laing and Buisson. Care of Older People UK Market Report. 28th ed, 2017.
2. Tian Y, Dixon A, Gao H. Emergency hospital admissions for ambulatory care-sensitive conditions: identifying the potential for reductions: King's Fund, 2012.
3. British Geriatric Society. Quest for quality in care homes: British Geriatrics Society Joint Working Party Inquiry into the Quality of Healthcare Support for Older People in Care Homes: A Call for Leadership, Partnership and Quality Improvement. London: British Geriatric Society, 2011.
4. Bardsley M, Blunt I, Davies S. Is secondary preventive care improving? Observational study of 10-year trends in emergency admissions for conditions amenable to ambulatory care. *BMJ Open* 2013;3(1) doi: 10.1136/bmjopen-2012- 002007
5. Kings Fund. Data briefing: emergency hospital admissions for ambulatory care sensitive conditions 2012 [Available from: https://www.kingsfund.org.uk/sites/default/files/field/field_publication_file/data-briefing-emergency-hospital-admissions-for-ambulatory-care-sensitive-conditions-apr-2012.pdf accessed 20/02/2020.
6. Mukadam N, Sampson EL. A systematic review of the prevalence, associations and outcomes of dementia in older general hospital inpatients. *IntPsychogeriatr* 2010:1-12.
7. Watkin L, Blanchard MR, Tookman A, et al. Prospective cohort study of adverse events in older people admitted to the acute general hospital: risk factors and the impact of dementia. *IntJGeriatrPsychiatry* 2012;27(1):76-82. doi: 10.1002/gps.2693 [doi]
8. Carter MW, Porell FW. Vulnerable populations at risk of potentially avoidable hospitalizations: the case of nursing home residents with Alzheimer's disease. *American journal of Alzheimer's disease and other dementias* 2005;20(6):349-58. [published Online First: 2006/01/07]
9. Grabowski DC, O'Malley AJ, Barhydt NR. The costs and potential savings associated with nursing home hospitalizations. *Health affairs (Project Hope)* 2007;26(6):1753-61. doi: 10.1377/hlthaff.26.6.1753 [published Online First: 2007/11/06]
10. Purdy S, Griffin T, Salisbury C, et al. Ambulatory care sensitive conditions: terminology and disease coding need to be more specific to aid policy makers and clinicians. *Public Health* 2009;123:169-73.
11. Walsh E, Wiener J, Haber S. Potentially avoidable hospitalizations of dually eligible Medicare and Medicaid beneficiaries from nursing facility and Home- and Community-Based Services waiver programs. *Journal of the American Geriatric Society* 2012;60(5):821-9. doi: 10.1111/j.1532-5415.2012.03920.x
12. Carter M, Porell F. Vulnerable populations at risk of potentially avoidable hospitalizations: the case of nursing home residents with Alzheimer's disease. *American Journal of Alzheimer's Disease and Other Dementias* 2005;20(6):349-58.
13. Krüger K, Jansen K, Grimsmo A. Hospital admissions from nursing homes: Rates and reasons. *Nursing Research and Practice* 2011 doi: 10.1155/2011/247623
14. Ouslander J, Diaz S, Hain D. Frequency and diagnoses associated with 7- and 30- day readmission of skilled nursing facility patients to a nonteaching community hospital. *Journal of the American Medical Directors Association* 2011;12(3):195-203. doi: 10.1016/j.jamda.2010.02.015
15. Ouslander J, Lamb G, Tappen R. Interventions to reduce hospitalizations from nursing homes: evaluation of the INTERACT II collaborative quality improvement project. *Journal of the American Geriatric Society* 2011;59(4):745-53. doi: 10.1111/j.1532-5415.2011.03333.x
16. Alessi C, Harker J. A prospective study of acute illness in the nursing home. *Aging* 1998;10(6):479-89.

For peer review only - http://bmjopen.bmj.com/site/about/guidelines.xhtml

17. Mor V, Intrator O, Fries B. Changes in hospitalization associated with introducing the Resident Assessment Instrument. *Journal of the American Geriatric Society* 1997;45(8):1002-10.
18. Young Y, Inamdar S, Dichter B. Clinical and nonclinical factors associated with potentially preventable hospitalizations among nursing home residents in New York State. *Journal of the American Medical Directors Association* 2011;12(5):364-71. doi: 10.1016/j.jamda.2010.03.006
19. Ouslander J, Perloe M, Givens J. Reducing potentially avoidable hospitalizations of nursing home residents: results of a pilot quality improvement project. *Journal of the American Medical Directors Association* 2009;10(9):644-52. doi: 10.1016/j.jamda.2009.07.001
20. Olsan T. Reducing avoidable Rehospitalisations by Improving Residents' Transitions to the Nursing Home. 2011:B22.
21. Sampson EL, Feast A, Blighe A, et al. Evidence-based intervention to reduce avoidable hospital admissions in care home residents (the Better Health in Residents in Care Homes (BHiRCH) study): protocol for a pilot cluster randomised trial. *BMJ open* 2019;9(5):e026510. doi: 10.1136/bmjopen-2018-026510 [published Online First: 2019/05/28]
22. Kitson AL, Rycroft-Malone J, Harvey G, et al. Evaluating the successful implementation of evidence into practice using the PARiHS framework: theoretical and practical challenges. *ImplementSci* 2008;3:1.
23. Schulz KF, Altman DG, Moher D, et al. CONSORT 2010 statement: updated guidelines for reporting parallel group randomised trials. *BMJ* 2010;340:c332. doi: 10.1136/bmj.c332 [published Online First: 2010/03/25]
24. Dewing J, McCormack B, Titchen A. Practice Development Workbook for Nursing, Health and Social Care Teams. West Sussex: John Wiley and Sons 2014.
25. Rycroft-Malone J, Harvey G, Seers K, et al. An exploration of the factors that influence the implementation of evidence into practice. *JClinNurs* 2004;13(8):913-24.
26. University. FA. Stop and Watch Early Warning Tool 2018 [Available from: http://www.pathway-interact.com/wp-content/uploads/2017/04/148604-Stop-and-Watch-v4_0.pdf accessed 20/02/2020.
27. Mahoney FI, Barthel DW. Functional Evaluation: The Barthel Index. *Md State Med J* 1965;14:61-5. [published Online First: 1965/02/01]
28. Edvardsson D, Fetherstonhaugh D, Nay R, et al. Development and initial testing of the Person-centered Care Assessment Tool (P-CAT). *International psychogeriatrics / IPA* 2010;22(1):101-8. doi: 10.1017/S1041610209990688 [published Online First: 2009/07/28]
29. Tjia J, Mazor KM, Field T, et al. Nurse-physician communication in the long-term care setting: perceived barriers and impact on patient safety. *J Patient Saf* 2009;5(3):145-52. doi: 10.1097/PTS.0b013e3181b53f9b [published Online First: 2009/11/21]
30. Saliba D, Kington R, Buchanan J, et al. Appropriateness of the decision to transfer nursing facility residents to the hospital. *J Am Geriatr Soc* 2000;48(2):154-63. [published Online First: 2000/02/22]
31. Beecham J, Knapp M. Costing psychiatric interventions. In: Thornicroft G, ed. Measuring Mental Helath Needs. London: Gaskell 2001:200-24.
32. Herdman M, Gudex C, Lloyd A, et al. Development and preliminary testing of the new five-level version of EQ-5D (EQ-5D-5L). *Qual Life Res* 2011;20(10):1727-36. doi: 10.1007/s11136-011-9903-x [published Online First: 2011/04/12]
33. Curtis L, Burns A. Unit Costs of Health and Social Care 2017 2017 [updated 2017. Available from: http://www.pssru.ac.uk/project-pages/unit-costs/unit-costs-2017/2018.
34. Joint Formulary Committee. British National Formulary. London: BMJ Group and Pharmaceutical Press 2018.
35. Department of Health. National Schedule of Reference Costs- Year 2016-2017- NHS trusts and NHS foundation trusts: NHS own cost. London, 2016.

36. Devlin NJ, Shah KK, Feng Y, et al. Valuing health-related quality of life: An EQ-5D-5L value set for England. *Health Econ* 2017 doi: 10.1002/hec.3564 [published Online First: 2017/08/24]
37. Devlin NJ, Shah K, Feng Y, et al. Valuing health-related quality of life: an EQ-5D-5L value set for England. Research Paper 16/01. London, 2016.
38. van Hout B, Janssen MF, Feng YS, et al. Interim scoring for the EQ-5D-5L: mapping the EQ-5D-5L to EQ-5D-3L value sets. *Value Health* 2012;15(5):708-15. doi: 10.1016/j.jval.2012.02.008 [published Online First: 2012/08/08]
39. National Institute for Clinical Excellence. Position statement on use of the EQ-5D-5L valuation set, 2017.
40. Briggs AH, Gray AM. Handling uncertainty when performing economic evaluation of healthcare interventions. *Health technology assessment* 1999;3(2):1-134. [published Online First: 1999/08/17]
41. Kane RL, Huckfeldt P, Tappen R, et al. Effects of an Intervention to Reduce Hospitalizations From Nursing Homes: A Randomized Implementation Trial of the INTERACT Program. *JAMA Intern Med* 2017;177(9):1257-64. doi: 10.1001/jamainternmed.2017.2657 [published Online First: 2017/07/04]
42. Gordon AL, Franklin M, Bradshaw L, et al. Health status of UK care home residents: a cohort study. *Age Ageing* 2014;43(1):97-103. doi: 10.1093/ageing/aft077
43. Saliba D, Kington R, Buchanan J, et al. Appropriateness of the decision to transfer nursing facility residents to the hospital. *Journal of the American Geriatrics Society* 2000;2000/02/22:154-63.
44. Van den Block L, Honinx E, Pivodic L, et al. Evaluation of a Palliative Care Program for Nursing Homes in 7 Countries: The PACE Cluster-Randomized Clinical Trial. *JAMA Intern Med* 2019:1-10. doi: 10.1001/jamainternmed.2019.5349 [published Online First: 2019/11/12]
45. Ballard C, Corbett A, Orrell M, et al. Impact of person-centred care training and person-centred activities on quality of life, agitation, and antipsychotic use in people with dementia living in nursing homes: A cluster-randomised controlled trial. *PLoS Med* 2018;15(2):e1002500. doi: 10.1371/journal.pmed.1002500 [published Online First: 2018/02/07]
46. Gordon AL, Goodman C, Davies SL, et al. Optimal healthcare delivery to care homes in the UK: a realist evaluation of what supports effective working to improve healthcare outcomes. *Age Ageing* 2018;47(4):595-603. doi: 10.1093/ageing/afx195 [published Online First: 2018/01/10]
47. Sherlaw-Johnson C, Crump H, Curry N, et al. Transforming health care in nursing homes: An evaluation of a dedicated primary care service in outer east London. London, 2018.
48. Morgan B, Hejdenberg J, Hinrichs-Krapels S, et al. Do feasibility studies contribute to, or avoid, waste in research? *PLoS One* 2018;13(4):e0195951. doi: 10.1371/journal.pone.0195951 [published Online First: 2018/04/24]

For peer review only - http://bmjopen.bmj.com/site/about/guidelines.xhtml

Figure title and legend

Figure 1 title: Resident recruitment flowchart

For peer review only

For peer review only - http://bmjopen.bmj.com/site/about/guidelines.xhtml

Figure 1. Resident recruitment flowchart

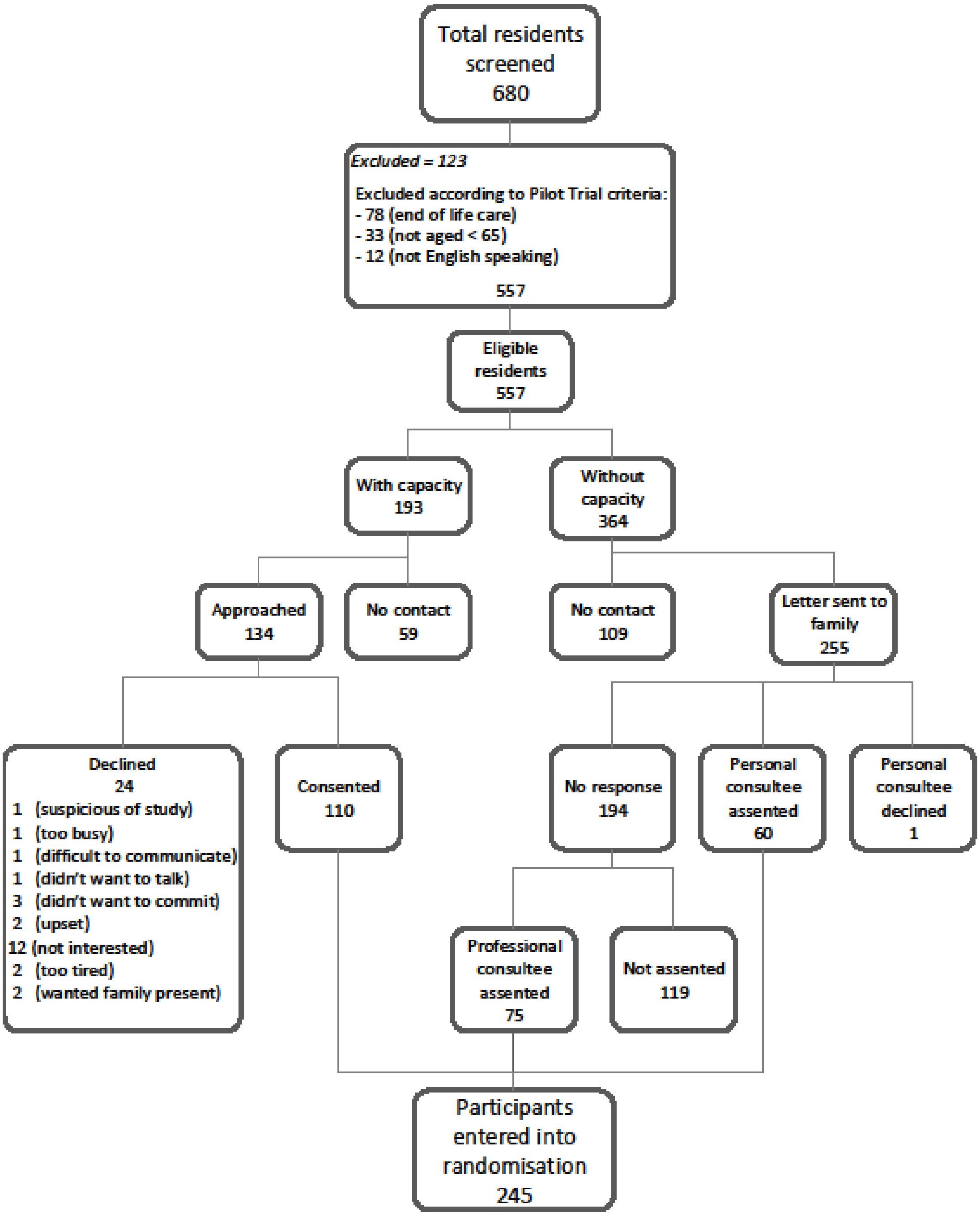

Better Health in Residents of Care Homes with Nursing - BHiRCH-NH study

Appendix 1: Pre-intervention characteristics of participating nursing homes

Characteristic	All care homes	TAU	BHiRCH-NH
	n/N or median (% or IQR)		
**Residents and beds**			
Beds available to new residents	1 (1,3)	2 (0, 2)	1 (1, 3)
Resident places	60 (36, 71)	67 (47, 84)	49 (28, 64)
Number of residents present in home	50 (34, 68)	50 (47, 83)	46 (25, 63)
Number of residents with dementia	20 (15, 33)	33 (15, 44)	20 (13, 23)
Number of residents in hospital	0 (0, 1)	1 (0, 2)	0 (0, 1)
**Medical attendances**			
Number hospital admissions	3 (2, 5)	4 (2, 7)	3 (2, 4)
Number of ambulances called	3 (2, 6)	4 (2, 9)	3 (2, 6)
Unscheduled (out of hours) GP visits or telephone contacts	1 (1, 3)	2 (1, 3)	1 (1, 3)
Accident and Emergency attendances	3 (2, 5)	3 (1, 4)	3 (2, 6)
**Staffing**			
Qualified nursing staff rostered on during the day	3 (2, 5)	3 (2, 5)	3 (2, 5)
Care staff rostered on during the day	11 (9, 13)	11 (9, 14)	12 (6, 13)
Qualified nursing staff rostered on during the night	2 (1, 3)	2 (1, 3)	2 (1, 3)
Care staff rostered on during the night	3 (3, 7)	3 (3, 7)	4 (2, 7)
Number of agency/ bank staff in 24 hour period	1 (0, 3)	1 (0, 3)	1 (0, 3)
Number of permanent registered nursing staff (including those on sick/carer/compassionate leave)	10 (8, 13)	11 (10, 13)	8 (7, 15)
Number of permanent other care staff (including those on sick/carer/compassionate leave)	26 (24, 57)	26 (24, 70)	33 (23, 57)
Number of registered nursing staff from those above on sick/carer/ compassionate leave	0 (0, 1)	0 (0, 1)	0 (0, 1)
Number of other care staff from those above on sick/carer/ compassionate leave	0 (0, 2)	1 (0, 6)	0 (0, 2)
**Nursing home**			
Privately managed	12/13 (92)	6/7 (86)	6/6 (100)
Local Authority managed	1/13 (8)	1/7 (14)	0/6 (0)
Nursing	6/13 (46)	4/7 (57)	2/6 (33)
Nursing and personal care	7/13 (54)	3/7 (43)	4/6 (67)
Dementia registered	8/11 (73)	5/6 (83)	3/5 (60)
Dementia specialist	2/12 (17)	2/6 (33)	0/6 (0)
**Regular nursing home attendance from**			
Physiotherapist	7/12* (58)	4/7 (57)	3/5 (60)
Geriatrician	3/12 (25)	2/7 (29)	1/5 (20)
District Nurse	5/13 (38)	3/7 (43)	2/6 (33)
Tissue viability nurses	7/12 (58)	3/7 (43)	4/5 (80)
Dietitian	9/13 (69)	4/7 (57)	5/6 (83)
Speech and language therapist	8/13 (62)	4/7 (57)	4/6 (67)
Optician	11/13 (85)	5/7 (71)	6/6 (100)
Ophthalmologist	4/13 (31)	1/7 (14)	3/6 (50)
Chiropodist	13/13 (100)	7/7 (100)	6/6 (100)
Occupational therapist	7/13 (54)	3/7 (43)	4/6 (67)
Dentist	8/12 (67)	5/7 (71)	3/5 (60)
Audiologist	2/12 (17)	1/7 (14)	1/5 (20)
Dedicated GP for all residents	13/13 (100)	7/7 (100)	6/6 (100)

***for some variables N=12 where this data was not available for a particular home**

For peer review only

Appendix 2. Family carer recruitment and retention

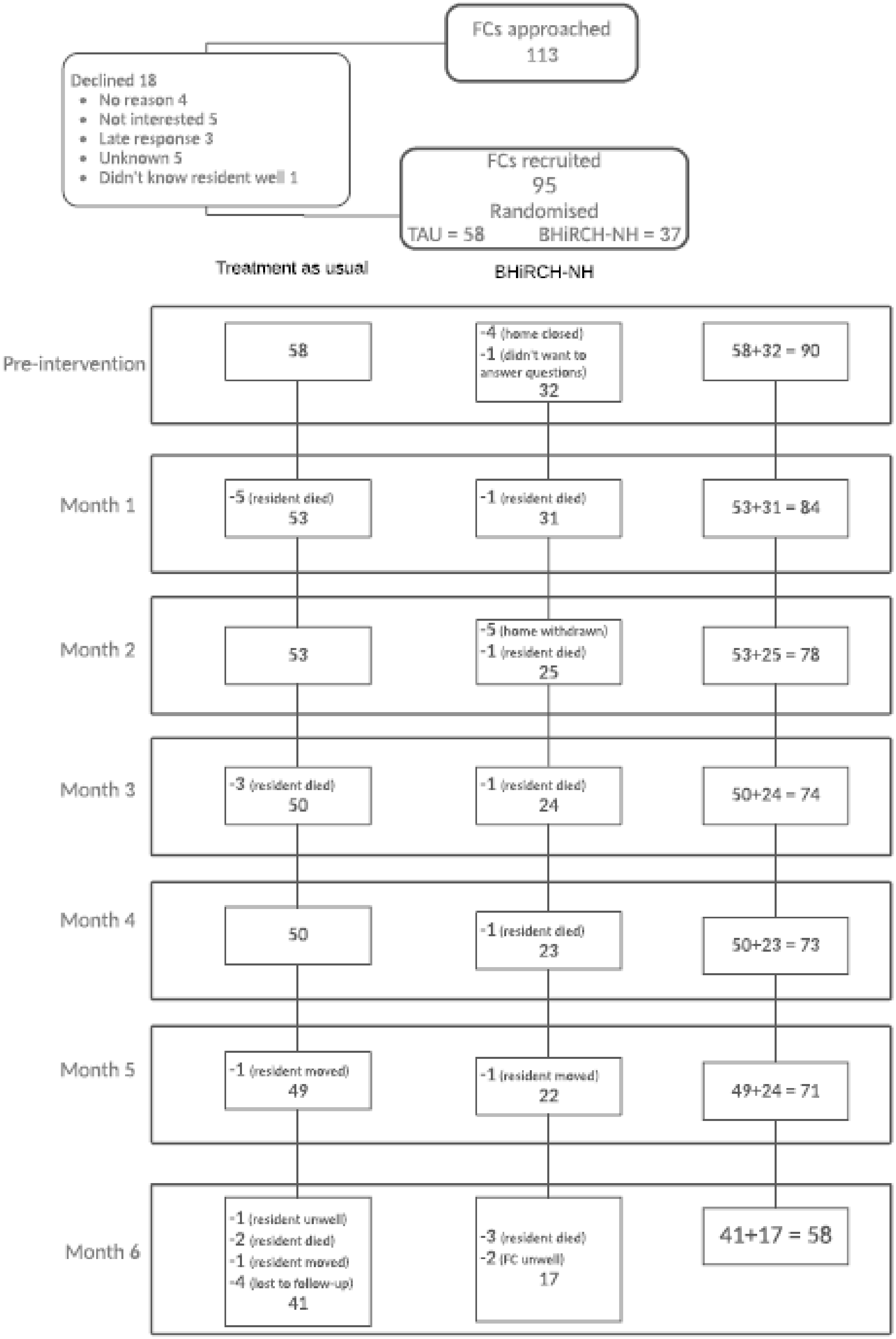

Appendix 3. Staff recruitment and retention flowchart

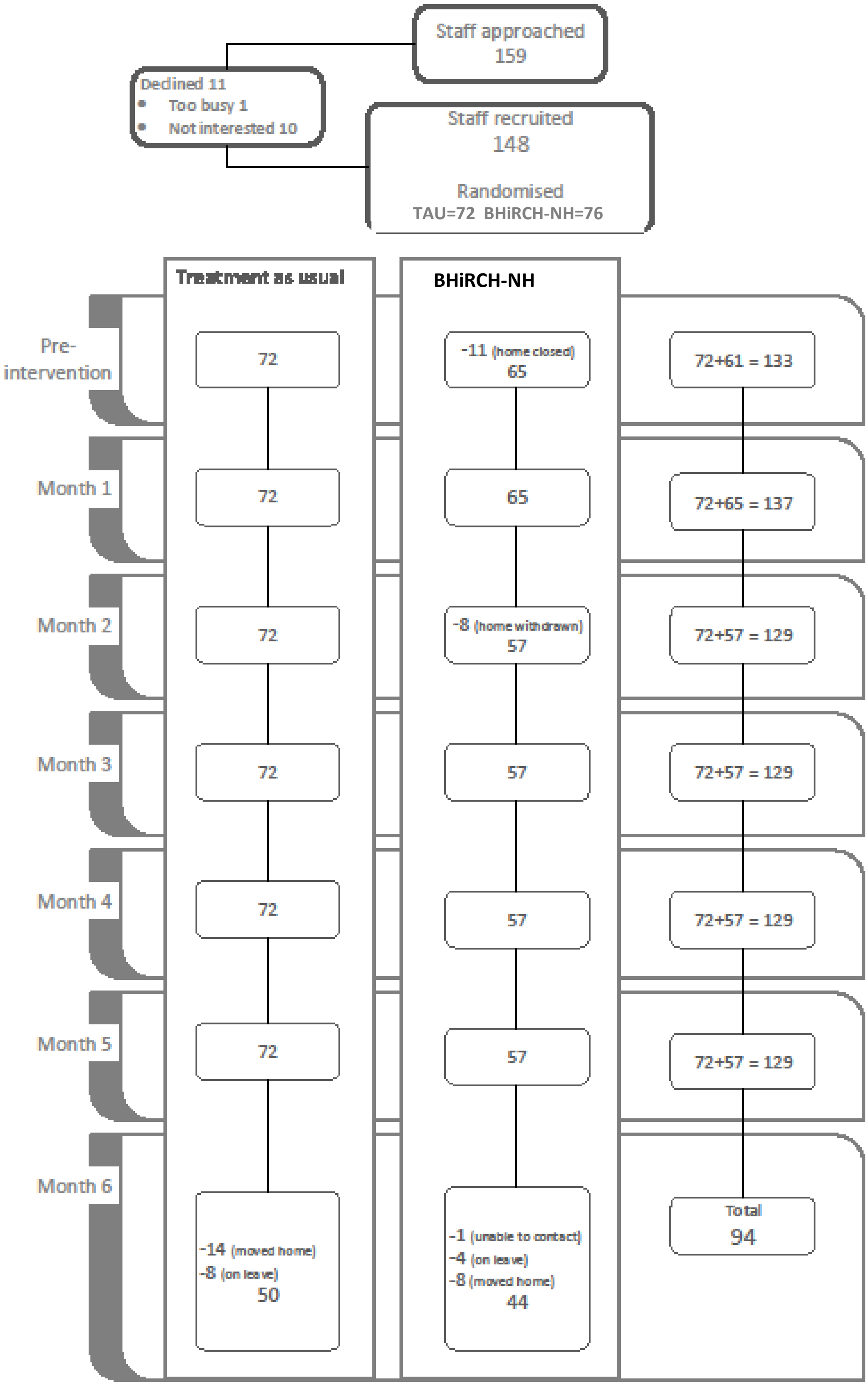

Better Health in Residents of Care Homes with Nursing - BHiRCH-NH study

Appendix 4: Staff participant characteristics pre-intervention

Characteristic	Cohort	TAU	Intervention
	n/N or median (% or IQR)		
**Demographics**	N=130	N=70	N=60
Male	16 (12)	9 (13)	7 (12)
Age	42 (30, 53)	42(32, 53)	39 (27, 50)
**Ethnicity**	N=129	N=69	N=60
White	76 (59)	32 (46)	44 (73)
Black	29 (22)	21 (30)	8 (13)
Asian	16 (12)	9 (13)	7 (12)
Other	8 (16)	7 (10)	1 (2)
**Marital status**	N=129	N=69	N=60
Married or cohabiting	71 (55)	40 (58)	31 (52)
Single	47 (36)	22 (32)	25 (42)
Divorced or widowed	11 (9)	7 (10)	4 (7)
**Education**	N=128	N=69	N=59
Completed years of education	11(11, 12)	12 (11, 12)	11 (11, 12)
No qualifications or GCSE or equivalent	16 (13)	9 (13)	7 (12)
A Level/NVQ/ HNC/ HND or equivalent	46 (36)	18 (26)	28 (47)
Degree or higher degree	49 (38)	28 (41)	21 (36)
Other qualification	17 (13)	14 (20)	3 (5)
**Type of staff**	N=125	N=67	N=58
Registered Nurse	24 (19)	9 (13)	7 (12)
Manager/ Matron/ Charge Nurse	12 (10)	8 (12)	4 (7)
Staff Nurse	2 (2)	5 (7)	3 (5)
Agency Nurse	87 (70)	1 (1)	1 (2)
Not a nurse		44 (66)	43 (74)
**Nurses only**	N=40	N=24	N=16
Works days	28 (70)	16 (67)	12 (75)
Works evenings	2 (5)	1 (4)	1 (6)
Works nights	9 (23)	4 (17)	5 (31)
Works mornings/early 12-hour shifts	12 (30)	5 (21)	7 (44)
Works afternoons/late 12-hour shifts	3 (8)	0 (0)	3 (19)
Works weekends	3 (8)	1 (4)	2 (13)
**Number of hours/week working in this nursing home**	N=40	N=24	N=16
Less than 8	1 (3)	0 (0)	1 (6)
8 to 12	0 (0)	0 (0)	0 (0)
13 to 40	13 (33)	8 (33)	5 (31)
More than 40	26 (65)	16 (67)	10 (63)
**How long have you worked at this nursing home?**	N=40	N=24	N=16
Less than 2 months	1 (3)	1 (4)	0
2 to 6 months	4 (10)	3 (13)	1 (6)
7 to 11 months	7 (18)	4 (17)	3 (19)
1 to 5 years	17 (43)	7 (29)	10 (63)
6 to 10 years	6 (15)	6 (25)	0
More than 10 years	5 (13)	3 (13)	2 (13)
**How long have you worked in nursing homes?**	N=39	N=24	N=15
7 to 11 months	2 (5)	1 (4)	1 (7)
1 to 5 years	14 (36)	6 (25)	8 (53)
6 to 10 years	7 (18)	7 (29)	0
More than 10 years	16 (41)	10 (42)	6 (40)
**How long have you been a registered nurse?**	N=40	N=24	N=16
1 to 5 years	8 (20)	3 (13)	5 (31)
6 to 10 years	10 (25)	7 (29)	3 (19)
More than 10 years	22 (55)	14 (58)	8 (50)

For peer review only

Better Health in Residents of Care Homes with Nursing - BHiRCH-NH study

**English is first language**	20 (50)	12 (50)	8 (50)
**PCAT Score**	49 (I46, 53)	50 (47,54)	48 (44,51)

For peer review only

Appendix 5: Nurse – General Practitioner Communication Tool scores

	Pre-intervention			6 month follow up	
	Cohort	TAU	BHiRCH-NH n/N (%)	TAU	BHiRCH-NH
Have difficulty understanding what GP means due to medical jargon	2/40 (5)	2/24 (8)	0/16 (0)	0/14 (0)	0/7 (0)
A GP's language or accent makes it hard for you to understand what they are saying	2/40 (5)	1/24 (4)	1/16 (16)	0/15 (0)	0/7 (0)
A GP has difficulty understanding what you are saying due to your language or accent	2/40 (5)	2/24 (8)	0/16 (0)	0/15 (0)	0/7 (0)
GPs interrupt you before you have finished reporting on a resident	4/40 (10)	2/24 (8)	2/16 (13)	1/14 (7)	0/7 0)
GPs consider nurses' views when making decisions about residents	29/39 (10)	18/23 (78)	11/16 (69)	12/15 (80)	5/7 (71)
GPs are rude to you when you call them about a resident	2/39 (5)	2/23 (9)	0/16 (0)	1/15 (7)	0/7 (0)
You feel respected after an interaction with a GP?	29/40 (73)	19/24 (79)	10/16 (63)	12/15 (80)	4/7 (57)
You feel frustrated after an interaction with a GP?	4/40 (10)	3/24 (13)	1/16 (6)	1/15 (7)	0/7 (0)
Difficulty reaching the GP	9/40 (23)	5/24 (21)	4/16 (25)	2/15 (13)	2/7 (29)
Uncertainty about what to tell the GP	1/38 (3)	1/23 (4)	0/15 (0)	0/15 (0)	0/7 (0)
Feeling that the GP doesn't want to deal with the problem	3/40 (8)	1/24 (4)	2/16 (13)	1/15 (7)	1/7 (14)
Finding time to make the call	11/40 (28)	8/24 (33)	3/16 (19)	3/15 (20)	2/7 (29)
Finding a quiet place to make the call	10/40 (25)	7/24 (29)	3/16 (19)	4/14 (29)	0/7 (0)
Anticipating that the GP will be rude or unpleasant	1/40 (3)	0/24 (0)	1/16 (6)	1/15 (7)	0/7 (0)
Feeling hurried by the GP	7/40 (18)	4/24 (17)	3/16 (19)	1/15 (7)	0/7 (0)
Feeling that I am bothering the GP	4/39 (10)	2/24 (8)	2/15 (13)	2/15 (13)	0/7 (0)
Worrying that the GP may order something inappropriate or unnecessary	4/40 (10)	2/24 (8)	2/16 (13)	1/15 (7)	0/7 (0)
Feeling that I don't have enough time to say everything that I need to say	3/39 (8)	1/24 (4)	2/15 (13)	1/15 (7)	0/7 (0)
Nurses get good training in this care home	28/39 (72)	16/24 (67)	12/15 (80)	14/15 (93)	5/7 (71)
Nurses in this care home are willing to try new protocols	33/39 (85)	20/24 (83)	13/15 (87)	11/15 (73)	5/7 (71)
Nurses in this care home sometimes have to ignore protocols to get everything done	6/39 (15)	4/24 (17)	2/15 (13)	3/15 (20)	1/7 (14)
When this care home makes changes to improve resident care, they follow up to see if the changes worked	31/39 (79)	18/24 (75)	13/15 (87)	15/15 (100)	6/7 (86)
It is hard to make changes to improve resident care in this care home	4/39 (10)	1/24 (4)	3/15 (20)	2/15 (13)	0/7 (0)
This care home often wants nurses to follow protocols that don't really help residents	3/39 (8)	1/24 (4)	2/15 (13)	2/15 (13)	0/7 (0)
New protocols often make it harder for nurses to do their job	4/39 (10)	2/24 (8)	2/15 (13)	4/15 (27)	0/7 (0)
The communication between nurses and GPs in this care home is open	34/40 (85)	20/24 (83)	14/16 (88)	13/15 (87)	7/7 (100)
Feel comfortable communicating with GPs?	36/40 (90)	23/24 (96)	13/16 (81)	15/15 (100)	7/7 (100)
Feel comfortable communicating with nurse practitioners?	34/40 (85)	23/24 (96)	11/16 (69)	14/15 (93)	6/6 (100)

For peer review only

Better Health in Residents of Care Homes with Nursing - BHiRCH-NH study

Appendix 6: Nurse self- assessed core competencies

Core competencies	Pre-intervention Cohort	TAU	BHiRCH-NH	6 month follow up TAU	BHiRCH-NH
			n/N (%)		
**I know how acute episodes of chronic heart failure may manifest in older people**					
Disagree	0/34 (0)	0/21 (0)	0/13 (0)	0/15 (0)	0/7 (0)
Neither agree nor disagree	2/34 (6)	0/21 (0)	2/13 (15)	1/15 (7)	0/7 (0)
Agree	18/34 (53)	12/21 (57)	6/13 (46)	7/15 (47)	4/7 (57)
Completely agree	14/34 (41)	9/21 (43)	5/13 (38)	7/15 (47)	3/7 (43)
**I know how respiratory infections may manifest in older people**					
Disagree	0/34 (0)	0/21 (0)	0/13 (0)	0/15 (0)	0/7 (0)
Neither agree nor disagree	0/34 (0)	0/21 (0)	0/13 (0)	0/15 (0)	0/7 (0)
Agree	13/34 (38)	7/21 (33)	6/13 (46)	6/15 (40)	3/7 (43)
Completely agree	21/34 (62)	14/21 (67)	7/13 (54)	9/15 (60)	4/7 (57)
**I know how dehydration may manifest in older people**					
Disagree	0/34 (0)	0/21(0)	0/13 (0)	0/15 (0)	0/7 (0)
Neither agree nor disagree	0/34 (0)	0/21 (0)	0/13 (0)	0/15(0)	0/7 V
Agree	14/34 (41)	7/21 (33)	7/13 (54)	5/15 (33)	1/7 (14)
Completely agree	20/34 (59)	14/21 (67)	6/13 (46)	10/15 (67)	6/7 (86)
**I know how urinary tract infections may manifest in older people**					
Disagree	0/34 (0)	0/21 (0)	0/13 (0)	0/15 (0)	0/7 (0)
Neither agree nor disagree	0/34 (0)	0/21 (0)	0/13 (0)	0/15 (0)	0/7 (0)
Agree	15/3 4 (44)	9/21 (43)	6/13 (46)	5/15	1/7 (14)
Completely agree	19/34 (56)	12/21 (57)	7/13 (54)	10/15 (67)	6/7 (86)
**I am able to identify changes in physiology or behaviour as compared to baseline or normal for that resident**					
Disagree	0/34 (0)	0/21 (0)	0/13 (0)	0/15 (0)	0/7 (0)
Neither agree nor disagree	0/34 (0)	0/21 (0)	0/13 (0)	0/15 (0)	0/7 (0)
Agree	14/34 (41)	8/21(38)	6/13 (46)	5/15 (33)	0/7 (0)
Completely agree	20/34 (59)	13/21 (62)	7/13 (54)	10/15 (67)	7/7 (100)
**I am able to identify changes in physiology or behaviour for people who cannot communicate verbally**					
Disagree	0/34 (0)	0/21	0/13 (0)	0/15 (0)	0/7 (0)
Neither agree nor disagree	1/34 (3)	0/21(0)	1/13 (8)	1/15 (7)	0/7 (0)
Agree	17/34 (50)	11/21 (52)	6/13 (46)	6/15 (40)	2/7 (29)
Completely agree	16/34 (47)	10/21 (48)	6/13 (46)	8/15 (53)	5/7 (71)
**I know each resident's existing medical conditions**					
Disagree	1/34 (3)	0/21 (0)	1/13 (8)	0/15 (0)	1/7 (14)
Neither agree nor disagree	2/34 (6)	1/21 (5)	1/13 (8)	1/15 (7)	0/7 (0)
Agree	22/34 (65)	15/21 (71)	7/13 (54)	8/15 (53)	6/7 (86)
Completely agree	9/34 (26)	5/21 (24)	4/13 (31)	6/15 (40)	0/7 (0)
**I know each resident's care plans with respect to these medical conditions**					
Disagree	1/34 (3)	0/21 (0)	1/13 (8)	0/15 (0)	0/7(0)
Neither agree nor disagree	2/34 (6)	1/21 (5)	1/13 (8)	1/15 (7)	1/7 (14)
Agree	17/34 (50	10/21 (48)	7/13 (54)	8/15 (53)	5/7 (71)

Completely agree	14/34 (41)	10/21 (48)	4/13 (31)	6/15 (40)	1/7 (14)
**I know what is normal (i.e. their baseline) for each resident in terms of physiology, abilities and behaviour**					
Disagree	0/34 (0)	0/21 (0)	0/13 (0)	0/15 (0)	0/7 (0)
Neither agree nor disagree	1/34 (3)	1/21 (5)	0/13 (0)	2/15 (13)	0/7(0)
Agree	19/34 (56)	11/21 (52)	8/13 (62)	7/15 (47)	6/7 (86)
Completely agree	14/34 (41)	9/21 (43)	5/13 (38)	6/15 (40)	1/7 (14)
**I am able to carry out immediate interventions (eg adjusting fluid levels)**					
Disagree	1/33 (3)	1/21 (5)	0/12 (0)	0/15 (0)	0/7 (0)
Neither agree nor disagree	0/33 (0)	0/21 (0)	0/12 (0)	0/15 (0)	1/7 (14)
Agree	21/33 (64)	10/21 (48)	11/12 (92)	6/15 (40)	6/7(86)
Completely agree	11/33 (33)	10/21 (48)	1/12 (8)	9/15 (60)	0/7 (0)
**I am able to recognise verbal and nonverbal signs of acute deterioration**					
Disagree	0/34 (0)	0/21 (0)	0/13 (0)	0/15 (0)	0/7 (0)
Neither agree nor disagree	0/34 (0)	0/21 (0)	0/13 (0)	1/15 (7)	0/7 (0)
Agree	19/34 (56)	10/21 (48)	9/13 (69)	6/15 (40)	4/7 (57)
Completely agree	15/34 (44)	11/21 (52)	4/13 (31)	8/15 (53)	3/7 (43)
**I am able to carry out bedside observations (vital signs etc)**					
Disagree	0/34 (0)	0/21 (0)	0/13 (0)	0/15 (0)	0/7 (0)
Neither agree nor disagree	0/34 (0)	0/21 (0)	0/13 (0)	0/15 (0)	0/7 (0)
Agree	11/34 (32)	6/21 (29)	5/13 (38)	5/15 (33)	4/7 (57)
Completely agree	23/34 (68)	15/21 (71)	8/13 (62)	10/15(67)	3/7 (43)
**I am able to utilise basic clinical skills (use of medication etc)**					
Disagree	0/34 (0)	0/21 (0)	0/13 (0)	0/15 (0)	0/7 (0)
Neither agree nor disagree	0/34 (0)	0/21 (0)	0/13 (0)	0/15 (0)	0/7 (0)
Agree	11/34 (32)	5/21 (24)	6/13	5/15	3/7
Completely agree	23/34 (68)	16/21 (76)	7/13 (54)	10/15 (67)	4/7 (57)
I **know when to seek additional support (eg. asking a nurse, calling a GP or ambulance)**					
Disagree	0/34 (0)	0/21 (0)	0/13 (0)	0/15 (0)	0/7 (0)
Neither agree nor disagree	0/34 (0)	0/21 (0)	0/13 (0)	0/15 (0)	0/7 (0)
Agree	10/34 (29)	4/21 (19)	6/13 (46)	4/15 (27)	2/7 (29)
Completely agree	24/34 (71)	17/21 (81)	7/13 (54)	11/15 (73)	5/7 (71)
**I am able to set and monitor staff work to ensure quality of health care is provided**					
Disagree	1/34 (3)	1/21 (5)	0/13 (0)	1/15 (7)	0/7(0)
Neither agree nor disagree	0/34 (0)	0/21 V	0/13 V	0/15 (0)	0/7 (0)
Agree	14/34 (41)	5/21 (24)	9/13 (69)	4/15 (27)	3/7 (43)
Completely agree	19/34 (56)	15/21 (71)	4/13 (31)	10/15 (67)	4/7 (57)
**I am able to encourage or support other staff in their care for acute deterioration in resident's health**					
Disagree	0/34 (0)	0/21 V	0/13 (0)	0/15 (0)	0/7 (0)
Neither agree nor disagree	2/34 (6)	2/21 (10)	0/13 (0)	0/15 (0)	0/7 (0)
Agree	14/34 (41)	5/21 (24)	9/13 (69)	6/15 (40)	3/ (43)7
Completely agree	18/34 (53)	14/21 (67)	4/13 (31)	9/15 (60)	4/7 (57)
**I am able to negotiate and plan a course of action with other staff**					
Disagree	0/34 (0)	0/21 (0)	0/13 (0)	0/15 (0)	0/7 (0)
Neither agree nor disagree	4/34 (12)	3/21 (14)	1/13 (8)	1/15 (7)	0/7 (0)
Agree	13/34 (38)	6/21 (29)	7/13 (54)	5/15 (33)	5/7 (71)

For peer review only

Completely agree	17/34 (50)	12/21 (57)	5/13 (38)	9/15 (60)	2/7 (29)
**I am able to record a plan of action, e.g. draft and update care plans**					
Disagree	0/34 (0)	0/21 (0)	0/13 (0)	0/15 (0)	0/ (0)7
Neither agree nor disagree	0/34 (0)	0/21 (0)	0/13 (0)	1/15 (7)	0/7 (0)
Agree	16/34 (47)	10/21 (48)	6/13 (46)	4/15 (27)	3/7 (43)
Completely agree	18/34 (53)	11/21 (52)	7/13 (54)	10/15 (67)	4/7 (57)
**I am able to ensure acute changes in a resident's health status are communicated to staff in written or oral form**					
Disagree	0/34 (0)	0/21 (0)	0/13 (0)	0/15 (0)	0/7 (0)
Neither agree nor disagree	0/34 (0)	0/21 (0)	0/13 (0)	1/15 (7)	0/7(0)
Agree	14/34 (41)	7/21 (33)	7/13 (54)	5/15 (33)	3/7 (43)
Completely agree	20/34 (59)	14/21 (67)	6/13 (46)	9/15 (60)	4/7 (57)
**I am able to ensure effectiveness of treatment for acute changes in a resident's health status are communicated to staff and carers in written or oral form**					
Disagree	0/34 (0)	0/21 (0)	0/13 (0)	0/15 (0)	0/7 (0)
Neither agree nor disagree	2/34 (6)	1/21 (5)	1/13 (8)	1/15 (7)	0/7 (0)
Agree	12/34 (35)	6/21 (29)	6/13 (46)	6/15 (40)	2/7 (29)
Completely agree	20/34 (59)	14/21 (67)	6/13 (46)	8/15 (53)	5/7 (71)
**I am able to convey information about changes in health accurately and sensitively to carers**					
Disagree	0/34 (0)	0/21 (0)	0/13 (0)	0/15 (0)	0/6 (0)
Neither agree nor disagree	1/34 (3)	0/21 (0)	1/13 (8)	1/15 (7)	0/6 (0)
Agree	16/34 (47)	10/21 (48)	6/13 (46)	6/15 (40)	2/6 (33)
Completely agree	17/34 (50)	11/21 (52)	6/13 (46)	8/15 (53)	4/6 (67)
**I am able to adapt assessment and communication for people with dementia or communication difficulties**					
Disagree	1/34 (3)	1/21 (5)	0/13 (0)	0/15 (0)	0/7 (0)
Neither agree nor disagree	1/34 (3)	1/21 (5)	0/13 (0)	1/15 (7)	0/7 (0)
Agree	17/34 (50)	11/21 (52)	6/13 (460	6/15 (40)	3/7 (43)
Completely agree	15/34 (44)	8/21 (38)	7/13 (54)	8/15 (53)	4/7 (57)
**I am able to communicate the nature of change in a resident's health status**					
Disagree	0/34 (0)	0/21 (0)	0/13 (0)	0/15 (0)	0/7 (0)
Neither agree nor disagree	0/34 (0)	0/21 (0)	0/13 (0)	1/15 (7)	0/7 (0)
Agree	17/34 (50)	12/21 (57)	5/13 (38)	5/15 (33)	3/7 (43)
Completely agree	17/34 (50)	9/21 (43)	8/13 (62)	9/15 (60)	4/7 (57)

For peer review only

Better Health in Residents of Care Homes with Nursing - BHiRCH-NH study

Appendix 7. Serious adverse events by randomised group

SAE	TAU N=55* n/N	Intervention N=41* n/N
**Death**	17 (31)	14 (34)
**Requires hospitalisation or prolonged hospital stay**	37 (67)	25 (61)
**Other medically significant**	1 (2)	2 (5)
**Not caused by the intervention**	55 (100)	41 (100)
**Expected side effect or outcome of the intervention**	55 (100)	39 (95)
**Not expected**	0 (0)	2 (5)
**Not related to the intervention**	55 (100)	41 (100)

*(denominator is SAE not residents)

For peer review only

Appendix 8. Resident retention flowchart

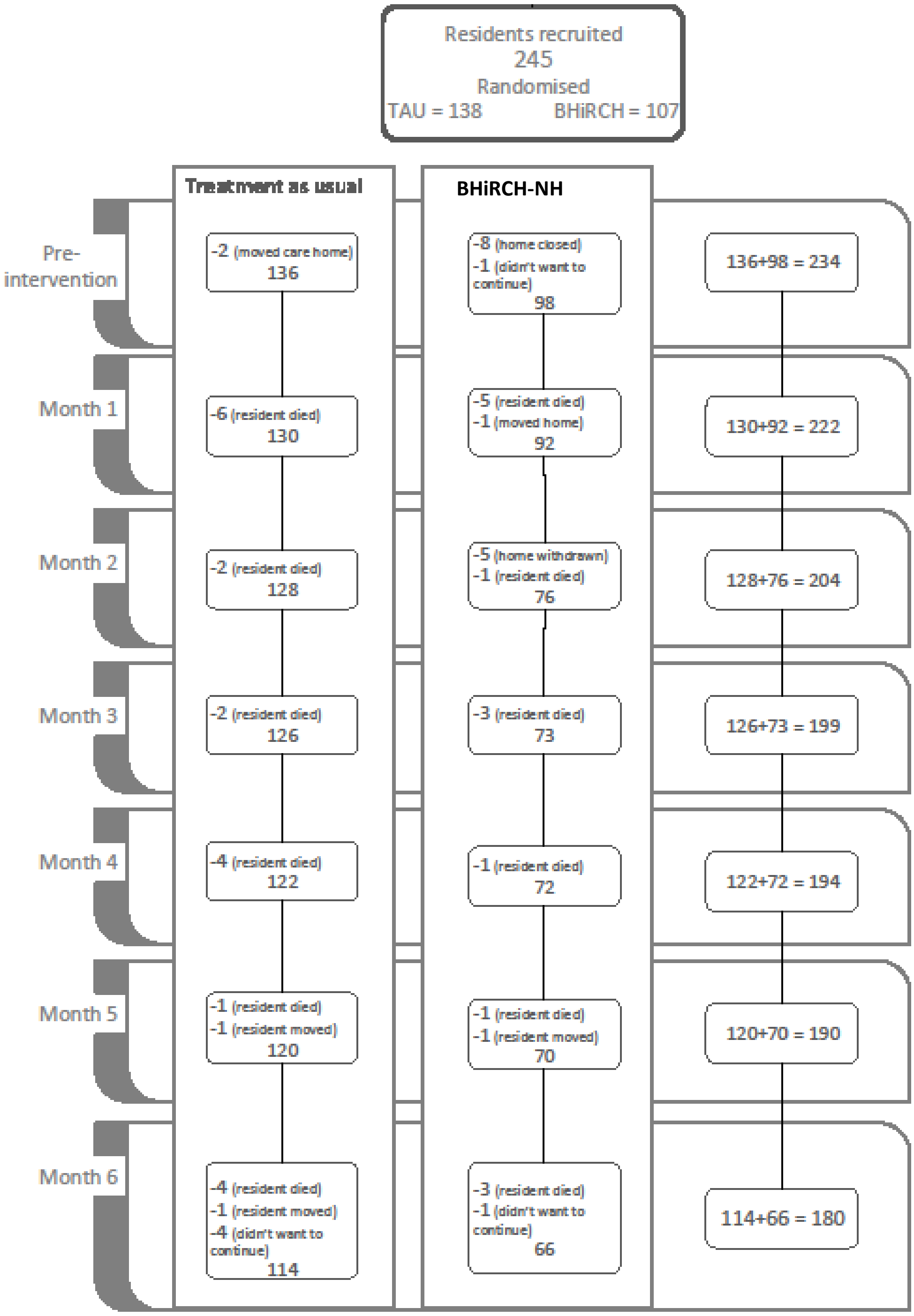

Appendix 9. Mean healthcare resource use ± standard deviation (SD) and cost (unit costs at 2016/2017 values) per resident over the 6 months, complete data

Type of resource use (unit)	TAU	BHiRCH-NH	Difference (BHiRCH-NH vs TAU)		TAU	BHiRCH-NH	Incremental difference (BHiRCH-NH vs TAU)
	Mean ± SD	Mean ± SD	Mean	p-value	Mean cost	Mean cost	Mean cost (95% CI)
**Primary care, community health or emergency services (visits)**	8.3 ± 6.1 *(n=114)*	9.6 ± 7.6 *(n=71)*	1.3	0.20	£491.3	£694.9	£203.6 (-£67.4 to £474.6)
**Outpatient appointments (visits)**	0.4 ± 1.0 *(n=114)*	0.7 ± 1.5 *(n=68)*	0.3	0.11	£22.6	£49.9	£27.4 (£1.2 to £53.5)
**Inpatient services (admissions)**	0.2 ± 0.5 *(n=114)*	0.2 ± 0.6 *(n=68)*	0.001	0.99	£356.9	£426.6	£69.6 (-£333.8 to £472.9)
**Ambulance service (calls)**	0.2 ± 0.6 *(n=114)*	0.2 ± 0.5 *(n=68)*	0.04	0.61	£1.3	£1.6	£0.3 (-£0.9 to £1.4)
**Accident and Emergency (attendances)**	0.1 ± 0.5 *(n=114)*	0.3 ± 0.6 *(n=68)*	0.1	0.21	£23.1	£38.7	£15.6 (-£8.9 to £40.1)
**Prescriptions (packages)**	54.1 ± 23.8 *(n=114)*	60.5 ± 26.6 *(n=68)*	6.33	0.10	£325.7	£381.6	£55.9 (-£43.8 to £155.6)
**Length of stay in hospital (days)**	0.8 ± 3.9 *(n=114)*	0.9 ± 3.9 *(n=68)*	0.2	0.76			

For peer review only

Better Health in Residents of Care Homes with Nursing - BHiRCH-NH study

Appendix 10. Mean utility values and QALYs per resident (based on resident self-completed EQ-5D-5L questionnaire)

Utility values	TAU		BHiRCH-NH		Incremental difference	
	Mean	(95%CI)	Mean	(95%CI)	Mean	p-value
**Complete cases**						
Baseline	0.610 *(n=69)*	0.540 to 0.680	0.487 *(n=58)*	0.403 to 0.570	-0.123	0.02
6 months	0.685 *(n=59)*	0.610 to 0.759	0.614 *(n=32)*	0.501 to 0.727	-0.071	0.28
**QALYs**	0.337 *(n=46)*	0.297 to 0.377	0.259 *(n=26)*	0.196 to 0.321	-0.078	0.03
**With imputations**						
Baseline	0.617	0.607 to 0.628	0.504	0.491 to 0.518	-0.113	<0.001
6 months	0.652	0.641 to 0.662	0.649	0.637 to 0.661	-0.003	0.73
**QALYs**	0.317	0.312 to 0.322	0.289	0.283 to 0.294	-0.029	<0.001

For peer review only

Better Health in Residents of Care Homes with Nursing - BHiRCH-NH study

Appendix 11. Mean cost ± standard deviation (SD) of BHiRCH-NH and TAU of resource use per resident over 6 months

Type of costs	TAU	BHiRCH-NH	BHiRCH-NH vs. TAU
**BHiRCH-NH intervention costs per resident**			
Training	£0 ± £0	£39 ± £28	£39
Materials used in training	£0 ± £0	£0.20 ± £0.10	£0.20
Delivery of the intervention	£0 ± £0	£34 ± £32	£34
**Total costs (95%CI) per resident of healthcare resource use over 6 months**			
Complete cases	£1,250 (£900 to £1,599) *(n=105)*	£1,560 (£912 to £2,209) *(n=66)*	£310 (-£361 to £981) *(n=171)*
With imputation	£1,233 (£1,171 to £1,295)	£1,458 (£1,351 to £1,566)	£225 (£108 to £342)
**Total costs (95%CI) per resident of BHiRCH-NH intervention cost + healthcare resource use over 6 months**			
Complete cases	£1,250 (£900 to £1,599) *(n=105)*	£1,640 (£989 to £2,291) *(n=66)*	£390 (-£283 to £1,063) *(n=171)*
With imputation	£1,233 (£1,171 to £1,295)	£1,532 (£1,424 to £1,640)	£299 (£182 to £417)

For peer review only

Appendix 12. Cost-effectiveness acceptability curve showing the probability that BHiRCH-NH intervention is cost-effective versus TAU at different values of the WTP for a QALY, N=237

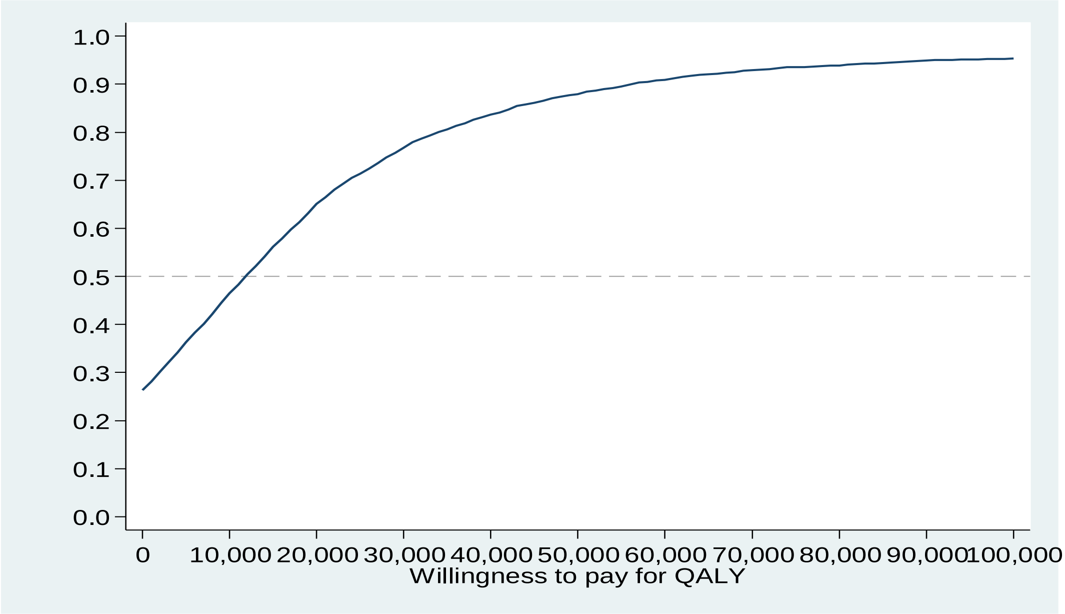

Figure 5 legend: Abbreviations: BHiRCH-NH-Better Health in Residents of Care Homes with Nursing study, QALY- Quality Adjusted Life year, TAU-Treatment as usual, WTP-Willingness to Pay

review only

For peer review only - http://bmjopen.bmj.com/site/about/guidelines.xhtml

For peer review only

For peer review only - http://bmjopen.bmj.com/site/about/guidelines.xhtml

CONSORT 2010 checklist of information to include when reporting a randomised trial*

Section/Topic	Item No	Checklist item	Reported on page No
**Title and abstract**			
	1a	Identification as a randomised trial in the title	Title page
	1b	Structured summary of trial design, methods, results, and conclusions (for specific guidance see CONSORT for abstracts)	Page 1
**Introduction**			
Background and objectives	2a	Scientific background and explanation of rationale	Page 4-5
	2b	Specific objectives or hypotheses	Page 5
**Methods**			
Trial design	3a	Description of trial design (such as parallel, factorial) including allocation ratio	Page 5
	3b	Important changes to methods after trial commencement (such as eligibility criteria), with reasons	Page 8
Participants	4a	Eligibility criteria for participants	Pages 6-7
	4b	Settings and locations where the data were collected	Pages 6-7
Interventions	5	The interventions for each group with sufficient details to allow replication, including how and when they were actually administered	Pages 8-9
Outcomes	6a	Completely defined pre-specified primary and secondary outcome measures, including how and when they were assessed	Pages 12-13
	6b	Any changes to trial outcomes after the trial commenced, with reasons	Page 8
Sample size	7a	How sample size was determined	Page 14
	7b	When applicable, explanation of any interim analyses and stopping guidelines	N/A
Randomisation:			
Sequence generation	8a	Method used to generate the random allocation sequence	Page 14
	8b	Type of randomisation; details of any restriction (such as blocking and block size)	Page 14
Allocation concealment mechanism	9	Mechanism used to implement the random allocation sequence (such as sequentially numbered containers), describing any steps taken to conceal the sequence until interventions were assigned	Page 14
Implementation	10	Who generated the random allocation sequence, who enrolled participants, and who assigned participants to interventions	Page 14
Blinding	11a	If done, who was blinded after assignment to interventions (for example, participants, care providers, those	Page 14

For peer review only - http://bmjopen.bmj.com/site/about/guidelines.xhtml

		assessing outcomes) and how	
	11b	If relevant, description of the similarity of interventions	N/A
Statistical methods	12a	Statistical methods used to compare groups for primary and secondary outcomes	Page 14-15
	12b	Methods for additional analyses, such as subgroup analyses and adjusted analyses	Page 15
**Results**			
Participant flow (a diagram is strongly recommended)	13a	For each group, the numbers of participants who were randomly assigned, received intended treatment, and were analysed for the primary outcome	Figure 1 Appendices 2,3,8
	13b	For each group, losses and exclusions after randomisation, together with reasons	Figures 1 Appendices 2,3,8
Recruitment	14a	Dates defining the periods of recruitment and follow-up	Page 7
	14b	Why the trial ended or was stopped	Page 7
Baseline data	15	A table showing baseline demographic and clinical characteristics for each group	Table 1
Numbers analysed	16	For each group, number of participants (denominator) included in each analysis and whether the analysis was by original assigned groups	Table 1
Outcomes and estimation	17a	For each primary and secondary outcome, results for each group, and the estimated effect size and its precision (such as 95% confidence interval)	Tables 1-4
	17b	For binary outcomes, presentation of both absolute and relative effect sizes is recommended	N/A
Ancillary analyses	18	Results of any other analyses performed, including subgroup analyses and adjusted analyses, distinguishing pre-specified from exploratory	N/A
Harms	19	All important harms or unintended effects in each group (for specific guidance see CONSORT for harms)	Appendix 7
**Discussion**			
Limitations	20	Trial limitations, addressing sources of potential bias, imprecision, and, if relevant, multiplicity of analyses	Page 24
Generalisability	21	Generalisability (external validity, applicability) of the trial findings	Pages 24
Interpretation	22	Interpretation consistent with results, balancing benefits and harms, and considering other relevant evidence	Pages 23-26
**Other information**			
Registration	23	Registration number and name of trial registry	Page 3
Protocol	24	Where the full trial protocol can be accessed, if available	Page 3
Funding	25	Sources of funding and other support (such as supply of drugs), role of funders	Page 26

For peer review only

For peer review only - http://bmjopen.bmj.com/site/about/guidelines.xhtml

*We strongly recommend reading this statement in conjunction with the CONSORT 2010 Explanation and Elaboration for important clarifications on all the items. If relevant, we also recommend reading CONSORT extensions for cluster randomised trials, non-inferiority and equivalence trials, non-pharmacological treatments, herbal interventions, and pragmatic trials. Additional extensions are forthcoming: for those and for up to date references relevant to this checklist, see www.consort-statement.org.

For peer review only

BMJ Open

A pilot cluster randomised trial of an evidence-based intervention to reduce avoidable hospital admissions in nursing home residents (Better Health in Residents of Care Homes with Nursing - BHiRCH-NH study)

Journal:	*BMJ Open*
Manuscript ID	bmjopen-2020-040732.R1
Article Type:	Original research
Date Submitted by the Author:	07-Aug-2020

Complete List of Authors:	Sampson, Elizabeth; UCL, Division of Psychiatry<br>Feast, Alexandra; UCL, Division of Psychiatry<br>Blighe, Alan; University of Bradford Faculty of Health Studies, Ccentre for Applied Dementia Studies<br>Froggatt, Katherine ; Lancaster University Faculty of Health and Medicine, Faculty of Health and Medicine; Lancaster University<br>Hunter, Rachael; UCL, Research Dept of Primary Care and Population Health<br>Marston, Louise; UCL<br>McCormack, Brendan; Queen Margaret University School of Health Sciences, Nursing; Queen Margaret University School of Health Sciences,<br><br>Nurock, Shirley; UCL, Division of Psychiatry<br>Panca, Monica; University College London, Primary Care and Population Health<br>Powell, Catherine; University of Bradford Faculty of Health Studies, Centre for Applied Dementia Studies<br>Rait, Greta; UCL, Primary Care<br>Robinson, Louise; Newcastle University, Institute for Ageing, Institute for Health and Society<br>Woodward-Carlton, Barbara; University of Bradford Faculty of Health Studies, Ccentre for Applied Dementia Studies<br>Young, John; University of Leeds, Academic Unit of Elderly Care and Rehabilitation<br>Downs, Murna; University of Bradford, Bradford Dementia Group

**Primary Subject Heading**:	Geriatric medicine
Secondary Subject Heading:	Health services research, General practice / Family practice, Health economics, Nursing
Keywords:	GERIATRIC MEDICINE, PRIMARY CARE, HEALTH ECONOMICS, Dementia < NEUROLOGY

For peer review only - http://bmjopen.bmj.com/site/about/guidelines.xhtml

I, the Submitting Author has the right to grant and does grant on behalf of all authors of the Work (as defined in the below author licence), an exclusive licence and/or a non-exclusive licence for contributions from authors who are: i) UK Crown employees; ii) where BMJ has agreed a CC-BY licence shall apply, and/or iii) in accordance with the terms applicable for US Federal Government officers or employees acting as part of their official duties; on a worldwide, perpetual, irrevocable, royalty-free basis to BMJ Publishing Group Ltd ("BMJ") its licensees and where the relevant Journal is co-owned by BMJ to the co-owners of the Journal, to publish the Work in this journal and any other BMJ products and to exploit all rights, as set out in our licence.

The Submitting Author accepts and understands that any supply made under these terms is made by BMJ to the Submitting Author unless you are acting as an employee on behalf of your employer or a postgraduate student of an affiliated institution which is paying any applicable article publishing charge ("APC") for Open Access articles. Where the Submitting Author wishes to make the Work available on an Open Access basis (and intends to pay the relevant APC), the terms of reuse of such Open Access shall be governed by a Creative Commons licence – details of these licences and which Creative Commons licence will apply to this Work are set out in our licence referred to above.

Other than as permitted in any relevant BMJ Author's Self Archiving Policies, I confirm this Work has not been accepted for publication elsewhere, is not being considered for publication elsewhere and does not duplicate material already published. I confirm all authors consent to publication of this Work and authorise the granting of this licence.

For peer review only

A pilot cluster randomised trial of an evidence-based intervention to reduce avoidable hospital admissions in nursing home residents (Better Health in Residents of Care Homes with Nursing - BHiRCH-NH study)

Elizabeth L Sampson,[1,2] Alexandra Feast,[1] Alan Blighe,[3] Katherine Froggatt,[4] Rachael Hunter,[5] Louise Marston,[5] Brendan McCormack,[6] Shirley Nurock,[1] Monica Panca,[5] Catherine Powell,[7] Greta Rait,[5] Louise Robinson,[8] Barbara Woodward-Carlton,[3] John Young,[9] Murna Downs[3]

Corresponding author
Elizabeth L. Sampson
UCL Division of Psychiatry
6th floor, Wing B, Maple House
149 Tottenham Court Road
London
W1T 7NF
02076799500/internal 09500
e.sampson@ucl.ac.uk

[1] Marie Curie Palliative Care Research Department, Division of Psychiatry, University College London, UK.
[2] Barnet Enfield and Haringey Mental Health Trust Liaison Psychiatry Team, North Middlesex University Hospital, London, UK.
[3] Centre for Applied Dementia Studies, University of Bradford, Bradford, BD7 1DP, UK.
[4] International Observatory on End of Life Care, Lancaster University, Lancaster, UK.
[5] Department of Primary Care and Population Health and Priment Clinical Trials Unit, University College London, London, UK.
[6] Divisions of Nursing, Occupational Therapy & Arts Therapies, School of Health Sciences, Queen Margaret University, Edinburgh, UK.
[7] School of Pharmacy and Medical Sciences, University of Bradford, Bradford, UK
[8] Newcastle University Institute for Ageing and Institute for Health & Society, Newcastle University, Newcastle upon Tyne, UK.
[9] Academic Unit of Elderly Care and Rehabilitation, University of Leeds, Bradford Institute for Health Research, Bradford, UK.

Word count: 5162

For peer review only

ABSTRACT

Objectives: To pilot a complex intervention to support healthcare and improve early detection and treatment for common health conditions experienced by nursing home (NH) residents.

Design: Pilot cluster randomised controlled trial.

Setting: 14 NHs (7 intervention, 7 control) in London and West Yorkshire.

Participants: NH residents, their family carers and staff.

Intervention: Complex intervention to support healthcare and improve early detection and treatment of urinary tract and respiratory infections, chronic heart failure and dehydration, comprising: 1) "stop and watch (S&W)" early warning tool for changes in physical health, 2) condition-specific care pathway and 3) Situation, Background, Assessment and Recommendation tool to enhance communication with primary care. Implementation was supported by Practice Development Champions, a Practice Development Support Group and regular telephone coaching with external facilitators.

Outcome measures: Data on NH (quality ratings, size, ownership), residents, family carers and staff demographics during the month prior to intervention and subsequently, numbers of admissions, accident and emergency visits and unscheduled GP visits monthly for 6 months during intervention. We collected data on how the intervention was used, healthcare resource use and quality of life data for economic evaluation. We assessed recruitment and retention and whether a full trial was warranted.

Results: We recruited 14 NHs, 148 staff, 95 family carers and 245 residents. We retained the majority of participants recruited (95%). 15% of residents had an unplanned hospital admission for one of the four study conditions. We were able to collect sufficient questionnaire data (all over 96% complete). No NH implemented intervention tools as planned. Only 16 S&W forms and 8 Care Pathways were completed. There was no evidence of harm.

Conclusions: Recruitment, retention and data collection processes were effective but the intervention not implemented. A full trial is not warranted.

For peer review only - http://bmjopen.bmj.com/site/about/guidelines.xhtml

Trial registration: ISRCTN74109734 (registered 6/11/2017)

https://doi.org/10.1186/ISRCTN74109734

Original protocol: *BMJ Open*. 2019;9(5):e026510. doi:10.1136/bmjopen-2018-026510

Keywords: cost analysis; dehydration; dementia; geriatric medicine; heart failure; nursing homes; primary care; respiratory infections; urinary tract infections

ARTICLE SUMMARY

Strengths and limitations of this study

- The intervention was adapted from a successful USA programme, co-designed with residents, family carers and staff to fit with the UK nursing home context
- Successful recruitment and retention of nursing homes, their staff, residents and family carers demonstrated the feasibility of our study methods
- It was challenging to collect reliable data on hospital admissions, ambulance and general practitioner visits
- The nature of the intervention in this cluster randomised trial meant that outcome data were not collected blind to allocation for intervention or control group.

INTRODUCTION

Currently in the United Kingdom (UK) more than 420,000 people aged over 65 years live in residential care, of which approximately 220,000 reside in care home with nursing (referred to in this paper as “nursing homes” (NHs)).[1] Older people living in NHs have complex healthcare needs with high levels of multi-morbidity, frailty and dementia. The King’s Fund[2] and British Geriatrics Society[3] have raised concerns about the quality of healthcare provision to NHs.

There has been a 63% increase in all-cause hospital admissions from NHs between 2011-2015.[4] As well as causing distress to residents, their families and staff, hospitalisation is expensive for health and social care systems, costing an estimated £1.2 billion per annum in the UK.[5] Hospital admission increases the risk of decline in functional ability, delirium, adverse events and prolonged stays.[6 7]

Ambulatory Care Sensitive Conditions (ACSCs) are “conditions that can lead to unplanned hospital admissions that may have been avoidable or manageable by timely access to medical care in the community”.[8] The conditions include angina, asthma, cellulitis, chronic obstructive pulmonary disease (COPD), congestive heart failure (CHF), dehydration, diabetes mellitus, gastroenteritis, epilepsy, hypertension, hypoglycaemia, urinary tract infections (UTI), pneumonia, severe ear, nose, and throat infections.[9] Four ACSCs contribute to a 30% of hospitalisations from NHs:[5] respiratory infections;[10 11-13] acute exacerbation of chronic heart failure;[14 15] urinary tract infections;[12 16] and dehydration.[12]

A number of interventions have been developed to enhance healthcare in NHs, falling broadly into two categories; single component interventions (predominantly advance care planning or single disease care pathways, e.g. for pneumonia) and multicomponent interventions which include enhancing staff knowledge and skills,[17] clinical guidance and decision-support tools (care pathways), engaging with families,[18] and input from geriatricians

For peer review only - http://bmjopen.bmj.com/site/about/guidelines.xhtml

or nurse practitioners.[19] Research highlights the importance of collaborative development of interventions with staff[20], residents and families,[18] and using local champions to support implementation.[15] "INTERACT" (Interventions to Reduce Acute Care Transfers), developed in the USA, focusses on managing acute changes in residents' condition and reduces transfers to acute hospitals.[15] We worked with stakeholders including NH staff and our family Carer Reference Panel (CRP), to develop and adapt INTERACT for use in the UK[21] (paper in preparation). The Promoting Action in Health Services (PARiHS) framework underpinned our co-design of implementation support and guidance[22] for the new "BHiRCH-NH" intervention.

Aim and objectives

Our aim was to indicate whether a definitive study of the BHiRCH-NH intervention is warranted.

Primary objective

To indicate whether the intervention was acceptable (including monitoring for serious adverse events (SAEs)) and feasible.

Secondary objectives

1. Establish whether consent procedures facilitate collection of sufficient individual-level data
2. Assess intervention fidelity
3. Assess the effectiveness of the implementation strategy and level of staff engagement with the intervention
4. Indicate whether the intervention would be sustainable outside the trial context
5. Assess potential primary and secondary outcome measures for a definitive trial
6. Measure completeness of data collection, documentation, return rate of questionnaires and assess potential primary and secondary outcomes for a definitive trial

For peer review only - http://bmjopen.bmj.com/site/about/guidelines.xhtml

7. Assess feasibility of collecting data for economic evaluation.

METHODS

Trial design

A pilot cluster randomised trial in NHs, conducted and reported as per the CONSORT guidance.[23] Detailed methods are described in the trial protocol paper.[21] The NH was the unit of allocation (seven intervention and seven control sites).

For peer review only

Patient and public involvement in research

The project was developed in collaboration with UK Dementia and Neurodegenerative Diseases Network (DeNDRoN) and Patient and Public Involvement representatives (SN and BW-C) who were grant co-applicants. Two Carer reference Panels (CRPs), ensured public involvement at all stages. Each comprised eight family carers of people with dementia and a person living with dementia, supported by Alzheimer's Society research volunteer network. They worked collaboratively on intervention design, recruitment and consent processes, accessibility of information leaflets, data collection, interpretation and dissemination.

Study population and eligibility criteria

Nursing homes

We recruited 14 NHs (8 in West Yorkshire and 6 in London) with adequate staffing to implement the intervention and support research. These were identified via local Clinical Research Networks, and the ENRICH (Enabling Research in Care Homes) Network, purposively selected to include a range of providers (large and small chains, independent providers), urban, suburban and rural. Managers, regional managers, or owners gave written permission. The intervention was an enhanced version of "usual care" implemented at NH level. Therefore, individual consent was not required and all staff were involved in delivering the intervention to all care home residents.

For peer review only - http://bmjopen.bmj.com/site/about/guidelines.xhtml

Individual participants

We invited all English-speaking staff and residents over 65 years and their carers (family members or friends) to participate in individual-level data collection until we had recruited approximately 20 residents and 10 staff from each NH. We excluded residents receiving end-of-life care or those who did not wish to be involved in research.

Consent procedures

Ethical approval was given by the Queen Square London Research Ethics Committee (reference 17/LO/1542).

Residents

The care home manager or deputy manager identified all potentially eligible residents. If necessary, we conducted a capacity assessment regarding trial participation, adhering to the UK Mental Capacity Act (2005). If the resident lacked capacity, we used a personal consultee (friend or family), or if not available, a professional consultee (health or social care staff with a professional relationship to the resident but no connection with the project). If a resident lost capacity during the study a consultee was found.

Family carers and staff

We invited a family carer and NH staff associated with residents recruited to the study to answer questionnaires and they gave informed consent for this.

INTERVENTION

The pilot trial ran for 10 months (November 2017-August 2018). Sites were set up in months one and two, in month three we collected pre-intervention (baseline) data over four weeks before the intervention was implemented. The intervention ran for 6 months with final follow up data collection and site closure occurring in month 10. We planned for the trial to run for

For peer review only - http://bmjopen.bmj.com/site/about/guidelines.xhtml

16 months from November 2017 but had to reduce follow-up due to delays in obtaining ethical approvals. Thus, timing of data collection differs from our protocol.[21]

Implementation support

This was developed consistent with the PARIHS Framework to ensure implementation, matched individual contexts. It was important for Practice development Champions (PDCs) to decide on how they approached this, given the philosophy of quality collaboratives and 'champions' in place. This enhanced the role of PDCs in developing the approach each wanted to adopt.

Workshop

The research team delivered a one-day workshop to two PDCs from each NH comprising an introduction to four key ACSCs (respiratory and urinary tract infections, dehydration, acute exacerbation of chronic heart failure) and elements of how to bring about organizational change. We gave an overview of intervention materials.

Ongoing implementation support

1. We supported PDCs with a project handbook and the *Practice Development Workbook for Nursing, Health and Social Care Teams: Resources for Health and Social Care Teams*.[24]
2. We expected PDCs to establish a Practice Development Support Group (PDSG) to support their work in the NH. This "quality collaborative" approach[25] involves diverse stakeholders working together to close the gap between actual and potential practice.
3. We offered monthly telephone coaching to the PDCs by senior nurse researchers on our team (BMcC and KF).

The intervention (BHiRCH-NH)

This consisted of three key components, adapted from the INTERACT programme[26] and paper-based as UK NHs have variable use of electronic records:

For peer review only - http://bmjopen.bmj.com/site/about/guidelines.xhtml

1. *Stop and Watch Early Warning Tool*: Care assistants or nurses used this when they noted a change in a resident's condition. They circled observed changes, notified the nurse and placed the tool in the resident's NH records.
2. *Care Pathway*: This was a two-step clinical guidance and decision support system, focussing on symptoms and signs of four key ACSC conditions (acute exacerbation of chronic heart failure, respiratory and urinary tract infections, dehydration). The initial "Primary" assessment comprised screening questions with the potential to trigger a more detailed "Secondary" assessment. If the Primary or Secondary Assessment result was ambiguous, the *Care Pathway* was to be administered at 6-hour intervals, until concerns had resolved and/or appropriate intervention was instigated. The nurse recorded the outcome of the Primary and Secondary Assessment and their care plan in the resident's records and decided on the next course of action. This may have included further monitoring using the *Stop and Watch Early Warning Tool*, treatment initiated in the NH, or communication with primary care using the *SBAR*. Copies of the completed *Care Pathway* were kept with the resident's record.
3. *The SBAR method:* A structured method for communicating critical information to primary care used by nurses to seek primary care intervention for the resident after the *Care Pathway* indicated a risk of decline.

Treatment as usual (TAU) nursing homes

Residents received usual care according to existing local policy and practice. We permitted all medications and treatments.

Data collection

Nursing homes

A Research Facilitator, employed by the NH provided pseudo-anonymised data at the NH level. We collected data for four weeks pre-intervention (baseline) including staff turnover and the number of beds available to new residents. For four weeks pre-intervention and then

For peer review only - http://bmjopen.bmj.com/site/about/guidelines.xhtml

monthly for 6 months we documented the total number of contacts with GPs, ambulances, A&E attendances and hospital admissions.

Residents

We collected data pre-intervention on age, gender, ethnicity, marital status and highest level of education. Functional status was measured using the Barthel Index[27] pre-intervention, and at 6 months. We collected data for the month pre-intervention and then for the following 6 months on healthcare use and QoL data for health economic analysis, hospital admission overall and admissions for the four ACSCs of interest, ambulances called, out-of-hours GP visits or telephone contacts, A&E attendances and deaths.

Family carers

Pre-intervention we collected data on socio-demographics including age, gender, ethnicity and marital status and preferred role, i.e. how involved they would like to be with the resident's medical care.

Staff

For contextual understanding we documented pre-intervention; staff age, gender, and education level and characteristics of their work (qualifications, role, length of employment, shift pattern and first language). Pre-intervention and at 6 months we measured the extent to which they perceived the organisation supported person centred care (Organisational Support for Person-centred Care[28] (P-CAT)) and the Nurse Ratings of Communication with Primary Care Questionnaire.[29]

Outcomes

We collected data in three domains: 1) individual-level data on NH residents, their carers and staff where consent had been obtained, 2) system-level data collected by a Research

For peer review only - http://bmjopen.bmj.com/site/about/guidelines.xhtml

Facilitator to provide pseudo-anonymised data at the NH level, and 3) process data collected by the study team (table 1).

Table 1: Summary of data collected, outcome measures and time schedule

**Data collected and tool used**		**Pre-intervention**	**Monthly**	**6 months**
Resident				
Socio-demographics	Age, gender, ethnicity, marital status, highest level of education	S	-	-
Service use in the prior month	Client Service Receipt Inventory (CSRI). Calculates service and total care costs	S	S	-
Functional status	The Barthel Index	S	-	S
Resident quality of life-self rated	EQ-5D-5L self-rated health index and visual analogue scale of current health state	P	-	P
Resident quality of life-proxy rated	EQ-5d-Proxy family carer or staff member view of the resident's QoL	FC/S	-	FC/S
Family carer				
Socio-demographics	Age, gender, ethnicity, marital status, years of schooling, highest level of education	FC	-	-
Quality of life	EQ-5D-5L	FC	-	FC
Preferred role	How much and how they like to be involved in the residents' care	FC	-	-
Staff				
Staff socio-demographics	Age, gender, ethnicity, number of years of education,	R	-	-
Staff work characteristics	Highest qualification, role in nursing home, length of service, shift pattern, first language	R	-	-
Organisational support for person-centered care	The Person-Centred Care Assessment Tool (P-CAT)	S	-	S
Communication with primary care	Nurse-GP Communication Needs Assessment Questionnaire	S	-	S
Perceived knowledge and skills for early detection in changes in health	Developed from feasibility study. Assesses key knowledge and skills needed to implement the intervention. Rated on 5-point Likert scale.	S	-	S
**System-level data**				
Number of hospital admissions	Respiratory infection, exacerbation of CHF, UTI and dehydration	S	S	-
"Avoidability" of admissions	Structured Implicit Record Review (SIRR; Saliba et al., 2000)	S	S	-
Use of Primary assessment tool	Respiratory infection, exacerbation of CHF, UTI and dehydration?	S	S	-
Use of Secondary assessment	Respiratory infection, exacerbation of CHF, UTI and dehydration	S	S	-
Out of hours GP contacts	GP visits or telephone contact	S	S	-
Ambulances and hospital use	Number of hospital admissions, A&E attendances and readmissions	S	S	-
Deaths in the last calendar month		S	S	-
Staff turnover		S	-	-
Nursing home occupancy level	Number of available beds to new residents	S	-	-

Measure assessed by: P, participant; FC family carer; R researcher; S, nursing home staff

For peer review only - http://bmjopen.bmj.com/site/about/guidelines.xhtml

Primary objective

To ascertain whether the intervention was acceptable and feasible we collected data monthly from the NH on intervention use in practice; the number of "Stop and Watch" Early Warning Tools and Primary (initial screening) and Secondary (more detailed) assessment tools completed. Where we had permission to collect individual-level resident data, we monitored participants monthly for SAEs defined as "any untoward occurrence that resulted in death, was considered life-threatening at the time of the event, required hospitalisation or prolongation of existing hospitalisation, resulted in persistent or significant disability or incapacity or was any other important medical condition".

Secondary objectives

1. *Establish whether consent procedures facilitate collection of sufficient individual-level data*

We collected data on consent and recruitment rates of residents, carers and staff.

2. *Assess intervention fidelity*

To explore intervention fidelity two nurse researchers aimed to review a convenience sample of five records for residents admitted to hospital, or received treatment in the NH, for ACSCs. They used a free-text review sheet to record references to trial intervention tools (Stop & Watch, the Care Pathway and SBAR) and assessed compliance with the care pathways. We noted where NHs made amendments to the structure or content of the care pathway.

3. *Assess the effectiveness of the implementation strategy and level of staff engagement with the intervention*

PDCs were offered a monthly support phone call with the research team to reflect on activities and achievements. They kept an activity log of work with Practice Development Support Groups to document the level of facilitation required to support the implementation process. Qualitative interviews were conducted to better understand the barriers and facilitators to implementation. Learning from the implementation strategy will be presented in a separate paper.

For peer review only - http://bmjopen.bmj.com/site/about/guidelines.xhtml

For peer review only

4. Indicate whether the intervention would be sustainable outside the trial context

Data from objectives 2-3 were considered by the independent project steering group at the end of the study.

5. Assess potential primary and secondary outcomes for a definitive trial

To explore the impact of enhanced healthcare in the NH we documented hospital admissions for the four key ACSCs (acute exacerbation of chronic heart failure, respiratory and urinary tract infections, dehydration). Number (%) of residents requiring one or more ambulance calls, one or more unscheduled out of hours GP visits or phone calls and having one or more accident and emergency department visits were documented as potential secondary outcomes.

We tested the assumption that a hospitalisation for an ACSC was a proxy for an avoidable hospital admission using the Structured Implicit Record Review (SIRR) tool.[30] Two independent experts (geriatrician and community nurse) used the SIRR, which takes account of the resident's pre-existing health, any advance directives and the care options available at the time to assess the "avoidability" of the admission.

6. *Measure completeness of data collection and documentation, return rate of questionnaires*

We assessed completeness of outcome measures, data collection and return rate of questionnaires.

7. Assess feasibility of collecting data for economic evaluation

We collected data on resident service use with the Client Service Receipt Inventory[31] (CSRI), completed pre-intervention and then monthly for 6 months. Data on quality of life (QoL) was collected using the self-completed EQ-5D-5L[32] questionnaire where participants had the capacity to do this. Where a resident could not complete the EQ-5D-5L questionnaire, the proxy version was completed by the carer or a member of staff. We also collected data on carer QoL using the EQ-5D-5L questionnaire. All QoL data were collected pre-intervention and at 6 months.

For peer review only - http://bmjopen.bmj.com/site/about/guidelines.xhtml

Sample size

This was a pilot study so no sample size calculation was conducted. The number of care homes was chosen on pragmatic grounds to allow testing of study procedures and variability in settings.

Randomisation

NHs were randomised prior to intervention; 4 in West Yorkshire and 3 in Greater London (7 total) to the intervention and 4 in Yorkshire and 3 in Greater London (7 total) to "usual care", stratified by location. We used the SAS statistical programme to generate a randomisation list drawn up by a clinical trials unit statistician not otherwise involved in the study.

Blinding

This was not feasible for staff collecting data. Statisticians and health economists were blinded to allocation. The randomisation variable was supplied to them unlabelled, and the main analysis was completed using this.

Data management

All data was entered onto paper case report forms (CRFs) and then into an encrypted password protected database in accordance with the UK Data Protection Act and General Data Protection Regulation (GDPR). We followed a standardised process for database lock.

Statistical methods

As a pilot study, analyses are mainly descriptive (counts, means, standard deviation (SD), medians with interquartile range (IQR)) focussing on recruitment, participant characteristics, other baseline and outcome variables, loss to follow-up and tabulation of SAEs. We summarised completeness of data on outcome measures and questionnaire response rates.

For peer review only

Economic evaluation

Our aim was to provide a preliminary estimate of the costs, effects and relative cost-effectiveness of the intervention compared to TAU using accepted economic evaluation methods. We calculated costs associated with the intervention, including costs of staff training and implementation. Resource use associated with hospital admissions, primary care and other NHS and social care services were collected using the CSRI and costed[33-35] from the NHS/ personal social services (PSS) perspective. All costs are reported in 2016/2017 British Pounds (£). Quality-adjusted life years (QALYs) for residents were calculated using the EQ-5D-5L questionnaire and associated algorithms[36 37] mapping the 5L descriptive system data onto the 3L valuation set as recommended by the National Institute for Health and Care Excellence (NICE).[38 39] We report only results based on QALYs calculated using the resident self-completed EQ-5D-5L questionnaire as these had higher return. There was no discounting of costs or QALYs given that they were reported over 6 months only. Multivariate imputation by chained equation for missing data, generating 20 imputed data sets were used. For each of these, we ran 1,000 bootstrap replications using non-parametric bootstrapping. Bootstrap results were combined to calculate the mean values for costs and utilities and the standard errors around the imputed values, used to calculate 95% CI around point estimates. To report the probability that the intervention is cost-effective compared to TAU for a range of values of willingness-to-pay (WTP) for a QALY gained, the bootstrap results have been used to generate a cost-effectiveness acceptability curve (CEAC)[40] and the probability that the intervention is cost-effective compared to TAU at a £20,000 for a QALY gained reported.

RESULTS

Here, we give trial results with full data given in the supplementary file appendices to ensure transparency of reporting. In depth evaluation of implementation will be published separately.

For peer review only - http://bmjopen.bmj.com/site/about/guidelines.xhtml

Recruitment

Nursing homes

We recruited the target number of 14 NHs and randomised as planned; seven intervention (3 London, 4 Yorkshire) and seven control (3 London and 4 Yorkshire). One Yorkshire intervention NH dropped out during pre-intervention as it was closed by its owners. A further intervention NH in London dropped out following PDC training as they were unable to implement the intervention. Most NHs were privately managed with a median 50 residents (IQR 34, 68). The majority (73%) were “Dementia Registered”. In terms of their CQC ratings, one home (7%) was “outstanding”, eleven (79%) “good” and two (14%) “required improvement” (supplementary file, appendix 1).

Residents

We recruited 237 residents (figure 1), two thirds were female, predominantly white (90%) with a median age of 86 years (IQR 80, 91).

Figure 1 here

The median Barthel Index score was low at 27 (IQR 9, 64) indicating a high level of dependency in activities of daily living. Only six residents (3%) had an admission for an ACSC in the pre-intervention period, 11 (5%) residents had at least one ambulance called and 12 (5%) had an unscheduled GP visit or telephone contact (table 2).

Family carers

We recruited 91 family carers, two thirds were female (supplementary file, appendix 2). Median age was 63 years (IQR 57, 71). Most (91%) wished to be involved in noticing early changes in the resident’s health.

Table 2: Characteristics of residents and family carers

Characteristic	Cohort	TAU	BHiRCH-NH
	n or median (% or IQR)		
**RESIDENTS**			
**Demographics**	N=234	N=137	N=97
Male	73 (31)	46 (34)	27 (28)
Age	86 (80,91)	86 (80, 91)	84(78, 91)
**Ethnicity**	N=225	N=131	N=94
White	203 (90)	117 (89)	86 (91)
Black	14 (6)	9 (7)	5 (5)
Asian	5 (2)	4 (3)	1/(1)
Other	3 (1)	1 (1)	2(2)
**Marital status**	N=223	N=127	N=96
Married or cohabiting	49 (22)	28 (22)	21 (22)
Single	59 (26)	40 (32)	19 (20)
Divorced or widowed	115 (52)	59 (46)	56 (58)
**Education**	N=184	N=133	N=71
Completed years of education	11 (9,12)	11(10, 12)	11(9, 11)
No qualifications or GCSE or equivalent	107 (58)	63 (56)	44 (62)
A Level/NVQ/ HNC/ HND or equivalent	18 (10)	11 (10)	7 (10)
Degree or higher degree	23 (13)	14 (12)	9 (13)
Other qualification	36 (20)	25 (22)	11 (15)
**Function**			
Barthel Index score	27 (9, 64)	27(9, 66)	30(8, 63)
**CARERS**			
**Demographics**	N=91	N=56	N=35
Male	31 (34)	17 (30)	14 (40)
Age	63 (57, 71)	62(57, 71)	64(58, 74)
**Ethnicity**	N=87	N=52	N=35
White	72 (83)	43 (83)	29 (83)
Black	12 (14)	7 (13)	5 (14)
Asian	3 (3)	2 (4)	1(3)
**Marital status**	N=87	N=53	N=34
Married or cohabiting	65 (75)	36 (68)	29 (85)
Single	10 (11)	8 (15)	2 (6)
Divorced or widowed	12 (14)	9 (17)	3 (9)
**Education**	N=86	N=53	N=33
Completed years of education	11 (11,12)	12(11, 13)	11(11, 12)
No qualifications or GCSE or equivalent	35 (41)	21 (40)	14 (42)
A Level/NVQ/ HNC/ HND or equivalent	13 (15)	9 (17)	4 (12)
Degree or higher degree	26 (30)	14 (26)	12 (36)
Other qualification	12 (14)	9 (17)	3 (9)
**Preferred role**	N=87	N=52	N=35
Noticing early signs of changes in health	79 (91)	49 (94)	30 (86)
Informing staff about early signs of changes in health	77 (89)	48 (92)	29 (83)
Educating staff about how early signs of changes in health present	51 (59)	28 (54)	23 (66)
Educating care staff about health history of their family member	57 (66)	33 (63)	24 (69)
Prefer not to be involved	5 (6)	2 (4)	3 (9)
Other	18 (21)	9 (17)	9 (26)

For peer review only - http://bmjopen.bmj.com/site/about/guidelines.xhtml

Staff

We recruited 132 staff (supplementary file, appendix 3), with a median age of 42 years (IQR 30, 53), 12% were male, 50% of nurses spoke English as a first language and 59% of staff described themselves as white. Most staff (71%) had worked at the care home for a year or more and 30% were qualified nurses (supplementary file, appendix 4). Scores on the P-CAT scale (possible range 13-65) were generally positive with a median score of 49 (IQR 46, 53) (supplementary file, appendix 4). Most nurses were positive about the quality of communications they had with GPs (supplementary file, appendix 5) and their self-rated knowledge and skills (supplementary file, appendix 6).

Primary objective-feasibility and acceptability

Use of the intervention

Across the five intervention NHs, only sixteen Stop and Watch forms were completed of which eleven came from a single NH. Eight Care Pathways were reported as completed but only three were located by the study team. There was a median of one Stop and Watch form (IQR 0,3) and a median of zero Care Pathways (IQR 0,2) completed per month. In a few cases, routine clinical observations (e.g. temperature, blood pressure etc.) were carried out, but were not reported systematically or presented as part of a coherent assessment plan. One home had a policy of recording routine observations once per month.

Serious adverse events

There were no differences in SAEs between TAU and intervention groups. There were 104 SAEs in 74 residents during the study and 33 residents died (19 TAU and 14 intervention). Of the 104 SAEs, hospitalisation (any cause) was the most common (N= 50) (supplementary file, appendix 7).

For peer review only - http://bmjopen.bmj.com/site/about/guidelines.xhtml

Secondary objectives

1. Resident consent procedures and collection of sufficient individual level data

We screened 680 residents, 557 met eligibility criteria and 245 were recruited (35% recruitment). Taking into account that two NHs dropped out, leaving 12 in the study, our target of 240 residents (20 residents per care home) was reached. Most eligible residents (364, 65%) did not have capacity to consent to participate in the study. Of recruited residents 73% completed the study (supplementary file, appendix 8).

2. Assess intervention fidelity

It was not possible to assess fidelity to the intervention as the intervention was not implemented as intended and the documentation required to assess fidelity was not available.

3. Assess the effectiveness of the implementation strategy and level of NH staff engagement with the intervention

The implementation strategy was not effective and NH staff did not engage with or use the intervention tools. Data and learning from implementation will be presented in a separate paper.

4. Investigate whether the intervention would be sustainable outside the context of a trial

The intervention was not widely used; 16 Stop and Watch forms were completed and we found eight completed Care Pathway documents. We concluded the intervention in its current form was not sustainable.

5. Assess potential primary and secondary outcome measures for a definitive trial

Rates of hospitalisation for ACSCs

At baseline the rates of hospitalisation for ACSCs (respiratory infection, exacerbation of CHF, UTI and dehydration), our potential primary outcome, were low and 0.4% of the cohort

For peer review only - http://bmjopen.bmj.com/site/about/guidelines.xhtml

had an admission for respiratory infections, 1% for UTI, none for dehydration and 0.4% had an admission for chronic heart failure in the month before the trial started. There were six admissions in total from the 235 residents in the study. Considering the whole six-month study period, 25 study participants (15%) had an unplanned hospital admission for one of the ACSCs. The low rates of unplanned hospital admission (per 100 person months) for these ACSCs suggest this is not an optimal primary outcome measure for a future study (table 3). Considering secondary outcome measures for a definitive trial, we found a slightly higher proportion of participants (n=38, 16%) had an accident or emergency attendance during the follow up period, 42 (18%) had at least one ambulance called, 29 (12%) had an unscheduled (out-of-hours) GP visit and 21 (11%) died. The incidence of these events was still relatively low and not sufficiently frequent for a definitive study outcome.

Hospitalisation for an ACSC as a proxy for avoidable hospital admission

We intended to use the Structured Implicit Record Review (SIRR) tool to assess the appropriateness of 30 resident admissions for one of the four ACSCs, but it was not always possible to identify the reasons for admission and these were low overall. We therefore expanded our sample to include any hospital admission for any cause. One NH had no eligible residents, because those admitted to hospital had died and/or their care records were no longer available. We therefore also included residents who died in hospital, as long as their records were still available. We were able to assess 10 admissions from a total of 24 which occurred during the study period. Using the SIRR tool, three of the ten admissions assessed were deemed to be potentially avoidable. None of the NH care records provided a complete picture of residents' health in the period leading up to admission. From this review an ACSC admission was not a reliable proxy measure for an "avoidable admission".

For peer review only - http://bmjopen.bmj.com/site/about/guidelines.xhtml

Table 3. System-level outcome data

	Pre-intervention			Over 6 month follow up period	
	Whole cohort	TAU	BHiRCH-NH	TAU	BHiRCH-NH
	n or median (% or (IQR)				
**Study cohort**	N=235	N=139	N=96	N=139	N=96
At least one admission in the last month	6 (3)	2 (1)	4 (4)	19 (14)	16/96
Respiratory infection admission	1 (0.4)	0	1 (1)	8 (6)	5/96
Urinary tract infections admission	2 (1)	1 (1)	1 (1)	5 (4)	2/96
Dehydration admission	0	0	0	0	1/96
Congestive heart failure admission	1 (0.4)	0	1 (1)	0	1/96
At least one ambulance called	11(5)	4 (3)	7 (7)	20 (14)	22/96
At least one out of hours GP visit or telephone contact	12 (5)	11 (8)	1 (1)	14 (10)	15/96
At least one Accident and Emergency attendance	10 (4)	6 (4)	4 (4)	17 (12)	21/96
Died	1 (0.4)	1 (1)	0		
**Care home data**					
Number hospital admissions	3 (2, 5)	4 (2, 7)	3 (2, 4)	12 (12, 16)	12 (7, 18)
Number of ambulances called	3 (2,6)	4 (2,9)	3 (2, 6)	12 (11,17)	19 (7, 22)
Unscheduled (out of hours) GP visits or telephone contacts	1 (1,3)	2 (1,3)	1 (1, 3)	8 (7,13)	9 (4, 25)
Accident and Emergency attendances	3 (2,5)	3 (1,4)	3 (2, 6)	12 (11,13)	8 (7, 13)
Rate of hospital admissions per 100 person months		-	-	5.3 (2.3, 8.3)	5.9 (1.7, 7.1)
Rate of ambulances called per 100 person months		-	-	5.7 (2.3, 8.0)	6.0 (2.0, 9.3)
Rate of unscheduled (out of hours) GP visit or contacts per 100 person months		-	-	2.5 (1.8, 4.0)	5.1 (1.1, 6.0)
Rate of Accident and Emergency attendances per 100 person months		-	-	4.3 (2.5, 8.3)	3.9 (2.0, 5.6)

6. Completeness of data collection and documentation, return rate of questionnaires

Care staff-related data

We collected data on most recruited care staff at baseline (N=132). For example, 129 care staff gave demographic detail (98%) and 127 (96%) completed the P-CAT scale. Attrition in response to these scales was secondary to NHs dropping out of the study, rather than staff being unwilling or unable to complete the forms.

Resident-related data

Demographic and functional ability data were available on most residents at baseline (N=235) including gender (99%), ethnicity (95%), marital status (98%) and functional ability (Barthel Index) (98%). We cannot be sure that no admissions or visits to acute hospital were

For peer review only - http://bmjopen.bmj.com/site/about/guidelines.xhtml

missed as NHs did not have centralised systems for collecting these data.

7. Cost and outcome data for use in an economic evaluation, key cost components and probability of cost effectiveness

One NH withdrew after randomisation and therefore, we considered the cost of intervention to be £0 (as “per randomised” approach). Assuming the intervention is offered to all 89 residents randomised to the intervention group, the mean cost per resident would be £74 (95%CI £64 to £84). During follow-up, there were no significant differences in the majority of the components of healthcare resource use between the intervention and TAU groups (supplementary file, appendices 9and 10). Differences in mean utility values and QALYs were not statistically significant when carers assessed their QoL and therefore, no further analysis has been performed. The mean total cost of healthcare resource use per resident over 6 months was £1,458 (95%CI £1,351 to £1,566) in the intervention group and £1,233 (95%CI £1,171 to £1,295) in the TAU group (supplementary file, appendix 11). Non-parametric bootstrapping after multiple imputation produced a mean total cost per resident in the intervention group of £1,479 (95%CI £757 to £2,200), compared with £1,271 (95%CI £975 to £1,566) in the TAU group. The mean difference in cost between the BHiRCH-NH and TAU group was £208 (95%CI -£561 to £977). Non-parametric bootstrapping after multiple imputation produced 0.315 (95%CI 0.304 to 0.326) QALYs in the intervention group and 0.298 (95%CI 0.290 to 0.307) QALYs in the TAU group, generating a mean difference in QALYs of 0.016 (95%CI 0.003 to 0.300) which was statistically significant (table 4).

For peer review only

Table 4. Cost-effectiveness of BHiRCH-NH intervention versus TAU: complete case and imputed data analyses

	Incremental cost		QALY gained	
	**Mean**	**(95% CI)**	**Mean**	**(95% CI)**
Base case*	£208	-£561 to £977	0.016	0.003 to 0.300
Complete case^	£352	-£745 to £1,448	0.018	-0.012 to 0.048

*Data include values imputed using multiple imputation with standard errors corrected to account for uncertainty in the imputed values. QALYs gained are adjusted for baseline utility values and care home clustering. The incremental costs are adjusted for costs in the 1 month period prior to baseline and care home clustering. ^As for the base case analysis except there is no multiple imputation for missing data

The incremental cost per QALY gained of BHiRCH-NH versus TAU was £12,633. Residents receiving the intervention accrued a non-significantly higher cost, and a very small increase in QALYs; the intervention has a 65% probability of being cost-effective at a WTP of £20,000 (supplementary file, appendix 12).

DISCUSSION

Our cluster randomised pilot trial of the BHiRCH-NH intervention in 12 NHs found our study processes were effective. We successfully recruited, retained and obtained individual-level data from residents, staff and family carers. Data on adverse events did not suggest the intervention caused harm. Our Carer Reference Panel worked with us throughout, advising on study set-up, engaging with homes and potential participants, data collection and contributing to data analysis and interpretation. However, despite excellent recruitment and retention, there was limited engagement with the intervention tools and the support offered for their implementation. The lack of use of the intervention coupled with the economic analysis means that we would not recommend a definitive randomised controlled trial of the BHiRCH-NH intervention. We focussed on four key ACSCs (dehydration, respiratory and urinary tract infections and exacerbation of chronic heart failure) because they are common causes of potentially avoidable hospital admissions and a significant area of health policy focus. There is lack of national comparative data, but we found lower hospitalisation rates for

For peer review only - http://bmjopen.bmj.com/site/about/guidelines.xhtml

these conditions than we expected. Categorising admissions to hospital is complex and people may present with broader symptoms i.e. delirium or falls. ACSCs may be more of an administrative label to be used in large data analyses rather than a sensitive tool for assessing the "avoidability" or otherwise of acute hospital admissions.

Implementing practice change in NHs can be challenging. Despite offering monthly telephone support calls during the study at times agreed with PDCs, they often couldn't speak due to changes in duty rosters, last-minute leave or being too busy. Whilst the INTERACT programme, from which our BHiRCH-NH intervention was adapted, demonstrated reductions in all-cause hospitalisations among actively participating NHs, a subsequent larger randomised controlled implementation trial in 85 USA NHs had no effect on emergency department visits or hospital admissions.[15] Resources, competing demands and instability of NH leadership were all barriers to successful to INTERACT implementation[41] and it is likely that these factors were also present in our study.

Our study has limitations. We recruited NHs, residents, staff and family carers to target, although two NHs dropped-out from the intervention arm. Our NHs may be atypical as 79% were rated as "good" by the CQC, compared to the UK national average of 73%. Residents in our sample were broadly representative of the UK NH population in terms of age and gender.[42] Monthly visits from research fieldworkers and the appointment of Research Facilitators who were existing NH staff fostered positive relationships between NHs and the research team and facilitated access to NH records. We cannot verify that data on hospital attendances, staffing and support from external health care services is complete, as most NHs did not routinely record hospital admissions or ambulance and GP call-outs. Staff self-rated their knowledge and skills regarding health conditions and their communication with primary care and research assistants collecting outcome data were not blind to NH allocation. We assumed we could use admission for ACSCs as a proxy for avoidable admissions, but even using the SIRR tool[43] it was difficult to identify whether an admission

For peer review only - http://bmjopen.bmj.com/site/about/guidelines.xhtml

was for an ACSC or not. Future studies should include consent to access hospital records so this can be more thoroughly assessed.

Current UK policy focus on reducing hospitalisations and enhanced health care in NHs has led to significant levels of activity in local health and social care services. Research in such a fast-moving landscape is challenging. NHS England's demonstration projects "Vanguards" and increasing numbers of local quality improvement initiatives means "usual care" will be improving, making trials challenging to conduct. Although our intervention was co-designed with staff, this involved an extra burden of observation and documentation, which was difficult for staff who were already pressed for time. Recent studies conducted in care homes suggest that a higher level of support and facilitation may be required for effective implementation; meetings involving all nursing home staff may be more successful.[44] The Palliative Care for Older People Steps to Success Programme (PACES), conducted in 78 NHs across seven countries, which involved a complex intervention to improve end of life care, did not improve resident comfort or staff knowledge despite using a train-the-trainer approach.[45] However, the Well-Being and Health for People with Dementia (WHELD) programme was successfully implemented in 69 UK care homes and showed a significant effect on reducing agitation possibly through a much higher intensity of external facilitation that may not be sustainable or cost effective.[46] Since we designed our intervention and conducted our pilot trial, knowledge on improving healthcare for care home residents has increased. Key components for resident health care, identified in the 2017 "Optimal" study were GP involvement supported by integrated external services.[47] These findings are reflected by emerging evidence that change to the wider health and social care system may be more effective in reducing the number of NH residents admitted to hospital.[48]

CONCLUSIONS

Our co-designed complex intervention of tools for early detection, monitoring and communication of change in residents' health does not warrant a future definitive trial

For peer review only - http://bmjopen.bmj.com/site/about/guidelines.xhtml

because it was not implemented in practice. However, it was important that this pilot trial was conducted as this has avoided the risk of embarking on a full trial and subsequent waste of resources and time.[49] Our study contributes to learning in the under-researched but vital field of NH research.

DECLARATIONS

Acknowledgements: The authors would like to thank the members of our carer reference panels and the Alzheimer's Society research network volunteers. We are grateful to the nursing homes that supported our feasibility study. We are grateful to the Clinical research Network staff; Mary-Jo Doyle, Deborah Cooper, Kim Williams, Sally Gordon and Helen Permain and others who assisted with data collection Justin Chan, Ayesha Dar, Luiza Grycuk, Dr Adam Hughes, Dr Maria Ivanov and Angela Richardson. We would like to thank the study steering group (Dr Najma Siddiqi (Chair), Prof Tom Dening, Bert Green, Mike Bradburn, Laura Mandefield and members of the international advisory group (Prof Finbarr Martin (Chair), Prof Barbara Bowers, Prof John Gladman, Prof Claire Goodman, Prof Martin Green, Prof Raymond Koopmans, Prof Julienne Meyer, Prof Des O'Neill, Prof Jo Ouslander, Dr Hilary Rhoden, Jenny Adams, Prof Lynn Chenoweth, Prof Graham Stokes, Louise Taylor, Gavin Terry, Steve Williams). .

Competing interests statement: none declared

Funding: This work was supported by the UK NIHR grant number RP-PG-0612-20010. The views expressed are those of the author(s) and not necessarily those of the NHS, the NIHR or the Department of Health and Social Care. The funding body had no role in the design of the study, collection, analysis, and interpretation of data and in writing the paper.

For peer review only - http://bmjopen.bmj.com/site/about/guidelines.xhtml

Author contributions: ELS, AF, AB, KF, RH, LM, BMcC, SN, CP, MP, GR, LR, BW-C, JY, MD were involved in development of the intervention and made substantial contributions to the concept and design of the study and protocol. LM, MP and RH analysed the data. ELS and AF wrote the paper with assistance from CP, AF, AB, KF, RH, LM, BMcC, SN, MP, GR, LR, BW-C, JY, MD critically revised the manuscript and approved the final version.

Provenance and peer review: not commissioned; externally peer reviewed

Data sharing: The datasets generated and/or analysed during the current study are not publicly available, the small number of NHs means that data may be identifiable. Data are available from the corresponding author on reasonable request.

For peer review only

REFERENCES

1. Laing and Buisson. Care of Older People UK Market Report. 28th ed, 2017.
2. Tian Y, Dixon A, Gao H. Emergency hospital admissions for ambulatory care-sensitive conditions: identifying the potential for reductions: King's Fund, 2012.
3. British Geriatric Society. Quest for quality in care homes: British Geriatrics Society Joint Working Party Inquiry into the Quality of Healthcare Support for Older People in Care Homes: A Call for Leadership, Partnership and Quality Improvement. London: British Geriatric Society, 2011.
4. Bardsley M, Blunt I, Davies S. Is secondary preventive care improving? Observational study of 10-year trends in emergency admissions for conditions amenable to ambulatory care. *BMJ Open* 2013;3(1) doi: 10.1136/bmjopen-2012- 002007
5. Kings Fund. Data briefing: emergency hospital admissions for ambulatory care sensitive conditions 2012 [Available from: https://www.kingsfund.org.uk/sites/default/files/field/field_publication_file/data-briefing-emergency-hospital-admissions-for-ambulatory-care-sensitive-conditions-apr-2012.pdf accessed 20/02/2020.
6. Mukadam N, Sampson EL. A systematic review of the prevalence, associations and outcomes of dementia in older general hospital inpatients. *IntPsychogeriatr* 2010:1-12.
7. Watkin L, Blanchard MR, Tookman A, et al. Prospective cohort study of adverse events in older people admitted to the acute general hospital: risk factors and the impact of dementia. *IntJGeriatrPsychiatry* 2012;27(1):76-82. doi: 10.1002/gps.2693 [doi]
8. Carter MW, Porell FW. Vulnerable populations at risk of potentially avoidable hospitalizations: the case of nursing home residents with Alzheimer's disease. *American journal of Alzheimer's disease and other dementias* 2005;20(6):349-58. [published Online First: 2006/01/07]
9. Grabowski DC, O'Malley AJ, Barhydt NR. The costs and potential savings associated with nursing home hospitalizations. *Health affairs (Project Hope)* 2007;26(6):1753-61. doi: 10.1377/hlthaff.26.6.1753 [published Online First: 2007/11/06]
10. Purdy S, Griffin T, Salisbury C, et al. Ambulatory care sensitive conditions: terminology and disease coding need to be more specific to aid policy makers and clinicians. *Public Health* 2009;123:169-73.
11. Walsh E, Wiener J, Haber S. Potentially avoidable hospitalizations of dually eligible Medicare and Medicaid beneficiaries from nursing facility and Home- and Community-Based Services waiver programs. *Journal of the American Geriatric Society* 2012;60(5):821-9. doi: 10.1111/j.1532-5415.2012.03920.x
12. Carter M, Porell F. Vulnerable populations at risk of potentially avoidable hospitalizations: the case of nursing home residents with Alzheimer's disease. *American Journal of Alzheimer's Disease and Other Dementias* 2005;20(6):349-58.
13. Krüger K, Jansen K, Grimsmo A. Hospital admissions from nursing homes: Rates and reasons. *Nursing Research and Practice* 2011 doi: 10.1155/2011/247623
14. Ouslander J, Diaz S, Hain D. Frequency and diagnoses associated with 7- and 30- day readmission of skilled nursing facility patients to a nonteaching community hospital. *Journal of the American Medical Directors Association* 2011;12(3):195-203. doi: 10.1016/j.jamda.2010.02.015
15. Ouslander J, Lamb G, Tappen R. Interventions to reduce hospitalizations from nursing homes: evaluation of the INTERACT II collaborative quality improvement project. *Journal of the American Geriatric Society* 2011;59(4):745-53. doi: 10.1111/j.1532-5415.2011.03333.x
16. Alessi C, Harker J. A prospective study of acute illness in the nursing home. *Aging* 1998;10(6):479-89.
17. Mor V, Intrator O, Fries B. Changes in hospitalization associated with introducing the Resident Assessment Instrument. *Journal of the American Geriatric Society* 1997;45(8):1002-10.
18. Young Y, Inamdar S, Dichter B. Clinical and nonclinical factors associated with potentially preventable hospitalizations among nursing home residents in New York

For peer review only - http://bmjopen.bmj.com/site/about/guidelines.xhtml

State. *Journal of the American Medical Directors Association* 2011;12(5):364-71. doi: 10.1016/j.jamda.2010.03.006
19. Ouslander J, Perloe M, Givens J. Reducing potentially avoidable hospitalizations of nursing home residents: results of a pilot quality improvement project. *Journal of the American Medical Directors Association* 2009;10(9):644-52. doi: 10.1016/j.jamda.2009.07.001
20. Olsan T. Reducing avoidable Rehospitalisations by Improving Residents' Transitions to the Nursing Home. 2011:B22.
21. Sampson EL, Feast A, Blighe A, et al. Evidence-based intervention to reduce avoidable hospital admissions in care home residents (the Better Health in Residents in Care Homes (BHiRCH) study): protocol for a pilot cluster randomised trial. *BMJ open* 2019;9(5):e026510. doi: 10.1136/bmjopen-2018-026510 [published Online First: 2019/05/28]
22. Kitson AL, Rycroft-Malone J, Harvey G, et al. Evaluating the successful implementation of evidence into practice using the PARiHS framework: theoretical and practical challenges. *ImplementSci* 2008;3:1.
23. Schulz KF, Altman DG, Moher D, et al. CONSORT 2010 statement: updated guidelines for reporting parallel group randomised trials. *BMJ* 2010;340:c332. doi: 10.1136/bmj.c332 [published Online First: 2010/03/25]
24. Dewing J, McCormack B, Titchen A. Practice Development Workbook for Nursing, Health and Social Care Teams. West Sussex: John Wiley and Sons 2014.
25. Rycroft-Malone J, Harvey G, Seers K, et al. An exploration of the factors that influence the implementation of evidence into practice. *JClinNurs* 2004;13(8):913-24.
26. University. FA. Stop and Watch Early Warning Tool 2018 [Available from: http://www.pathway-interact.com/wp-content/uploads/2017/04/148604-Stop-and-Watch-v4_0.pdf accessed 20/02/2020.
27. Mahoney FI, Barthel DW. Functional Evaluation: The Barthel Index. *Md State Med J* 1965;14:61-5. [published Online First: 1965/02/01]
28. Edvardsson D, Fetherstonhaugh D, Nay R, et al. Development and initial testing of the Person-centered Care Assessment Tool (P-CAT). *International psychogeriatrics / IPA* 2010;22(1):101-8. doi: 10.1017/S1041610209990688 [published Online First: 2009/07/28]
29. Tjia J, Mazor KM, Field T, et al. Nurse-physician communication in the long-term care setting: perceived barriers and impact on patient safety. *J Patient Saf* 2009;5(3):145-52. doi: 10.1097/PTS.0b013e3181b53f9b [published Online First: 2009/11/21]
30. Saliba D, Kington R, Buchanan J, et al. Appropriateness of the decision to transfer nursing facility residents to the hospital. *J Am Geriatr Soc* 2000;48(2):154-63. [published Online First: 2000/02/22]
31. Beecham J, Knapp M. Costing psychiatric interventions. In: Thornicroft G, ed. Measuring Mental Helath Needs. London: Gaskell 2001:200-24.
32. Herdman M, Gudex C, Lloyd A, et al. Development and preliminary testing of the new five-level version of EQ-5D (EQ-5D-5L). *Qual Life Res* 2011;20(10):1727-36. doi: 10.1007/s11136-011-9903-x [published Online First: 2011/04/12]
33. Curtis L, Burns A. Unit Costs of Health and Social Care 2017 2017 [updated 2017. Available from: http://www.pssru.ac.uk/project-pages/unit-costs/unit-costs-2017/2018.
34. Joint Formulary Committee. British National Formulary. London: BMJ Group and Pharmaceutical Press 2018.
35. Department of Health. National Schedule of Reference Costs- Year 2016-2017- NHS trusts and NHS foundation trusts: NHS own cost. London, 2016.
36. Devlin NJ, Shah KK, Feng Y, et al. Valuing health-related quality of life: An EQ-5D-5L value set for England. *Health Econ* 2017 doi: 10.1002/hec.3564 [published Online First: 2017/08/24]
37. Devlin NJ, Shah K, Feng Y, et al. Valuing health-related quality of life: an EQ-5D-5L value set for England. Research Paper 16/01. London, 2016.

For peer review only

38. van Hout B, Janssen MF, Feng YS, et al. Interim scoring for the EQ-5D-5L: mapping the EQ-5D-5L to EQ-5D-3L value sets. *Value Health* 2012;15(5):708-15. doi: 10.1016/j.jval.2012.02.008 [published Online First: 2012/08/08]
39. National Institute for Clinical Excellence. Position statement on use of the EQ-5D-5L valuation set, 2017.
40. Briggs AH, Gray AM. Handling uncertainty when performing economic evaluation of healthcare interventions. *Health technology assessment* 1999;3(2):1-134. [published Online First: 1999/08/17]
41. Kane RL, Huckfeldt P, Tappen R, et al. Effects of an Intervention to Reduce Hospitalizations From Nursing Homes: A Randomized Implementation Trial of the INTERACT Program. *JAMA Intern Med* 2017;177(9):1257-64. doi: 10.1001/jamainternmed.2017.2657 [published Online First: 2017/07/04]
42. Gordon AL, Franklin M, Bradshaw L, et al. Health status of UK care home residents: a cohort study. *Age Ageing* 2014;43(1):97-103. doi: 10.1093/ageing/aft077
43. Saliba D, Kington R, Buchanan J, et al. Appropriateness of the decision to transfer nursing facility residents to the hospital. *Journal of the American Geriatrics Society* 2000;2000/02/22:154-63.
44. Romoren M, Gjelstad S, Lindbaek M. A structured training program for health workers in intravenous treatment with fluids and antibiotics in nursing homes: A modified stepped-wedge cluster-randomised trial to reduce hospital admissions. *PLoS One* 2017;12(9):e0182619. doi: 10.1371/journal.pone.0182619 [published Online First: 2017/09/08]
45. Van den Block L, Honinx E, Pivodic L, et al. Evaluation of a Palliative Care Program for Nursing Homes in 7 Countries: The PACE Cluster-Randomized Clinical Trial. *JAMA Intern Med* 2019:1-10. doi: 10.1001/jamainternmed.2019.5349 [published Online First: 2019/11/12]
46. Ballard C, Corbett A, Orrell M, et al. Impact of person-centred care training and person-centred activities on quality of life, agitation, and antipsychotic use in people with dementia living in nursing homes: A cluster-randomised controlled trial. *PLoS Med* 2018;15(2):e1002500. doi: 10.1371/journal.pmed.1002500 [published Online First: 2018/02/07]
47. Gordon AL, Goodman C, Davies SL, et al. Optimal healthcare delivery to care homes in the UK: a realist evaluation of what supports effective working to improve healthcare outcomes. *Age Ageing* 2018;47(4):595-603. doi: 10.1093/ageing/afx195 [published Online First: 2018/01/10]
48. Sherlaw-Johnson C, Crump H, Curry N, et al. Transforming health care in nursing homes: An evaluation of a dedicated primary care service in outer east London. London, 2018.
49. Morgan B, Hejdenberg J, Hinrichs-Krapels S, et al. Do feasibility studies contribute to, or avoid, waste in research? *PLoS One* 2018;13(4):e0195951. doi: 10.1371/journal.pone.0195951 [published Online First: 2018/04/24]

Figure title and legend

Figure 1 title: Resident recruitment flowchart

For peer review only - http://bmjopen.bmj.com/site/about/guidelines.xhtml

For peer review only

For peer review only - http://bmjopen.bmj.com/site/about/guidelines.xhtml

Figure 1. Resident recruitment flowchart

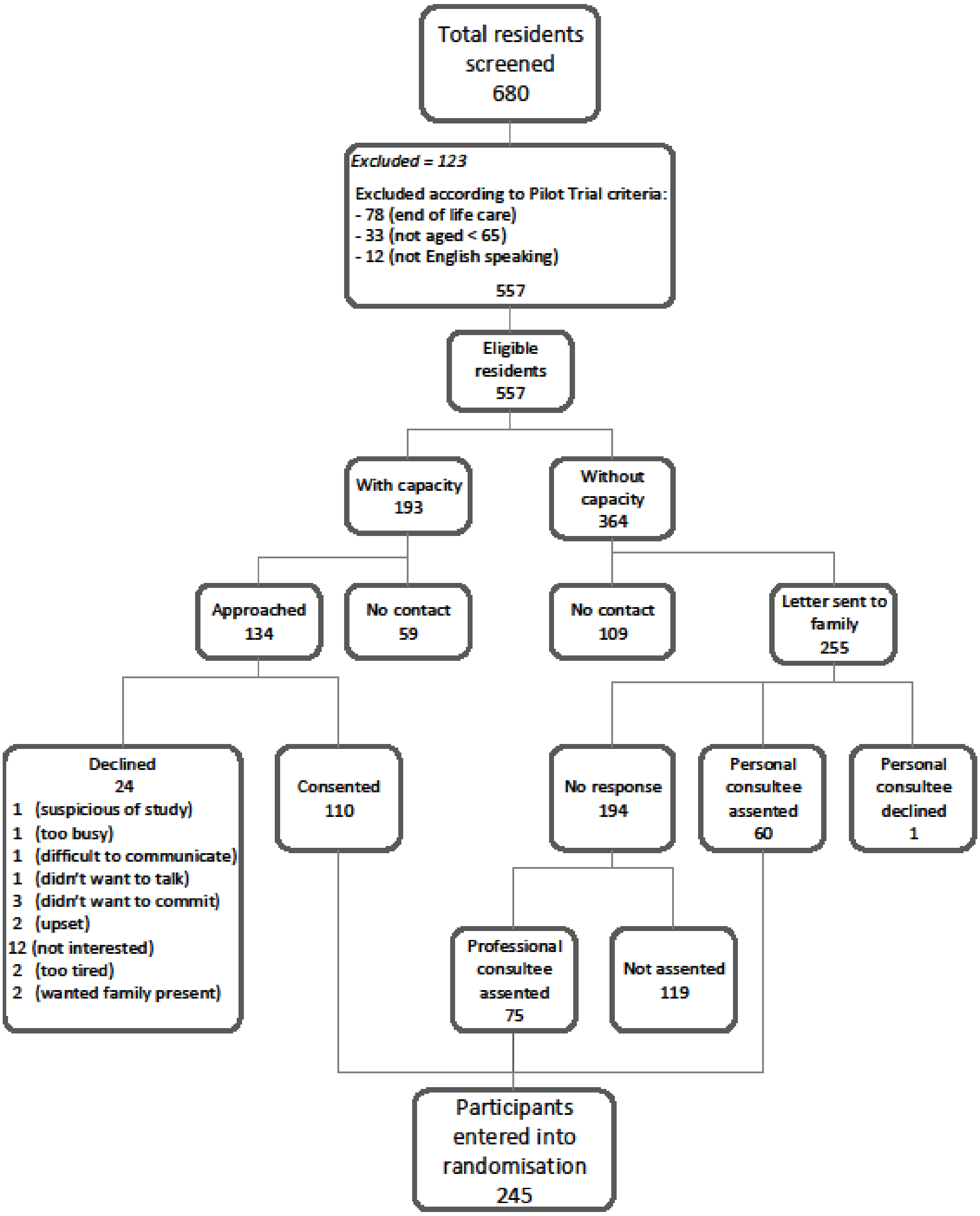

Better Health in Residents of Care Homes with Nursing - BHiRCH-NH study

Appendix 1: Pre-intervention characteristics of participating nursing homes

Characteristic	All care homes	TAU	BHiRCH-NH
	n/N or median (% or IQR)		
**Residents and beds**			
Beds available to new residents	1 (1,3)	2 (0, 2)	1 (1, 3)
Resident places	60 (36, 71)	67 (47, 84)	49 (28, 64)
Number of residents present in home	50 (34, 68)	50 (47, 83)	46 (25, 63)
Number of residents with dementia	20 (15, 33)	33 (15, 44)	20 (13, 23)
Number of residents in hospital	0 (0, 1)	1 (0, 2)	0 (0, 1)
**Medical attendances**			
Number hospital admissions	3 (2, 5)	4 (2, 7)	3 (2, 4)
Number of ambulances called	3 (2, 6)	4 (2, 9)	3 (2, 6)
Unscheduled (out of hours) GP visits or telephone contacts	1 (1, 3)	2 (1, 3)	1 (1, 3)
Accident and Emergency attendances	3 (2, 5)	3 (1, 4)	3 (2, 6)
**Staffing**			
Qualified nursing staff rostered on during the day	3 (2, 5)	3 (2, 5)	3 (2, 5)
Care staff rostered on during the day	11 (9, 13)	11 (9, 14)	12 (6, 13)
Qualified nursing staff rostered on during the night	2 (1, 3)	2 (1, 3)	2 (1, 3)
Care staff rostered on during the night	3 (3, 7)	3 (3, 7)	4 (2, 7)
Number of agency/ bank staff in 24 hour period	1 (0, 3)	1 (0, 3)	1 (0, 3)
Number of permanent registered nursing staff (including those on sick/carer/compassionate leave)	10 (8, 13)	11 (10, 13)	8 (7, 15)
Number of permanent other care staff (including those on sick/carer/compassionate leave)	26 (24, 57)	26 (24, 70)	33 (23, 57)
Number of registered nursing staff from those above on sick/carer/ compassionate leave	0 (0, 1)	0 (0, 1)	0 (0, 1)
Number of other care staff from those above on sick/carer/ compassionate leave	0 (0, 2)	1 (0, 6)	0 (0, 2)
**Nursing home**			
Privately managed	12/13 (92)	6/7 (86)	6/6 (100)
Local Authority managed	1/13 (8)	1/7 (14)	0/6 (0)
Nursing	6/13 (46)	4/7 (57)	2/6 (33)
Nursing and personal care	7/13 (54)	3/7 (43)	4/6 (67)
Dementia registered	8/11 (73)	5/6 (83)	3/5 (60)
Dementia specialist	2/12 (17)	2/6 (33)	0/6 (0)
**Regular nursing home attendance from**			
Physiotherapist	7/12* (58)	4/7 (57)	3/5 (60)
Geriatrician	3/12 (25)	2/7 (29)	1/5 (20)
District Nurse	5/13 (38)	3/7 (43)	2/6 (33)
Tissue viability nurses	7/12 (58)	3/7 (43)	4/5 (80)
Dietitian	9/13 (69)	4/7 (57)	5/6 (83)
Speech and language therapist	8/13 (62)	4/7 (57)	4/6 (67)
Optician	11/13 (85)	5/7 (71)	6/6 (100)
Ophthalmologist	4/13 (31)	1/7 (14)	3/6 (50)
Chiropodist	13/13 (100)	7/7 (100)	6/6 (100)
Occupational therapist	7/13 (54)	3/7 (43)	4/6 (67)
Dentist	8/12 (67)	5/7 (71)	3/5 (60)
Audiologist	2/12 (17)	1/7 (14)	1/5 (20)
Dedicated GP for all residents	13/13 (100)	7/7 (100)	6/6 (100)

***for some variables N=12 where this data was not available for a particular home**

For peer review only

Appendix 2. Family carer recruitment and retention

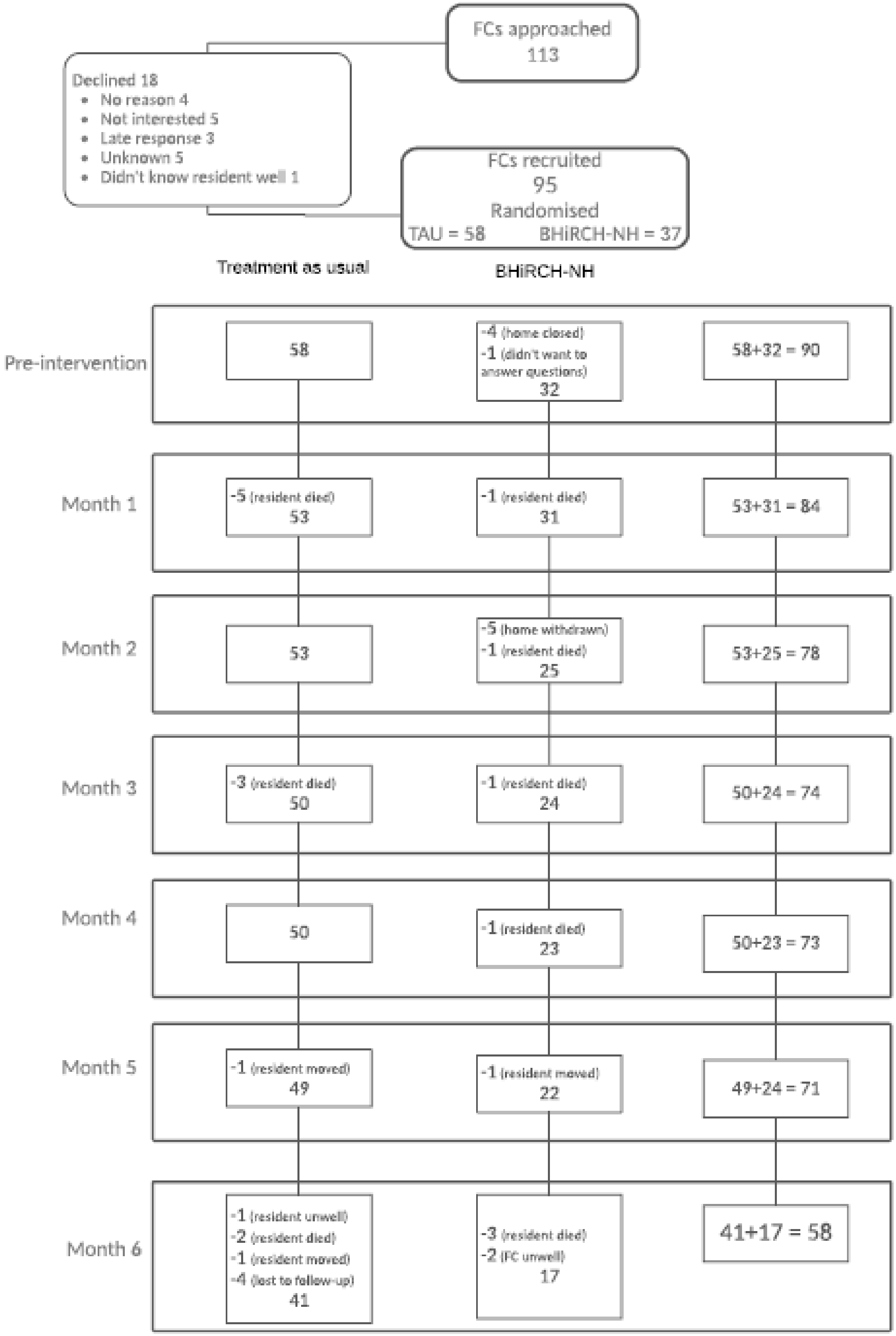

Appendix 3. Staff recruitment and retention flowchart

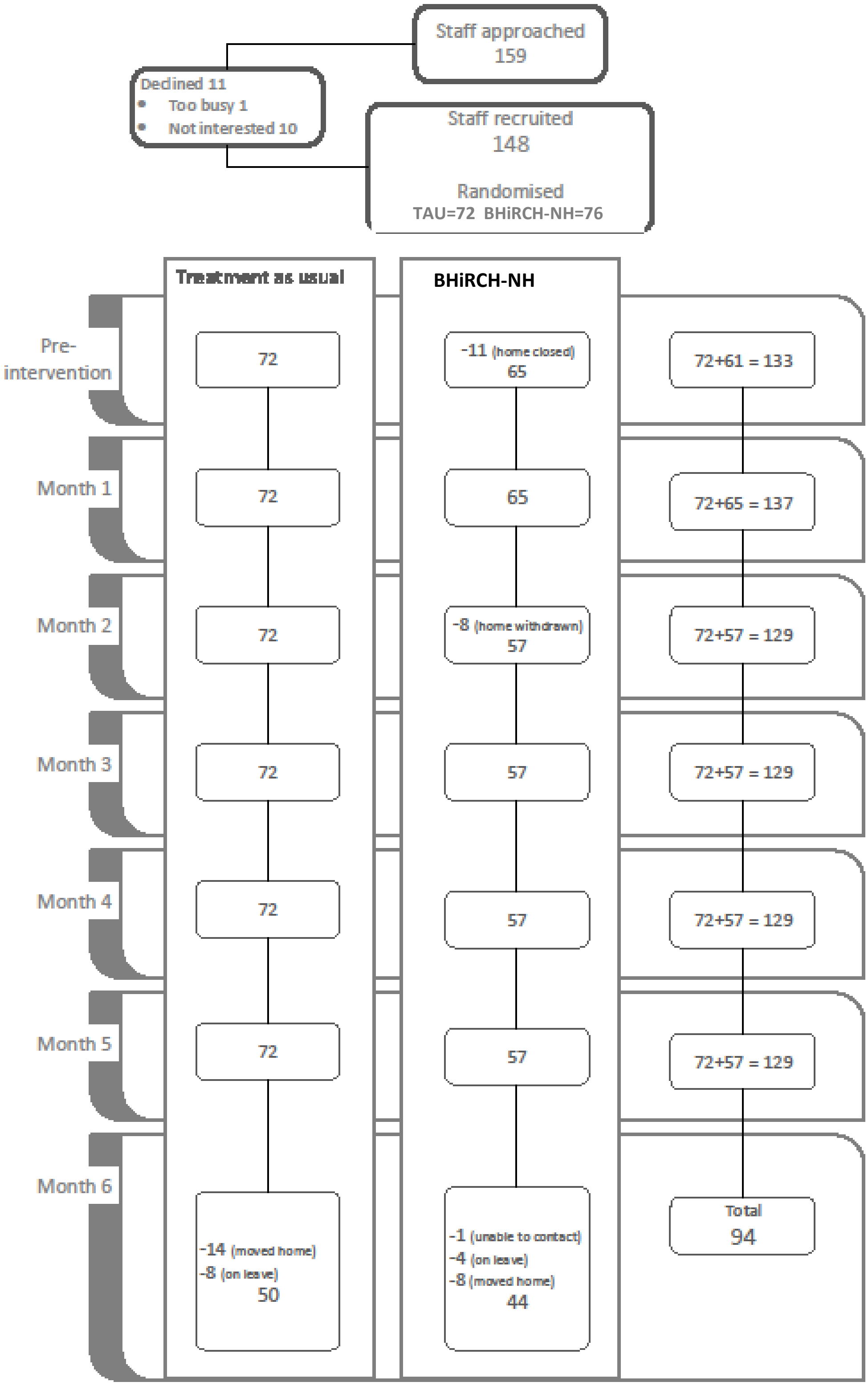

Appendix 4: Staff participant characteristics pre-intervention

Characteristic	Cohort	TAU	Intervention
	n/N or median (% or IQR)		
**Demographics**	N=130	N=70	N=60
Male	16 (12)	9 (13)	7 (12)
Age	42 (30, 53)	42(32, 53)	39 (27, 50)
**Ethnicity**	N=129	N=69	N=60
White	76 (59)	32 (46)	44 (73)
Black	29 (22)	21 (30)	8 (13)
Asian	16 (12)	9 (13)	7 (12)
Other	8 (16)	7 (10)	1 (2)
**Marital status**	N=129	N=69	N=60
Married or cohabiting	71 (55)	40 (58)	31 (52)
Single	47 (36)	22 (32)	25 (42)
Divorced or widowed	11 (9)	7 (10)	4 (7)
**Education**	N=128	N=69	N=59
Completed years of education	11(11, 12)	12 (11, 12)	11 (11, 12)
No qualifications or GCSE or equivalent	16 (13)	9 (13)	7 (12)
A Level/NVQ/ HNC/ HND or equivalent	46 (36)	18 (26)	28 (47)
Degree or higher degree	49 (38)	28 (41)	21 (36)
Other qualification	17 (13)	14 (20)	3 (5)
**Type of staff**	N=125	N=67	N=58
Registered Nurse	24 (19)	9 (13)	7 (12)
Manager/ Matron/ Charge Nurse	12 (10)	8 (12)	4 (7)
Staff Nurse	2 (2)	5 (7)	3 (5)
Agency Nurse	87 (70)	1 (1)	1 (2)
Not a nurse		44 (66)	43 (74)
**Nurses only**	N=40	N=24	N=16
Works days	28 (70)	16 (67)	12 (75)
Works evenings	2 (5)	1 (4)	1 (6)
Works nights	9 (23)	4 (17)	5 (31)
Works mornings/early 12-hour shifts	12 (30)	5 (21)	7 (44)
Works afternoons/late 12-hour shifts	3 (8)	0 (0)	3 (19)
Works weekends	3 (8)	1 (4)	2 (13)
**Number of hours/week working in this nursing home**	N=40	N=24	N=16
Less than 8	1 (3)	0 (0)	1 (6)
8 to 12	0 (0)	0 (0)	0 (0)
13 to 40	13 (33)	8 (33)	5 (31)
More than 40	26 (65)	16 (67)	10 (63)
**How long have you worked at this nursing home?**	N=40	N=24	N=16
Less than 2 months	1 (3)	1 (4)	0
2 to 6 months	4 (10)	3 (13)	1 (6)
7 to 11 months	7 (18)	4 (17)	3 (19)
1 to 5 years	17 (43)	7 (29)	10 (63)
6 to 10 years	6 (15)	6 (25)	0
More than 10 years	5 (13)	3 (13)	2 (13)
**How long have you worked in nursing homes?**	N=39	N=24	N=15
7 to 11 months	2 (5)	1 (4)	1 (7)
1 to 5 years	14 (36)	6 (25)	8 (53)
6 to 10 years	7 (18)	7 (29)	0
More than 10 years	16 (41)	10 (42)	6 (40)
**How long have you been a registered nurse?**	N=40	N=24	N=16
1 to 5 years	8 (20)	3 (13)	5 (31)
6 to 10 years	10 (25)	7 (29)	3 (19)
More than 10 years	22 (55)	14 (58)	8 (50)

For peer review only

Better Health in Residents of Care Homes with Nursing - BHiRCH-NH study

**English is first language**	20 (50)	12 (50)	8 (50)
**PCAT Score**	49 (I46, 53)	50 (47,54)	48 (44,51)

For peer review only

Better Health in Residents of Care Homes with Nursing - BHiRCH-NH study

Appendix 5: Nurse – General Practitioner Communication Tool scores

		Pre-intervention		6 month follow up	
	**Cohort**	**TAU**	**BHiRCH-NH n/N (%)**	**TAU**	**BHiRCH-NH**
Have difficulty understanding what GP means due to medical jargon	2/40 (5)	2/24 (8)	0/16 (0)	0/14 (0)	0/7 (0)
A GP's language or accent makes it hard for you to understand what they are saying	2/40 (5)	1/24 (4)	1/16 (16)	0/15 (0)	0/7 (0)
A GP has difficulty understanding what you are saying due to your language or accent	2/40 (5)	2/24 (8)	0/16 (0)	0/15 (0)	0/7 (0)
GPs interrupt you before you have finished reporting on a resident	4/40 (10)	2/24 (8)	2/16 (13)	1/14 (7)	0/7 0)
GPs consider nurses' views when making decisions about residents	29/39 (10)	18/23 (78)	11/16 (69)	12/15 (80)	5/7 (71)
GPs are rude to you when you call them about a resident	2/39 (5)	2/23 (9)	0/16 (0)	1/15 (7)	0/7 (0)
You feel respected after an interaction with a GP?	29/40 (73)	19/24 (79)	10/16 (63)	12/15 (80)	4/7 (57)
You feel frustrated after an interaction with a GP?	4/40 (10)	3/24 (13)	1/16 (6)	1/15 (7)	0/7 (0)
Difficulty reaching the GP	9/40 (23)	5/24 (21)	4/16 (25)	2/15 (13)	2/7 (29)
Uncertainty about what to tell the GP	1/38 (3)	1/23 (4)	0/15 (0)	0/15 (0)	0/7 (0)
Feeling that the GP doesn't want to deal with the problem	3/40 (8)	1/24 (4)	2/16 (13)	1/15 (7)	1/7 (14)
Finding time to make the call	11/40 (28)	8/24 (33)	3/16 (19)	3/15 (20)	2/7 (29)
Finding a quiet place to make the call	10/40 (25)	7/24 (29)	3/16 (19)	4/14 (29)	0/7 (0)
Anticipating that the GP will be rude or unpleasant	1/40 (3)	0/24 (0)	1/16 (6)	1/15 (7)	0/7 (0)
Feeling hurried by the GP	7/40 (18)	4/24 (17)	3/16 (19)	1/15 (7)	0/7 (0)
Feeling that I am bothering the GP	4/39 (10)	2/24 (8)	2/15 (13)	2/15 (13)	0/7 (0)
Worrying that the GP may order something inappropriate or unnecessary	4/40 (10)	2/24 (8)	2/16 (13)	1/15 (7)	0/7 (0)
Feeling that I don't have enough time to say everything that I need to say	3/39 (8)	1/24 (4)	2/15 (13)	1/15 (7)	0/7 (0)
Nurses get good training in this care home	28/39 (72)	16/24 (67)	12/15 (80)	14/15 (93)	5/7 (71)
Nurses in this care home are willing to try new protocols	33/39 (85)	20/24 (83)	13/15 (87)	11/15 (73)	5/7 (71)
Nurses in this care home sometimes have to ignore protocols to get everything done	6/39 (15)	4/24 (17)	2/15 (13)	3/15 (20)	1/7 (14)
When this care home makes changes to improve resident care, they follow up to see if the changes worked	31/39 (79)	18/24 (75)	13/15 (87)	15/15 (100)	6/7 (86)
It is hard to make changes to improve resident care in this care home	4/39 (10)	1/24 (4)	3/15 (20)	2/15 (13)	0/7 (0)
This care home often wants nurses to follow protocols that don't really help residents	3/39 (8)	1/24 (4)	2/15 (13)	2/15 (13)	0/7 (0)
New protocols often make it harder for nurses to do their job	4/39 (10)	2/24 (8)	2/15 (13)	4/15 (27)	0/7 (0)
The communication between nurses and GPs in this care home is open	34/40 (85)	20/24 (83)	14/16 (88)	13/15 (87)	7/7 (100)
Feel comfortable communicating with GPs?	36/40 (90)	23/24 (96)	13/16 (81)	15/15 (100)	7/7 (100)
Feel comfortable communicating with nurse practitioners?	34/40 (85)	23/24 (96)	11/16 (69)	14/15 (93)	6/6 (100)

For peer review only

Better Health in Residents of Care Homes with Nursing - BHiRCH-NH study

Appendix 6: Nurse self- assessed core competencies

Core competencies	Pre-intervention Cohort	TAU	BHiRCH-NH	6 month follow up TAU	BHiRCH-NH
			n/N (%)		
**I know how acute episodes of chronic heart failure may manifest in older people**					
Disagree	0/34 (0)	0/21 (0)	0/13 (0)	0/15 (0)	0/7 (0)
Neither agree nor disagree	2/34 (6)	0/21 (0)	2/13 (15)	1/15 (7)	0/7 (0)
Agree	18/34 (53)	12/21 (57)	6/13 (46)	7/15 (47)	4/7 (57)
Completely agree	14/34 (41)	9/21 (43)	5/13 (38)	7/15 (47)	3/7 (43)
**I know how respiratory infections may manifest in older people**					
Disagree	0/34 (0)	0/21 (0)	0/13 (0)	0/15 (0)	0/7 (0)
Neither agree nor disagree	0/34 (0)	0/21 (0)	0/13 (0)	0/15 (0)	0/7 (0)
Agree	13/34 (38)	7/21 (33)	6/13 (46)	6/15 (40)	3/7 (43)
Completely agree	21/34 (62)	14/21 (67)	7/13 (54)	9/15 (60)	4/7 (57)
**I know how dehydration may manifest in older people**					
Disagree	0/34 (0)	0/21(0)	0/13 (0)	0/15 (0)	0/7 (0)
Neither agree nor disagree	0/34 (0)	0/21 (0)	0/13 (0)	0/15(0)	0/7 V
Agree	14/34 (41)	7/21 (33)	7/13 (54)	5/15 (33)	1/7 (14)
Completely agree	20/34 (59)	14/21 (67)	6/13 (46)	10/15 (67)	6/7 (86)
**I know how urinary tract infections may manifest in older people**					
Disagree	0/34 (0)	0/21 (0)	0/13 (0)	0/15 (0)	0/7 (0)
Neither agree nor disagree	0/34 (0)	0/21 (0)	0/13 (0)	0/15 (0)	0/7 (0)
Agree	15/3 4 (44)	9/21 (43)	6/13 (46)	5/15	1/7 (14)
Completely agree	19/34 (56)	12/21 (57)	7/13 (54)	10/15 (67)	6/7 (86)
**I am able to identify changes in physiology or behaviour as compared to baseline or normal for that resident**					
Disagree	0/34 (0)	0/21 (0)	0/13 (0)	0/15 (0)	0/7 (0)
Neither agree nor disagree	0/34 (0)	0/21 (0)	0/13 (0)	0/15 (0)	0/7 (0)
Agree	14/34 (41)	8/21(38)	6/13 (46)	5/15 (33)	0/7 (0)
Completely agree	20/34 (59)	13/21 (62)	7/13 (54)	10/15 (67)	7/7 (100)
**I am able to identify changes in physiology or behaviour for people who cannot communicate verbally**					
Disagree	0/34 (0)	0/21	0/13 (0)	0/15 (0)	0/7 (0)
Neither agree nor disagree	1/34 (3)	0/21(0)	1/13 (8)	1/15 (7)	0/7 (0)
Agree	17/34 (50)	11/21 (52)	6/13 (46)	6/15 (40)	2/7 (29)
Completely agree	16/34 (47)	10/21 (48)	6/13 (46)	8/15 (53)	5/7 (71)
**I know each resident's existing medical conditions**					
Disagree	1/34 (3)	0/21 (0)	1/13 (8)	0/15 (0)	1/7 (14)
Neither agree nor disagree	2/34 (6)	1/21 (5)	1/13 (8)	1/15 (7)	0/7 (0)
Agree	22/34 (65)	15/21 (71)	7/13 (54)	8/15 (53)	6/7 (86)
Completely agree	9/34 (26)	5/21 (24)	4/13 (31)	6/15 (40)	0/7 (0)
**I know each resident's care plans with respect to these medical conditions**					
Disagree	1/34 (3)	0/21 (0)	1/13 (8)	0/15 (0)	0/7(0)
Neither agree nor disagree	2/34 (6)	1/21 (5)	1/13 (8)	1/15 (7)	1/7 (14)
Agree	17/34 (50	10/21 (48)	7/13 (54)	8/15 (53)	5/7 (71)

For peer review only

Completely agree	14/34 (41)	10/21 (48)	4/13 (31)	6/15 (40)	1/7 (14)
**I know what is normal (i.e. their baseline) for each resident in terms of physiology, abilities and behaviour**					
Disagree	0/34 (0)	0/21 (0)	0/13 (0)	0/15 (0)	0/7 (0)
Neither agree nor disagree	1/34 (3)	1/21 (5)	0/13 (0)	2/15 (13)	0/7(0)
Agree	19/34 (56)	11/21 (52)	8/13 (62)	7/15 (47)	6/7 (86)
Completely agree	14/34 (41)	9/21 (43)	5/13 (38)	6/15 (40)	1/7 (14)
**I am able to carry out immediate interventions (eg adjusting fluid levels)**					
Disagree	1/33 (3)	1/21 (5)	0/12 (0)	0/15 (0)	0/7 (0)
Neither agree nor disagree	0/33 (0)	0/21 (0)	0/12 (0)	0/15 (0)	1/7 (14)
Agree	21/33 (64)	10/21 (48)	11/12 (92)	6/15 (40)	6/7(86)
Completely agree	11/33 (33)	10/21 (48)	1/12 (8)	9/15 (60)	0/7 (0)
**I am able to recognise verbal and nonverbal signs of acute deterioration**					
Disagree	0/34 (0)	0/21 (0)	0/13 (0)	0/15 (0)	0/7 (0)
Neither agree nor disagree	0/34 (0)	0/21 (0)	0/13 (0)	1/15 (7)	0/7 (0)
Agree	19/34 (56)	10/21 (48)	9/13 (69)	6/15 (40)	4/7 (57)
Completely agree	15/34 (44)	11/21 (52)	4/13 (31)	8/15 (53)	3/7 (43)
**I am able to carry out bedside observations (vital signs etc)**					
Disagree	0/34 (0)	0/21 (0)	0/13 (0)	0/15 (0)	0/7 (0)
Neither agree nor disagree	0/34 (0)	0/21 (0)	0/13 (0)	0/15 (0)	0/7 (0)
Agree	11/34 (32)	6/21 (29)	5/13 (38)	5/15 (33)	4/7 (57)
Completely agree	23/34 (68)	15/21 (71)	8/13 (62)	10/15(67)	3/7 (43)
**I am able to utilise basic clinical skills (use of medication etc)**					
Disagree	0/34 (0)	0/21 (0)	0/13 (0)	0/15 (0)	0/7 (0)
Neither agree nor disagree	0/34 (0)	0/21 (0)	0/13 (0)	0/15 (0)	0/7 (0)
Agree	11/34 (32)	5/21 (24)	6/13	5/15	3/7
Completely agree	23/34 (68)	16/21 (76)	7/13 (54)	10/15 (67)	4/7 (57)
I **know when to seek additional support (eg. asking a nurse, calling a GP or ambulance)**					
Disagree	0/34 (0)	0/21 (0)	0/13 (0)	0/15 (0)	0/7 (0)
Neither agree nor disagree	0/34 (0)	0/21 (0)	0/13 (0)	0/15 (0)	0/7 (0)
Agree	10/34 (29)	4/21 (19)	6/13 (46)	4/15 (27)	2/7 (29)
Completely agree	24/34 (71)	17/21 (81)	7/13 (54)	11/15 (73)	5/7 (71)
**I am able to set and monitor staff work to ensure quality of health care is provided**					
Disagree	1/34 (3)	1/21 (5)	0/13 (0)	1/15 (7)	0/7(0)
Neither agree nor disagree	0/34 (0)	0/21 V	0/13 V	0/15 (0)	0/7 (0)
Agree	14/34 (41)	5/21 (24)	9/13 (69)	4/15 (27)	3/7 (43)
Completely agree	19/34 (56)	15/21 (71)	4/13 (31)	10/15 (67)	4/7 (57)
**I am able to encourage or support other staff in their care for acute deterioration in resident's health**					
Disagree	0/34 (0)	0/21 V	0/13 (0)	0/15 (0)	0/7 (0)
Neither agree nor disagree	2/34 (6)	2/21 (10)	0/13 (0)	0/15 (0)	0/7 (0)
Agree	14/34 (41)	5/21 (24)	9/13 (69)	6/15 (40)	3/ (43)7
Completely agree	18/34 (53)	14/21 (67)	4/13 (31)	9/15 (60)	4/7 (57)
**I am able to negotiate and plan a course of action with other staff**					
Disagree	0/34 (0)	0/21 (0)	0/13 (0)	0/15 (0)	0/7 (0)
Neither agree nor disagree	4/34 (12)	3/21 (14)	1/13 (8)	1/15 (7)	0/7 (0)
Agree	13/34 (38)	6/21 (29)	7/13 (54)	5/15 (33)	5/7 (71)

For peer review only

Completely agree	17/34 (50)	12/21 (57)	5/13 (38)	9/15 (60)	2/7 (29)
**I am able to record a plan of action, e.g. draft and update care plans**					
Disagree	0/34 (0)	0/21 (0)	0/13 (0)	0/15 (0)	0/ (0)7
Neither agree nor disagree	0/34 (0)	0/21 (0)	0/13 (0)	1/15 (7)	0/7 (0)
Agree	16/34 (47)	10/21 (48)	6/13 (46)	4/15 (27)	3/7 (43)
Completely agree	18/34 (53)	11/21 (52)	7/13 (54)	10/15 (67)	4/7 (57)
**I am able to ensure acute changes in a resident's health status are communicated to staff in written or oral form**					
Disagree	0/34 (0)	0/21 (0)	0/13 (0)	0/15 (0)	0/7 (0)
Neither agree nor disagree	0/34 (0)	0/21 (0)	0/13 (0)	1/15 (7)	0/7(0)
Agree	14/34 (41)	7/21 (33)	7/13 (54)	5/15 (33)	3/7 (43)
Completely agree	20/34 (59)	14/21 (67)	6/13 (46)	9/15 (60)	4/7 (57)
**I am able to ensure effectiveness of treatment for acute changes in a resident's health status are communicated to staff and carers in written or oral form**					
Disagree	0/34 (0)	0/21 (0)	0/13 (0)	0/15 (0)	0/7 (0)
Neither agree nor disagree	2/34 (6)	1/21 (5)	1/13 (8)	1/15 (7)	0/7 (0)
Agree	12/34 (35)	6/21 (29)	6/13 (46)	6/15 (40)	2/7 (29)
Completely agree	20/34 (59)	14/21 (67)	6/13 (46)	8/15 (53)	5/7 (71)
**I am able to convey information about changes in health accurately and sensitively to carers**					
Disagree	0/34 (0)	0/21 (0)	0/13 (0)	0/15 (0)	0/6 (0)
Neither agree nor disagree	1/34 (3)	0/21 (0)	1/13 (8)	1/15 (7)	0/6 (0)
Agree	16/34 (47)	10/21 (48)	6/13 (46)	6/15 (40)	2/6 (33)
Completely agree	17/34 (50)	11/21 (52)	6/13 (46)	8/15 (53)	4/6 (67)
**I am able to adapt assessment and communication for people with dementia or communication difficulties**					
Disagree	1/34 (3)	1/21 (5)	0/13 (0)	0/15 (0)	0/7 (0)
Neither agree nor disagree	1/34 (3)	1/21 (5)	0/13 (0)	1/15 (7)	0/7 (0)
Agree	17/34 (50)	11/21 (52)	6/13 (460	6/15 (40)	3/7 (43)
Completely agree	15/34 (44)	8/21 (38)	7/13 (54)	8/15 (53)	4/7 (57)
**I am able to communicate the nature of change in a resident's health status**					
Disagree	0/34 (0)	0/21 (0)	0/13 (0)	0/15 (0)	0/7 (0)
Neither agree nor disagree	0/34 (0)	0/21 (0)	0/13 (0)	1/15 (7)	0/7 (0)
Agree	17/34 (50)	12/21 (57)	5/13 (38)	5/15 (33)	3/7 (43)
Completely agree	17/34 (50)	9/21 (43)	8/13 (62)	9/15 (60)	4/7 (57)

For peer review only

Appendix 7. Serious adverse events by randomised group

SAE	TAU N=55* n/N	Intervention N=41* n/N
**Death**	17 (31)	14 (34)
**Requires hospitalisation or prolonged hospital stay**	37 (67)	25 (61)
**Other medically significant**	1 (2)	2 (5)
**Not caused by the intervention**	55 (100)	41 (100)
**Expected side effect or outcome of the intervention**	55 (100)	39 (95)
**Not expected**	0 (0)	2 (5)
**Not related to the intervention**	55 (100)	41 (100)

*(denominator is SAE not residents)

For peer review only

Appendix 8. Resident retention flowchart

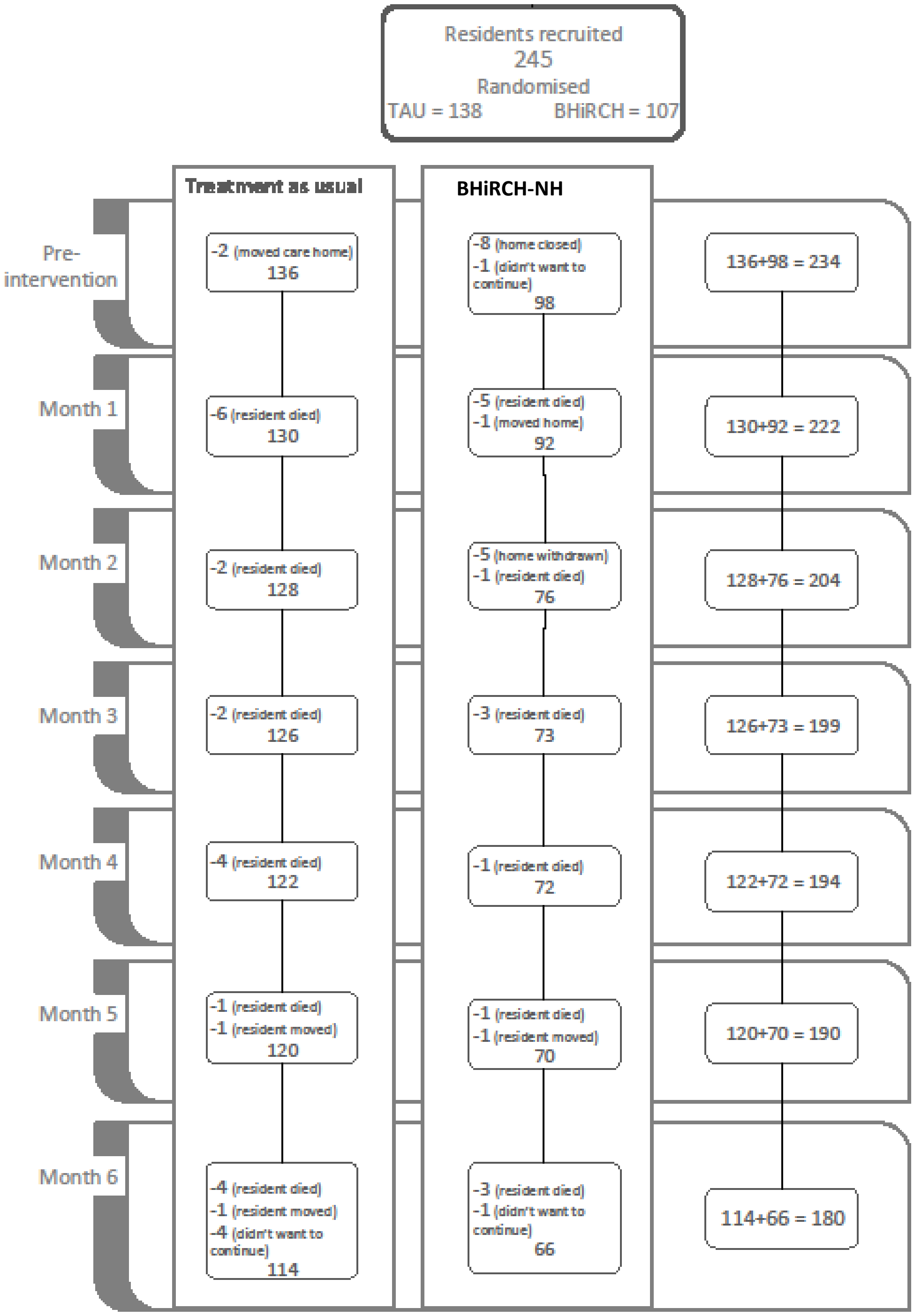

Appendix 9. Mean healthcare resource use ± standard deviation (SD) and cost (unit costs at 2016/2017 values) per resident over the 6 months, complete data

Type of resource use (unit)	TAU	BHiRCH-NH	Difference (BHiRCH-NH vs TAU)		TAU	BHiRCH-NH	Incremental difference (BHiRCH-NH vs TAU)
	Mean ± SD	Mean ± SD	Mean	p-value	Mean cost	Mean cost	Mean cost (95% CI)
**Primary care, community health or emergency services (visits)**	8.3 ± 6.1 *(n=114)*	9.6 ± 7.6 *(n=71)*	1.3	0.20	£491.3	£694.9	£203.6 (-£67.4 to £474.6)
**Outpatient appointments (visits)**	0.4 ± 1.0 *(n=114)*	0.7 ± 1.5 *(n=68)*	0.3	0.11	£22.6	£49.9	£27.4 (£1.2 to £53.5)
**Inpatient services (admissions)**	0.2 ± 0.5 *(n=114)*	0.2 ± 0.6 *(n=68)*	0.001	0.99	£356.9	£426.6	£69.6 (-£333.8 to £472.9)
**Ambulance service (calls)**	0.2 ± 0.6 *(n=114)*	0.2 ± 0.5 *(n=68)*	0.04	0.61	£1.3	£1.6	£0.3 (-£0.9 to £1.4)
**Accident and Emergency (attendances)**	0.1 ± 0.5 *(n=114)*	0.3 ± 0.6 *(n=68)*	0.1	0.21	£23.1	£38.7	£15.6 (-£8.9 to £40.1)
**Prescriptions (packages)**	54.1 ± 23.8 *(n=114)*	60.5 ± 26.6 *(n=68)*	6.33	0.10	£325.7	£381.6	£55.9 (-£43.8 to £155.6)
**Length of stay in hospital (days)**	0.8 ± 3.9 *(n=114)*	0.9 ± 3.9 *(n=68)*	0.2	0.76			

For peer review only

Appendix 10. Mean utility values and QALYs per resident (based on resident self-completed EQ-5D-5L questionnaire)

Utility values	TAU		BHiRCH-NH		Incremental difference	
	Mean	(95%CI)	Mean	(95%CI)	Mean	p-value
**Complete cases**						
Baseline	0.610 *(n=69)*	0.540 to 0.680	0.487 *(n=58)*	0.403 to 0.570	-0.123	0.02
6 months	0.685 *(n=59)*	0.610 to 0.759	0.614 *(n=32)*	0.501 to 0.727	-0.071	0.28
**QALYs**	0.337 *(n=46)*	0.297 to 0.377	0.259 *(n=26)*	0.196 to 0.321	-0.078	0.03
**With imputations**						
Baseline	0.617	0.607 to 0.628	0.504	0.491 to 0.518	-0.113	<0.001
6 months	0.652	0.641 to 0.662	0.649	0.637 to 0.661	-0.003	0.73
**QALYs**	0.317	0.312 to 0.322	0.289	0.283 to 0.294	-0.029	<0.001

For peer review only

Appendix 11. Mean cost ± standard deviation (SD) of BHiRCH-NH and TAU of resource use per resident over 6 months

Type of costs	TAU	BHiRCH-NH	BHiRCH-NH vs. TAU
**BHiRCH-NH intervention costs per resident**			
Training	£0 ± £0	£39 ± £28	£39
Materials used in training	£0 ± £0	£0.20 ± £0.10	£0.20
Delivery of the intervention	£0 ± £0	£34 ± £32	£34
**Total costs (95%CI) per resident of healthcare resource use over 6 months**			
Complete cases	£1,250 (£900 to £1,599) *(n=105)*	£1,560 (£912 to £2,209) *(n=66)*	£310 (-£361 to £981) *(n=171)*
With imputation	£1,233 (£1,171 to £1,295)	£1,458 (£1,351 to £1,566)	£225 (£108 to £342)
**Total costs (95%CI) per resident of BHiRCH-NH intervention cost + healthcare resource use over 6 months**			
Complete cases	£1,250 (£900 to £1,599) *(n=105)*	£1,640 (£989 to £2,291) *(n=66)*	£390 (-£283 to £1,063) *(n=171)*
With imputation	£1,233 (£1,171 to £1,295)	£1,532 (£1,424 to £1,640)	£299 (£182 to £417)

For peer review only

Appendix 12. Cost-effectiveness acceptability curve showing the probability that BHiRCH-NH intervention is cost-effective versus TAU at different values of the WTP for a QALY, N=237

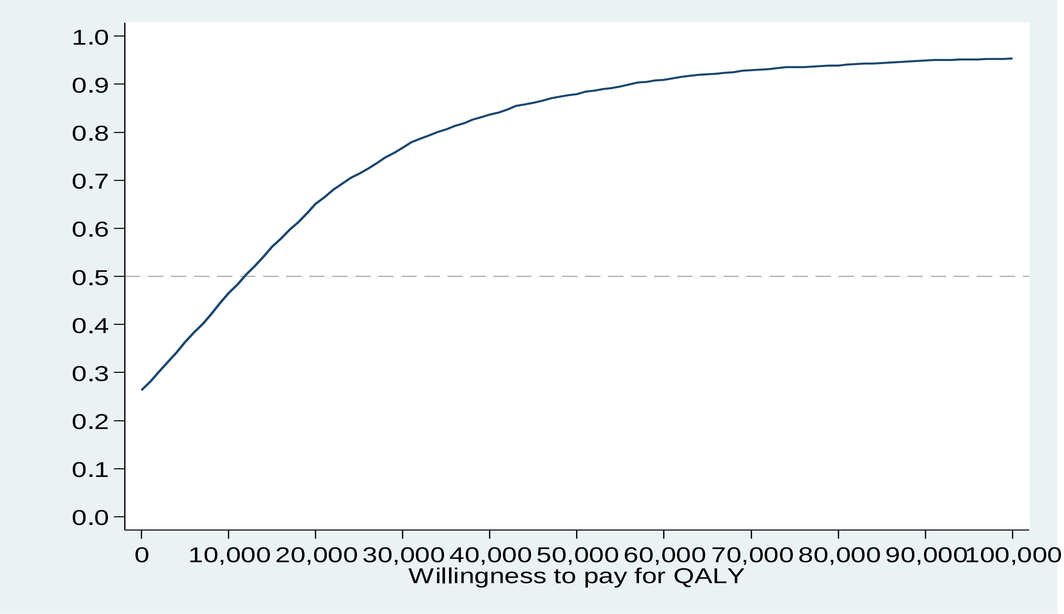

Figure 5 legend: Abbreviations: BHiRCH-NH-Better Health in Residents of Care Homes with Nursing study, QALY- Quality Adjusted Life year, TAU-Treatment as usual, WTP-Willingness to Pay

review only

For peer review only - http://bmjopen.bmj.com/site/about/guidelines.xhtml

For peer review only

For peer review only - http://bmjopen.bmj.com/site/about/guidelines.xhtml

CONSORT 2010 checklist of information to include when reporting a randomised trial*

Section/Topic	Item No	Checklist item	Reported on page No
**Title and abstract**			
	1a	Identification as a randomised trial in the title	Title page
	1b	Structured summary of trial design, methods, results, and conclusions (for specific guidance see CONSORT for abstracts)	Page 1
**Introduction**			
Background and objectives	2a	Scientific background and explanation of rationale	Page 4-5
	2b	Specific objectives or hypotheses	Page 5
**Methods**			
Trial design	3a	Description of trial design (such as parallel, factorial) including allocation ratio	Page 5
	3b	Important changes to methods after trial commencement (such as eligibility criteria), with reasons	Page 8
Participants	4a	Eligibility criteria for participants	Pages 6-7
	4b	Settings and locations where the data were collected	Pages 6-7
Interventions	5	The interventions for each group with sufficient details to allow replication, including how and when they were actually administered	Pages 8-9
Outcomes	6a	Completely defined pre-specified primary and secondary outcome measures, including how and when they were assessed	Pages 12-13
	6b	Any changes to trial outcomes after the trial commenced, with reasons	Page 8
Sample size	7a	How sample size was determined	Page 14
	7b	When applicable, explanation of any interim analyses and stopping guidelines	N/A
Randomisation:			
Sequence generation	8a	Method used to generate the random allocation sequence	Page 14
	8b	Type of randomisation; details of any restriction (such as blocking and block size)	Page 14
Allocation concealment mechanism	9	Mechanism used to implement the random allocation sequence (such as sequentially numbered containers), describing any steps taken to conceal the sequence until interventions were assigned	Page 14
Implementation	10	Who generated the random allocation sequence, who enrolled participants, and who assigned participants to interventions	Page 14
Blinding	11a	If done, who was blinded after assignment to interventions (for example, participants, care providers, those	Page 14

For peer review only

For peer review only - http://bmjopen.bmj.com/site/about/guidelines.xhtml

		assessing outcomes) and how	
	11b	If relevant, description of the similarity of interventions	N/A
Statistical methods	12a	Statistical methods used to compare groups for primary and secondary outcomes	Page 14-15
	12b	Methods for additional analyses, such as subgroup analyses and adjusted analyses	Page 15
**Results**			
Participant flow (a diagram is strongly recommended)	13a	For each group, the numbers of participants who were randomly assigned, received intended treatment, and were analysed for the primary outcome	Figure 1 Appendices 2,3,8
	13b	For each group, losses and exclusions after randomisation, together with reasons	Figures 1 Appendices 2,3,8
Recruitment	14a	Dates defining the periods of recruitment and follow-up	Page 7
	14b	Why the trial ended or was stopped	Page 7
Baseline data	15	A table showing baseline demographic and clinical characteristics for each group	Table 1
Numbers analysed	16	For each group, number of participants (denominator) included in each analysis and whether the analysis was by original assigned groups	Table 1
Outcomes and estimation	17a	For each primary and secondary outcome, results for each group, and the estimated effect size and its precision (such as 95% confidence interval)	Tables 1-4
	17b	For binary outcomes, presentation of both absolute and relative effect sizes is recommended	N/A
Ancillary analyses	18	Results of any other analyses performed, including subgroup analyses and adjusted analyses, distinguishing pre-specified from exploratory	N/A
Harms	19	All important harms or unintended effects in each group (for specific guidance see CONSORT for harms)	Appendix 7
**Discussion**			
Limitations	20	Trial limitations, addressing sources of potential bias, imprecision, and, if relevant, multiplicity of analyses	Page 24
Generalisability	21	Generalisability (external validity, applicability) of the trial findings	Pages 24
Interpretation	22	Interpretation consistent with results, balancing benefits and harms, and considering other relevant evidence	Pages 23-26
**Other information**			
Registration	23	Registration number and name of trial registry	Page 3
Protocol	24	Where the full trial protocol can be accessed, if available	Page 3
Funding	25	Sources of funding and other support (such as supply of drugs), role of funders	Page 26

For peer review only

*We strongly recommend reading this statement in conjunction with the CONSORT 2010 Explanation and Elaboration for important clarifications on all the items. If relevant, we also recommend reading CONSORT extensions for cluster randomised trials, non-inferiority and equivalence trials, non-pharmacological treatments, herbal interventions, and pragmatic trials. Additional extensions are forthcoming: for those and for up to date references relevant to this checklist, see www.consort-statement.org.

For peer review only

For peer review only - http://bmjopen.bmj.com/site/about/guidelines.xhtml

BMJ Open

A pilot cluster randomised trial of an evidence-based intervention to reduce avoidable hospital admissions in nursing home residents (Better Health in Residents of Care Homes with Nursing - BHiRCH-NH study)

Journal:	*BMJ Open*
Manuscript ID	bmjopen-2020-040732.R2
Article Type:	Original research
Date Submitted by the Author:	02-Nov-2020

Complete List of Authors:	Sampson, Elizabeth; UCL, Division of Psychiatry<br>Feast, Alexandra; UCL, Division of Psychiatry<br>Blighe, Alan; University of Bradford Faculty of Health Studies, Ccentre for Applied Dementia Studies<br>Froggatt, Katherine ; Lancaster University Faculty of Health and Medicine, Faculty of Health and Medicine; Lancaster University<br>Hunter, Rachael; UCL, Research Dept of Primary Care and Population Health<br>Marston, Louise; UCL<br>McCormack, Brendan; Queen Margaret University School of Health Sciences, Nursing; Queen Margaret University School of Health Sciences,<br><br>Nurock, Shirley; UCL, Division of Psychiatry<br>Panca, Monica; University College London, Primary Care and Population Health<br>Powell, Catherine; University of Bradford Faculty of Health Studies, Centre for Applied Dementia Studies<br>Rait, Greta; UCL, Primary Care<br>Robinson, Louise; Newcastle University, Institute for Ageing, Institute for Health and Society<br>Woodward-Carlton, Barbara; University of Bradford Faculty of Health Studies, Ccentre for Applied Dementia Studies<br>Young, John; University of Leeds, Academic Unit of Elderly Care and Rehabilitation<br>Downs, Murna; University of Bradford, Bradford Dementia Group

**Primary Subject Heading**:	Geriatric medicine
Secondary Subject Heading:	Health services research, General practice / Family practice, Health economics, Nursing
Keywords:	GERIATRIC MEDICINE, PRIMARY CARE, HEALTH ECONOMICS, Dementia < NEUROLOGY

I, the Submitting Author has the right to grant and does grant on behalf of all authors of the Work (as defined in the below author licence), an exclusive licence and/or a non-exclusive licence for contributions from authors who are: i) UK Crown employees; ii) where BMJ has agreed a CC-BY licence shall apply, and/or iii) in accordance with the terms applicable for US Federal Government officers or employees acting as part of their official duties; on a worldwide, perpetual, irrevocable, royalty-free basis to BMJ Publishing Group Ltd ("BMJ") its licensees and where the relevant Journal is co-owned by BMJ to the co-owners of the Journal, to publish the Work in this journal and any other BMJ products and to exploit all rights, as set out in our licence.

The Submitting Author accepts and understands that any supply made under these terms is made by BMJ to the Submitting Author unless you are acting as an employee on behalf of your employer or a postgraduate student of an affiliated institution which is paying any applicable article publishing charge ("APC") for Open Access articles. Where the Submitting Author wishes to make the Work available on an Open Access basis (and intends to pay the relevant APC), the terms of reuse of such Open Access shall be governed by a Creative Commons licence – details of these licences and which Creative Commons licence will apply to this Work are set out in our licence referred to above.

Other than as permitted in any relevant BMJ Author's Self Archiving Policies, I confirm this Work has not been accepted for publication elsewhere, is not being considered for publication elsewhere and does not duplicate material already published. I confirm all authors consent to publication of this Work and authorise the granting of this licence.

For peer review only

A pilot cluster randomised trial of an evidence-based intervention to reduce avoidable hospital admissions in nursing home residents (Better Health in Residents of Care Homes with Nursing - BHiRCH-NH study)

Elizabeth L Sampson,[1,2] Alexandra Feast,[1] Alan Blighe,[3] Katherine Froggatt,[4] Rachael Hunter,[5] Louise Marston,[5] Brendan McCormack,[6] Shirley Nurock,[1] Monica Panca,[5] Catherine Powell,[7] Greta Rait,[5] Louise Robinson,[8] Barbara Woodward-Carlton,[3] John Young,[9] Murna Downs[3]

Corresponding author
Elizabeth L. Sampson
UCL Division of Psychiatry
6th floor, Wing B, Maple House
149 Tottenham Court Road
London
W1T 7NF
02076799500
e.sampson@ucl.ac.uk

[1] Marie Curie Palliative Care Research Department, Division of Psychiatry, University College London, UK.
[2] Barnet Enfield and Haringey Mental Health Trust Liaison Psychiatry Team, North Middlesex University Hospital, London, UK.
[3] Centre for Applied Dementia Studies, University of Bradford, Bradford, BD7 1DP, UK.
[4] International Observatory on End of Life Care, Lancaster University, Lancaster, UK.
[5] Department of Primary Care and Population Health and Priment Clinical Trials Unit, University College London, London, UK.
[6] Divisions of Nursing, Occupational Therapy & Arts Therapies, School of Health Sciences, Queen Margaret University, Edinburgh, UK.
[7] School of Pharmacy and Medical Sciences, University of Bradford, Bradford, UK
[8] Newcastle University Institute for Ageing and Institute for Health & Society, Newcastle University, Newcastle upon Tyne, UK.
[9] Academic Unit of Elderly Care and Rehabilitation, University of Leeds, Bradford Institute for Health Research, Bradford, UK.

Word count: 5285

For peer review only

ABSTRACT

Objectives: To pilot a complex intervention to support healthcare and improve early detection and treatment for common health conditions experienced by nursing home (NH) residents.

Design: Pilot cluster randomised controlled trial.

Setting: 14 NHs (7 intervention, 7 control) in London and West Yorkshire.

Participants: NH residents, their family carers and staff.

Intervention: Complex intervention to support healthcare and improve early detection and treatment of urinary tract and respiratory infections, chronic heart failure and dehydration, comprising: 1) "stop and watch (S&W)" early warning tool for changes in physical health, 2) condition-specific care pathway and 3) Situation, Background, Assessment and Recommendation tool to enhance communication with primary care. Implementation was supported by Practice Development Champions, a Practice Development Support Group and regular telephone coaching with external facilitators.

Outcome measures: Data on NH (quality ratings, size, ownership), residents, family carers and staff demographics during the month prior to intervention and subsequently, numbers of admissions, accident and emergency visits and unscheduled GP visits monthly for 6 months during intervention. We collected data on how the intervention was used, healthcare resource use and quality of life data for economic evaluation. We assessed recruitment and retention and whether a full trial was warranted.

Results: We recruited 14 NHs, 148 staff, 95 family carers and 245 residents. We retained the majority of participants recruited (95%). 15% of residents had an unplanned hospital admission for one of the four study conditions. We were able to collect sufficient questionnaire data (all over 96% complete). No NH implemented intervention tools as planned. Only 16 S&W forms and 8 Care Pathways were completed. There was no evidence of harm.

Conclusions: Recruitment, retention and data collection processes were effective but the intervention not implemented. A full trial is not warranted.

For peer review only - http://bmjopen.bmj.com/site/about/guidelines.xhtml

Trial registration: ISRCTN74109734 (registered 6/11/2017)

https://doi.org/10.1186/ISRCTN74109734

Original protocol: *BMJ Open*. 2019;9(5):e026510. doi:10.1136/bmjopen-2018-026510

Keywords: cost analysis; dehydration; dementia; geriatric medicine; heart failure; nursing homes; primary care; respiratory infections; urinary tract infections

ARTICLE SUMMARY

Strengths and limitations of this study

- The intervention was adapted from a successful USA programme, co-designed with residents, family carers and staff to fit with the UK nursing home context
- Successful recruitment and retention of nursing homes, their staff, residents and family carers demonstrated the feasibility of our study methods
- It was challenging to collect reliable data on hospital admissions, ambulance and general practitioner visits
- The nature of the intervention in this cluster randomised trial meant that outcome data were not collected blind to allocation for intervention or control group.

INTRODUCTION

Currently in the United Kingdom (UK) more than 420,000 people aged over 65 years live in residential care, of which approximately 220,000 reside in care homes with nursing (referred to in this paper as “nursing homes” (NHs)).[1] Most UK NHs are owned by private companies and residents pay on a means-tested basis. Unlike some European NHs, for example in the Netherlands, there is no on-site provision of medical care. NH residents are served by a general practitioner and other visiting staff such as specialist nurses. NH residents have complex healthcare needs with high levels of multi-morbidity, frailty and dementia. The King’s Fund [2] and British Geriatrics Society [3] have raised concerns about the inconsistency and quality of healthcare provision to NHs.

There has been a 63% increase in all-cause hospital admissions from NHs between 2011-2015.[4] As well as causing distress to residents, their families and staff, hospitalisation is expensive for health and social care systems, costing an estimated £1.2 billion per-annum in the UK.[5] Hospital admission increases risk of decline in functional ability, delirium, adverse events and prolonged stays.[6,7]

Ambulatory Care Sensitive Conditions (ACSCs) are “conditions that can lead to unplanned hospital admissions that may have been avoidable or manageable by timely access to medical care in the community”.[8,9] Four ACSCs contribute to a 30% of hospitalisations from NHs:[5] respiratory infections;[10,11-13] acute exacerbation of chronic heart failure;[14,15] urinary tract infections;[12,16] and dehydration.[12]

A number of interventions have been developed to enhance healthcare in NHs, falling broadly into two categories; single component interventions (predominantly advance care planning or single disease care pathways, e.g. for pneumonia) and multicomponent interventions which

For peer review only - http://bmjopen.bmj.com/site/about/guidelines.xhtml

include enhancing staff knowledge and skills,[17] clinical guidance and decision-support tools (care pathways), engaging with families,[18] and input from geriatricians or nurse practitioners.[19] Research highlights the importance of collaborative intervention development with staff,[20] residents and families,[18] and using local champions to support implementation.[15] "INTERACT" (Interventions to Reduce Acute Care Transfers), developed in the USA, focusses on managing acute changes in residents' condition and reduces transfers to acute hospitals.[15] We worked with stakeholders and our family Carer Reference Panel (CRP), to develop and adapt INTERACT for use in the UK.[21] This included 18 semi-structured interviews and three co-design workshops over five months, with amendments made to the intervention after each workshop. Participants comprised 22 diverse stakeholders (two NH managers, three care assistants, eight nurses, four general practitioners, three family carers, geriatrician and a quality improvement manager) (paper in preparation). The Promoting Action in Health Services (PARiHS) framework underpinned co-design of implementation support and guidance.[22]

Aim and objectives

Our aim was to indicate whether a definitive study of the BHiRCH-NH intervention is warranted.

Primary objective

To indicate whether the intervention was acceptable and feasible.

Secondary objectives

1. Establish whether consent procedures facilitate collection of sufficient individual-level data
2. Assess intervention fidelity

For peer review only - http://bmjopen.bmj.com/site/about/guidelines.xhtml

3. Assess the effectiveness of the implementation strategy and level of staff engagement with the intervention
4. Indicate whether the intervention would be sustainable outside the trial context
5. Assess potential primary and secondary outcome measures for a definitive trial
6. Measure completeness of data collection, documentation, return rate of questionnaires and assess potential primary and secondary outcomes for a definitive trial
7. Assess feasibility of collecting data for economic evaluation.

METHODS

Trial design

A pilot cluster randomised trial in NHs, conducted and reported as per the CONSORT guidance.[23] Detailed methods are described in the trial protocol paper.[21] The NH was the unit of allocation (seven intervention and seven control sites).

Patient and public involvement in research

The project was developed in collaboration with UK Dementia and Neurodegenerative Diseases Network (DeNDRoN). Patient and Public Involvement representatives (SN and BW-C) were grant co-applicants. Two Carer reference Panels (CRPs), ensured public involvement at all stages. Each comprised eight family carers of people with dementia and a person living with dementia, supported by Alzheimer's Society research volunteer network. They collaborated on intervention design, recruitment and consent processes, accessibility of information leaflets, data collection, interpretation and dissemination.

For peer review only - http://bmjopen.bmj.com/site/about/guidelines.xhtml

Study population and eligibility criteria

Nursing homes

We recruited 14 NHs (eight in West Yorkshire and six in London) with adequate staffing to implement the intervention and support research. These were identified via local Clinical Research Networks, and the ENRICH (Enabling Research in Care Homes) Network, purposively selected to include a range of providers (large and small chains, independent providers), urban, suburban and rural. Managers, regional managers, or owners gave written permission. The intervention was an enhanced version of “usual care” implemented at NH level. Therefore, individual consent was not required and all staff were involved in delivering the intervention to all NH residents.

Individual participants

We invited all English-speaking staff and residents over 65 years and their carers (family members or friends) to participate in individual-level data collection until we recruited approximately 20 residents and ten staff from each NH. We excluded residents receiving end-of-life care or those who did not wish to be involved in research.

Consent procedures

Ethical approval was given by the Queen Square London Research Ethics Committee (reference 17/LO/1542).

Residents

The NH manager or deputy manager identified all potentially eligible residents. If necessary, we conducted a capacity assessment regarding trial participation, adhering to the UK Mental Capacity Act (2005). If the resident lacked capacity, we used a personal consultee (friend or family), or if not available, a professional consultee (health or social care staff with a

For peer review only - http://bmjopen.bmj.com/site/about/guidelines.xhtml

professional relationship to the resident but no connection with the project). If a resident lost capacity during the study a consultee was found.

Family carers and staff

We invited a family carer and NH staff associated with residents recruited to the study to answer questionnaires and they gave informed consent for this.

INTERVENTION

The pilot trial ran for ten months (November 2017-August 2018). Sites were set up in months one and two, in month three we collected pre-intervention (baseline) data over four weeks before the intervention was implemented. The intervention ran for six months with final data collection and site closure in month ten. We planned for the trial to run for 16 months from November 2017 but had to reduce follow-up due to delays in obtaining ethical approvals. Thus, timing of data collection differs from our protocol.[21]

Implementation support

This was developed consistent with the PARIHS Framework to ensure implementation, matched individual contexts. It was important for Practice development Champions (PDCs) to decide on how they approached this, given the philosophy of quality collaboratives and 'champions' in place. This enhanced the role of PDCs in developing their own approach.

Workshop

The research team delivered a one-day workshop to two PDCs from each NH comprising an introduction to four key ACSCs (respiratory and urinary tract infections, dehydration, acute exacerbation of chronic heart failure) and elements of how to bring about organizational change. We gave an overview of intervention materials.

For peer review only - http://bmjopen.bmj.com/site/about/guidelines.xhtml

Introductory meeting

Researchers held a project initiation meeting with each NH manager and available staff to highlight key intervention components, how staff should deliver the intervention and how this may be integrated into existing local systems. The PARIHS framework supported PDCs and their colleagues to disseminate this information to the wider NH staff.

Ongoing implementation support

1. We supported PDCs with a project handbook including information on approaches to change used in this project; intervention implementation within differing contexts; tips to help teams learn and act alongside the people for whom they care; and information on enhanced leadership capabilities. PDCs were also guided through the *Practice Development Workbook for Nursing, Health and Social Care Teams: Resources for Health and Social Care Teams*.[24]
2. We expected PDCs to establish a Practice Development Support Group (PDSG) to support their work in the NH. This "quality collaborative" approach involves diverse stakeholders working together to close the gap between actual and potential practice.[25]
3. The programme manager made weekly contact with the PDCs to collect information on how the intervention was working and was available for advice "as required". PDCs were offered monthly telephone coaching by senior nurse researchers on our team (BMcC and KF).

The intervention (BHiRCH-NH)

This consisted of three key components, adapted from the INTERACT programme [26] and paper-based as UK NHs have variable use of electronic records:

1. *Stop and Watch Early Warning Tool*: Care assistants or nurses used this when they noted a change in a resident's condition. They circled observed changes, notified the nurse and

For peer review only - http://bmjopen.bmj.com/site/about/guidelines.xhtml

placed the tool in the resident's NH records.

2. *Care Pathway*: This was a two-step clinical guidance and decision support system, focussing on symptoms and signs of four key ACSC conditions (acute exacerbation of chronic heart failure, respiratory and urinary tract infections, dehydration). The initial "Primary" assessment comprised screening questions with the potential to trigger a more detailed "Secondary" assessment. If the Primary or Secondary Assessment result was ambiguous, the *Care Pathway* was administered at 6-hour intervals, until concerns had resolved and/or appropriate intervention was instigated. The nurse recorded the outcome of the Primary and Secondary Assessment and their care plan in the resident's records and decided on the next course of action. This may have included further monitoring using the *Stop and Watch Early Warning Tool*, treatment initiated in the NH, or communication with primary care using the *SBAR*. Copies of the completed *Care Pathway* were kept with the resident's record.
3. *The SBAR method:* A structured method for communicating critical information to primary care used by nurses to seek primary care intervention for the resident after the *Care Pathway* indicated a risk of decline.

Treatment as usual (TAU) nursing homes

Residents received usual care according to existing local policy and practice. We permitted all medications and treatments.

Data collection

Nursing homes

A Research Facilitator, employed by the NH provided pseudo-anonymised data at the NH level. We collected data for four weeks pre-intervention (baseline) including staff turnover and the number of beds available to new residents. For four weeks pre-intervention and then monthly for

6 months we documented the total number of contacts with GPs, ambulances, A&E attendances and hospital admissions.

Residents

We collected data pre-intervention on age, gender, ethnicity, marital status and highest level of education. Functional status was measured using the Barthel Index [27] pre-intervention, and at 6 months. We collected data for the month pre-intervention and then for the following 6 months on healthcare use and QoL data for health economic analysis, hospital admission overall and admissions for the four ACSCs of interest, ambulances called, out-of-hours GP visits or telephone contacts, A&E attendances and deaths.

Family carers

Pre-intervention we collected data on socio-demographics including age, gender, ethnicity and marital status and preferred role, i.e. how involved they would like to be with the resident's medical care.

Staff

For contextual understanding we documented pre-intervention; staff age, gender, and education level and characteristics of their work (qualifications, role, length of employment, shift pattern and first language). Pre-intervention and at six months we measured the extent to which they perceived the organisation supported person centred care (Organisational Support for Person-centred Care (P-CAT))[28] and the Nurse Ratings of Communication with Primary Care Questionnaire.[29]

For peer review only - http://bmjopen.bmj.com/site/about/guidelines.xhtml

Outcomes

We collected data in three domains: 1) individual-level data on NH residents, their carers and staff where consent had been obtained, 2) system-level data collected by a Research Facilitator to provide pseudo-anonymised data at the NH level, and 3) process data collected by the study team (table 1).

For peer review only

Table 1: Summary of data collected, outcome measures and time schedule

Data collected and tool used		Pre-intervention	Monthly	6 months
Resident				
Socio-demographics	Age, gender, ethnicity, marital status, highest level of education	S	-	-
Service use in the prior month	Client Service Receipt Inventory (CSRI). Calculates service and total care costs	S	S	-
Functional status	The Barthel Index	S	-	S
Resident quality of life-self rated	EQ-5D-5L self-rated health index and visual analogue scale of current health state	P	-	P
Resident quality of life-proxy rated	EQ-5d-Proxy family carer or staff member view of the resident's QoL	FC/S	-	FC/S
Family carer				
Socio-demographics	Age, gender, ethnicity, marital status, years of schooling, highest level of education	FC	-	-
Quality of life	EQ-5D-5L	FC	-	FC
Preferred role	How much and how they like to be involved in the residents' care	FC	-	-
Staff				
Staff socio-demographics	Age, gender, ethnicity, number of years of education,	R	-	-
Staff work characteristics	Highest qualification, role in nursing home, length of service, shift pattern, first language	R	-	-
Organisational support for person-centered care	The Person-Centred Care Assessment Tool (P-CAT)	S	-	S
Communication with primary care	Nurse-GP Communication Needs Assessment Questionnaire	S	-	S
Perceived knowledge and skills for early detection in changes in health	Developed from feasibility study. Assesses key knowledge and skills needed to implement the intervention. Rated on 5-point Likert scale.	S	-	S
**System-level data**				
Number of hospital admissions	Respiratory infection, exacerbation of CHF, UTI and dehydration	S	S	-
"Avoidability" of admissions	Structured Implicit Record Review (SIRR; Saliba et al., 2000)	S	S	-
Use of Primary assessment tool	Respiratory infection, exacerbation of CHF, UTI and dehydration?	S	S	-
Use of Secondary assessment	Respiratory infection, exacerbation of CHF, UTI and dehydration	S	S	-
Out of hours GP contacts	GP visits or telephone contact	S	S	-
Ambulances and hospital use	Number of hospital admissions, A&E attendances and readmissions	S	S	-
Deaths in the last calendar month		S	S	-
Staff turnover		S	-	-
Nursing home occupancy level	Number of available beds to new residents	S	-	-

Measure assessed by: P, participant; FC family carer; R researcher; S, nursing home staff

For peer review only - http://bmjopen.bmj.com/site/about/guidelines.xhtml

Primary objective

To ascertain whether the intervention was acceptable and feasible we collected data monthly from the NH on intervention use in practice; number of “Stop and Watch” Early Warning Tools and Primary (initial screening) and Secondary (more detailed) assessment tools completed. Where we had permission to collect individual-level resident data, we monitored participants monthly for SAEs defined as “any untoward occurrence that resulted in death, was considered life-threatening at the time of the event, required hospitalisation or prolongation of existing hospitalisation, resulted in persistent or significant disability or incapacity or was any other important medical condition”.

Secondary objectives

1. *Establish whether consent procedures facilitate collection of sufficient individual-level data*

We collected data on consent and recruitment rates of residents, carers and staff.

2. *Assess intervention fidelity*

To explore intervention fidelity two nurse researchers aimed to review a convenience sample of five records for residents admitted to hospital, or received treatment in the NH, for ACSCs. They used a free-text review sheet to record references to trial intervention tools (Stop & Watch, the Care Pathway and SBAR) and assessed compliance with the care pathways. We noted where NHs made amendments to the structure or content of the care pathway.

3. *Assess the effectiveness of the implementation strategy and level of staff engagement with the intervention*

PDCs were offered monthly telephone coaching for advice and to reflect on activities and achievements. They kept an activity log of work with Practice Development Support Groups to document the level of facilitation required to support implementation. Qualitative interviews were conducted to better understand the barriers and facilitators to implementation. Learning from the implementation strategy will be presented in a separate paper.

For peer review only - http://bmjopen.bmj.com/site/about/guidelines.xhtml

4. Indicate whether the intervention would be sustainable outside the trial context

Data from objectives 2-3 were considered by the independent project steering group at the end of the study.

5. Assess potential primary and secondary outcomes for a definitive trial

To explore the impact of enhanced healthcare in the NH we documented hospital admissions for the four key ACSCs (acute exacerbation of chronic heart failure, respiratory and urinary tract infections, dehydration). Number (%) of residents requiring one or more ambulance calls, one or more unscheduled out of hours GP visits or phone calls and having one or more accident and emergency department visits were documented as potential secondary outcomes.

We tested the assumption that a hospitalisation for an ACSC was a proxy for an avoidable hospital admission using the Structured Implicit Record Review (SIRR) tool.[30] Two independent experts (geriatrician and community nurse) used the SIRR, which takes account of the resident's pre-existing health, any advance directives and the care options available at the time to assess the "avoidability" of the admission.

6. *Measure completeness of data collection and documentation, return rate of questionnaires*

We assessed completeness of outcome measures, data collection and return rate of questionnaires.

7. Assess feasibility of collecting data for economic evaluation

We collected data on resident service use with the Client Service Receipt Inventory (CSRI),[31] completed pre-intervention and then monthly for 6 months. Data on quality of life (QoL) was collected using the self-completed EQ-5D-5L [32] questionnaire where participants had the capacity to do this. Where a resident could not complete the EQ-5D-5L questionnaire, the proxy version was completed by the carer or a member of staff. We also collected data on carer QoL using the EQ-5D-5L questionnaire. All QoL data were collected pre-intervention and at 6 months.

For peer review only - http://bmjopen.bmj.com/site/about/guidelines.xhtml

Sample size

This was a pilot study so no sample size calculation was conducted. The number of NHs was chosen on pragmatic grounds to allow testing of study procedures and variability in settings.

Randomisation

NHs were randomised prior to intervention; four in West Yorkshire and three in Greater London (seven total) to the intervention and four in Yorkshire and three in Greater London (seven total) to "usual care", stratified by location. We used the SAS statistical programme to generate a randomisation list drawn-up by a clinical trials unit statistician not involved in the study.

Blinding

This was not feasible for staff collecting data. Statisticians and health economists were blinded to allocation. The randomisation variable was supplied to them unlabelled, and the main analysis was completed using this.

Data management

Data were entered onto paper case report forms (CRFs) and then into an encrypted password protected database in accordance with the UK Data Protection Act and General Data Protection Regulation (GDPR). We followed a standardised process for database lock.

Statistical methods

As a pilot study, analyses are mainly descriptive (counts, means, standard deviation (SD), medians with interquartile range (IQR)) focussing on recruitment, participant characteristics, other baseline and outcome variables, loss to follow-up and tabulation of SAEs. We summarised completeness of data on outcome measures and questionnaire response rates.

Economic evaluation

For peer review only - http://bmjopen.bmj.com/site/about/guidelines.xhtml

We calculated costs associated with the intervention, including costs of staff training and implementation. Resource use associated with hospital admissions, primary care and NHS and social care services were collected using the CSRI and costed from the NHS/ personal social services (PSS) perspective.[33-35] All costs are reported in 2016/2017 British Pounds (£). Quality-adjusted life years (QALYs) for residents were calculated using the EQ-5D-5L questionnaire and associated algorithms mapping the 5L descriptive system data onto the 3L valuation set as recommended by the National Institute for Health and Care Excellence (NICE).[36-39] We report only results based on QALYs calculated using the resident self-completed EQ-5D-5L questionnaire as these had higher return. There was no discounting of costs or QALYs given they were reported over 6 months only. For missing data we used multivariate imputation by chained equation, generating 20 imputed data sets. For each of these, we ran 1,000 bootstrap replications using non-parametric bootstrapping. Bootstrap results were combined to calculate the mean values for costs and utilities and the standard errors around the imputed values, used to calculate 95% CI around point estimates. To report the probability that the intervention is cost-effective compared to TAU for a range of values of willingness-to-pay (WTP) for a QALY gained, the bootstrap results have been used to generate a cost-effectiveness acceptability curve (CEAC) [40] and the probability that the intervention is cost-effective compared to TAU at a £20,000 for a QALY gained reported.

RESULTS

Here, we give trial results with full data given in the supplementary file appendices to ensure transparency of reporting. In depth evaluation of implementation will be published separately.

For peer review only - http://bmjopen.bmj.com/site/about/guidelines.xhtml

Recruitment

Nursing homes

We recruited the target number of 14 NHs and randomised as planned; seven intervention (three London, four Yorkshire) and seven control (three London and four Yorkshire). One Yorkshire intervention NH was closed by its owners and dropped out pre-intervention. A further intervention NH in London dropped out following PDC training as they were unable to implement the intervention. Most NHs were privately managed with a median 50 residents (IQR 34, 68). The majority (73%) were “dementia registered”. In terms of CQC ratings, one home (7%) was “outstanding”, eleven (79%) “good” and two (14%) “required improvement” (supplementary file, appendix 1).

Residents

We recruited 237 residents (figure 1), two thirds were female, predominantly white (90%) with a median age of 86 years (IQR 80, 91).

Figure 1 here

The median Barthel Index score was low at 27 (IQR 9, 64) indicating a high level of dependency in activities of daily living. Only six residents (3%) had an admission for an ACSC in the pre-intervention period, 11 (5%) residents had at least one ambulance called and 12 (5%) had an unscheduled GP visit or telephone contact (table 2).

Family carers

We recruited 91 family carers, two thirds were female (table 2, supplementary file, appendix 2). Median age was 63 years (IQR 57, 71). Most (91%) wished to be involved in noticing early changes in the resident’s health).

Table 2: Characteristics of residents and family carers

Characteristic	Cohort	TAU	BHiRCH-NH
	n or median (% or IQR)		
**RESIDENTS**			
**Demographics**	N=234	N=137	N=97
Male	73 (31)	46 (34)	27 (28)
Age	86 (80,91)	86 (80, 91)	84(78, 91)
**Ethnicity**	N=225	N=131	N=94
White	203 (90)	117 (89)	86 (91)
Black	14 (6)	9 (7)	5 (5)
Asian	5 (2)	4 (3)	1/(1)
Other	3 (1)	1 (1)	2(2)
**Marital status**	N=223	N=127	N=96
Married or cohabiting	49 (22)	28 (22)	21 (22)
Single	59 (26)	40 (32)	19 (20)
Divorced or widowed	115 (52)	59 (46)	56 (58)
**Education**	N=184	N=133	N=71
Completed years of education	11 (9,12)	11(10, 12)	11(9, 11)
No qualifications or GCSE or equivalent	107 (58)	63 (56)	44 (62)
A Level/NVQ/ HNC/ HND or equivalent	18 (10)	11 (10)	7 (10)
Degree or higher degree	23 (13)	14 (12)	9 (13)
Other qualification	36 (20)	25 (22)	11 (15)
**Function**			
Barthel Index score	27 (9, 64)	27(9, 66)	30(8, 63)
**CARERS**			
**Demographics**	N=91	N=56	N=35
Male	31 (34)	17 (30)	14 (40)
Age	63 (57, 71)	62(57, 71)	64(58, 74)
**Ethnicity**	N=87	N=52	N=35
White	72 (83)	43 (83)	29 (83)
Black	12 (14)	7 (13)	5 (14)
Asian	3 (3)	2 (4)	1(3)
**Marital status**	N=87	N=53	N=34
Married or cohabiting	65 (75)	36 (68)	29 (85)
Single	10 (11)	8 (15)	2 (6)
Divorced or widowed	12 (14)	9 (17)	3 (9)
**Education**	N=86	N=53	N=33
Completed years of education	11 (11,12)	12(11, 13)	11(11, 12)
No qualifications or GCSE or equivalent	35 (41)	21 (40)	14 (42)
A Level/NVQ/ HNC/ HND or equivalent	13 (15)	9 (17)	4 (12)
Degree or higher degree	26 (30)	14 (26)	12 (36)
Other qualification	12 (14)	9 (17)	3 (9)
**Preferred role**	N=87	N=52	N=35
Noticing early signs of changes in health	79 (91)	49 (94)	30 (86)
Informing staff about early signs of changes in health	77 (89)	48 (92)	29 (83)
Educating staff about how early signs of changes in health present	51 (59)	28 (54)	23 (66)
Educating care staff about health history of their family member	57 (66)	33 (63)	24 (69)
Prefer not to be involved	5 (6)	2 (4)	3 (9)
Other	18 (21)	9 (17)	9 (26)

For peer review only - http://bmjopen.bmj.com/site/about/guidelines.xhtml

Staff

We recruited 132 staff (supplementary file, appendix 3), with a median age of 42 years (IQR 30, 53), 12% were male, 50% of nurses spoke English as a first language and 59% of staff described themselves as white. Most staff (71%) had worked at the NH for a year or more and 30% were qualified nurses. Scores on the P-CAT scale (possible range 13-65) were generally positive with a median score of 49 (IQR 46, 53) (supplementary file, appendix 4). Most nurses were positive about the quality of communications they had with GPs (supplementary file, appendix 5) and their self-rated knowledge and skills (supplementary file, appendix 6).

Primary objective-feasibility and acceptability

Use of the intervention

Across the five intervention NHs, only sixteen Stop and Watch forms were completed of which eleven came from a single NH. Eight Care Pathways were reported as completed but only three were located by the study team. There was a median of one Stop and Watch form (IQR 0,3) and a median of zero Care Pathways (IQR 0,2) completed per month. In a few cases, routine clinical observations (e.g. temperature, blood pressure etc.) were carried out, but not reported systematically or presented as part of a coherent assessment plan. One home had a policy of recording routine observations once per month.

Serious adverse events

There were no differences in SAEs between TAU and intervention groups. There were 104 SAEs in 74 residents during the study and 33 residents died (19 TAU and 14 intervention). Of the 104 SAEs, hospitalisation (any cause) was the most common (N= 50) (supplementary file, appendix 7).

For peer review only - http://bmjopen.bmj.com/site/about/guidelines.xhtml

Secondary objectives

1. Resident consent procedures and collection of sufficient individual level data

We screened 680 residents, 557 met eligibility criteria and 245 were recruited (35% recruitment). Taking into account that two NHs dropped out, leaving 12 in the study, we reached our target of 240 residents (20 residents per NH). Most eligible residents (364, 65%) did not have capacity to consent to participate in the study. Of recruited residents, 73% completed the study (supplementary file, appendix 8).

2. Assess intervention fidelity

It was not possible to assess fidelity to the intervention as the intervention was not implemented as intended and the documentation required to assess fidelity was not available.

3. Assess the effectiveness of the implementation strategy and level of NH staff engagement with the intervention

The implementation strategy was not effective and NH staff did not engage with or use the intervention tools. Data and learning from implementation will be presented in a separate paper.

4. Investigate whether the intervention would be sustainable outside the context of a trial

The intervention was not widely used; 16 Stop and Watch forms were completed and we found eight completed Care Pathway documents. We concluded the intervention in its current form was not sustainable.

5. Assess potential primary and secondary outcome measures for a definitive trial

Rates of hospitalisation for ACSCs

At baseline the rates of hospitalisation for ACSCs (respiratory infection, exacerbation of CHF, UTI and dehydration), our potential primary outcome, were low and 0.4% of the cohort had an

For peer review only - http://bmjopen.bmj.com/site/about/guidelines.xhtml

admission for respiratory infections, 1% for UTI, none for dehydration and 0.4% had an admission for chronic heart failure in the month before the trial started. There were six admissions in total from the 235 residents in the study. Considering the whole six-month study period, 25 study participants (15%) had an unplanned hospital admission for one of the ACSCs. The low rates of unplanned hospital admission (per 100 person months) for these ACSCs suggest this is not an optimal primary outcome measure for future studies (table 3). Considering secondary outcome measures for a definitive trial, we found a slightly higher proportion of participants (n=38, 16%) had an accident or emergency attendance during the follow up period, 42 (18%) had at least one ambulance called, 29 (12%) had an unscheduled (out-of-hours) GP visit and 21 (11%) died. The incidence of these events was still relatively low and not sufficiently frequent as definitive study outcomes.

Hospitalisation for an ACSC as a proxy for avoidable hospital admission

We intended to use the Structured Implicit Record Review (SIRR) tool to assess the appropriateness of 30 resident admissions for one of the four ACSCs, but it was not always possible to identify the reasons for admission and these were low overall. We therefore expanded our sample to include hospital admissions for any cause. One NH had no eligible residents, because those admitted to hospital had died and/or their care records were no longer available. We therefore also included residents who died in hospital, as long as their records were still available. We were able to assess 10 admissions from a total of 24, which occurred during the study period. Using the SIRR tool, we deemed three of the ten admissions assessed potentially avoidable. None of the NH care records provided a complete picture of residents' health in the period leading up to admission. This suggested an ACSC admission was not a reliable proxy measure for an "avoidable admission".

For peer review only - http://bmjopen.bmj.com/site/about/guidelines.xhtml

Table 3. System-level outcome data

	Pre-intervention			Over 6 month follow up period	
	Whole cohort	TAU	BHiRCH-NH	TAU	BHiRCH-NH
	n or median (% or (IQR)				
**Study cohort**	N=235	N=139	N=96	N=139	N=96
At least one admission in the last month	6 (3)	2 (1)	4 (4)	19 (14)	16/96
Respiratory infection admission	1 (0.4)	0	1 (1)	8 (6)	5/96
Urinary tract infections admission	2 (1)	1 (1)	1 (1)	5 (4)	2/96
Dehydration admission	0	0	0	0	1/96
Congestive heart failure admission	1 (0.4)	0	1 (1)	0	1/96
At least one ambulance called	11(5)	4 (3)	7 (7)	20 (14)	22/96
At least one out of hours GP visit or telephone contact	12 (5)	11 (8)	1 (1)	14 (10)	15/96
At least one Accident and Emergency attendance	10 (4)	6 (4)	4 (4)	17 (12)	21/96
Died	1 (0.4)	1 (1)	0		
**Nursing home data**					
Number hospital admissions	3 (2, 5)	4 (2, 7)	3 (2, 4)	12 (12, 16)	12 (7, 18)
Number of ambulances called	3 (2,6)	4 (2,9)	3 (2, 6)	12 (11,17)	19 (7, 22)
Unscheduled (out of hours) GP visits or telephone contacts	1 (1,3)	2 (1,3)	1 (1, 3)	8 (7,13)	9 (4, 25)
Accident and Emergency attendances	3 (2,5)	3 (1,4)	3 (2, 6)	12 (11,13)	8 (7, 13)
Rate of hospital admissions per 100 person months		-	-	5.3 (2.3, 8.3)	5.9 (1.7, 7.1)
Rate of ambulances called per 100 person months		-	-	5.7 (2.3, 8.0)	6.0 (2.0, 9.3)
Rate of unscheduled (out of hours) GP visit or contacts per 100 person months		-	-	2.5 (1.8, 4.0)	5.1 (1.1, 6.0)
Rate of Accident and Emergency attendances per 100 person months		-	-	4.3 (2.5, 8.3)	3.9 (2.0, 5.6)

6. Completeness of data collection and documentation, return rate of questionnaires

Care staff-related data

We collected data on most recruited care staff at baseline (N=132). For example, 129 care staff gave demographic details (98%) and 127 (96%) completed the P-CAT scale. Attrition in response to these scales was secondary to NHs dropping out of the study, rather than staff being unwilling or unable to complete them.

Resident-related data

Demographic and functional ability data were available on most residents at baseline (N=235)

For peer review only - http://bmjopen.bmj.com/site/about/guidelines.xhtml

including gender (99%), ethnicity (95%), marital status (98%) and Barthel Index (98%). We cannot be sure that no admissions or visits to acute hospital were missed as NHs did not have centralised systems for collecting these data.

7. Cost and outcome data for use in an economic evaluation, key cost components and probability of cost effectiveness

One NH withdrew after randomisation and therefore, we considered the cost of intervention to be £0 (as "per randomised" approach). Assuming the intervention was offered to all 89 residents randomised to the intervention group, the mean cost per resident would be £74 (95%CI £64 to £84). During follow-up, there were no significant differences in the majority of the components of healthcare resource use between the intervention and TAU groups (supplementary file, appendices 9, 10). Differences in mean utility values and QALYs were not statistically significant when carers assessed their QoL and therefore, no further analysis was performed. The mean total cost of healthcare resource use/resident over 6 months was £1,458 (95%CI £1,351 to £1,566) in the intervention group and £1,233 (95%CI £1,171 to £1,295) in the TAU group (supplementary file, appendix 11). Non-parametric bootstrapping after multiple imputation produced a mean total cost per resident in the intervention group of £1,479 (95%CI £757 to £2,200), compared with £1,271 (95%CI £975 to £1,566) in the TAU group. The mean difference in cost between the BHiRCH-NH and TAU group was £208 (95%CI -£561 to £977). Non-parametric bootstrapping after multiple imputation produced 0.315 (95%CI 0.304 to 0.326) QALYs in the intervention group and 0.298 (95%CI 0.290 to 0.307) QALYs in the TAU group, generating a was statistically significant mean difference in QALYs of 0.016 (95%CI 0.003 to 0.300) (table 4).

For peer review only - http://bmjopen.bmj.com/site/about/guidelines.xhtml

Table 4. Cost-effectiveness of BHiRCH-NH intervention versus TAU: complete case and imputed data analyses

	Incremental cost		QALY gained	
	**Mean**	**(95% CI)**	**Mean**	**(95% CI)**
Base case*	£208	-£561 to £977	0.016	0.003 to 0.300
Complete case^	£352	-£745 to £1,448	0.018	-0.012 to 0.048

*Data include values imputed using multiple imputation with standard errors corrected to account for uncertainty in the imputed values. QALYs gained are adjusted for baseline utility values and nursing home clustering. The incremental costs are adjusted for costs in the 1 month period prior to baseline and nursing home clustering. ^As for the base case analysis except there is no multiple imputation for missing data

The incremental cost per QALY gained of BHiRCH-NH versus TAU was £12,633. Residents receiving the intervention accrued a non-significantly higher cost, and a very small increase in QALYs; the intervention has a 65% probability of being cost-effective at a WTP of £20,000 (supplementary file, appendix 12).

DISCUSSION

Our cluster randomised pilot trial of the BHiRCH-NH intervention in 12 NHs found study processes were effective. We successfully recruited, retained and obtained individual-level data from residents, staff and family carers. Adverse event data did not suggest the intervention caused harm. Our Carer Reference Panel worked with us throughout, advising on study set-up, engaging with homes and potential participants, data collection and contributing to data analysis and interpretation. However, despite excellent recruitment and retention, there was limited engagement with the intervention tools and the support offered for their implementation. The lack of use of the intervention coupled with the economic analysis means that we would not recommend a definitive randomised controlled trial of the BHiRCH-NH intervention. We focussed on four key ACSCs (dehydration, respiratory and urinary tract infections and exacerbation of chronic heart failure) because they are common causes of potentially avoidable

hospital admissions and a significant area of health policy focus. There is lack of national comparative data, but we found lower hospitalisation rates for these conditions than we expected. Categorising admissions to hospital is complex and people may present with broader symptoms i.e. delirium or falls. ACSCs may be more of an administrative label to be used in large data analyses rather than a sensitive tool for assessing the “avoidability” or otherwise of acute hospital admissions.

Implementing practice change in NHs can be challenging. Despite offering monthly telephone support calls during the study at times agreed with PDCs, they often couldn’t speak due to changes in duty rosters, last-minute leave or being too busy. Whilst the INTERACT programme, from which our BHiRCH-NH intervention was adapted, demonstrated reductions in all-cause hospitalisations among actively participating NHs, a subsequent larger randomised controlled implementation trial in 85 USA NHs had no effect on emergency department visits or hospital admissions.[15] Resources, competing demands and instability of NH leadership were all barriers to successful to INTERACT implementation [41] and it is likely that these factors were also present in our study.

Our study has limitations. We recruited NHs, residents, staff and family carers to target, although two NHs dropped-out from the intervention arm. Our NHs may be atypical as 79% were rated as “good” by the CQC, compared to the UK national average of 73%. Residents in our sample were broadly representative of the UK NH population in terms of age and gender.[42] Monthly visits from research fieldworkers and the appointment of Research Facilitators who were existing NH staff fostered positive relationships between NHs and the research team and facilitated access to NH records. We cannot verify that data on hospital attendances, staffing and support from external health care services is complete, as most NHs did not routinely record hospital admissions or ambulance and GP call-outs. Staff self-rated their

knowledge and skills regarding health conditions and their communication with primary care and research assistants collecting outcome data were not blind to NH allocation. We assumed we could use admission for ACSCs as a proxy for avoidable admissions, but even using the SIRR tool [43] it was difficult to identify whether an admission was for an ACSC or not. Future studies should include consent to access hospital records so this can be more thoroughly assessed.

Current UK policy focus on reducing hospitalisations and enhanced health care in NHs has led to significant levels of activity in local health and social care services. Research in such a fast-moving landscape is challenging. NHS England's demonstration projects "Vanguards" and increasing numbers of local quality improvement initiatives means "usual care" will be improving, making trials challenging to conduct. Although our intervention was co-designed with staff, this involved an extra burden of observation and documentation, which was difficult for staff who were already pressed for time. Recent studies conducted in care homes suggest that a higher level of support and facilitation may be required for effective implementation; meetings involving all NH staff may be more successful.[44] The Palliative Care for Older People Steps to Success Programme (PACES), conducted in 78 NHs across seven countries, which involved a complex intervention to improve end of life care, did not improve resident comfort or staff knowledge despite using a train-the-trainer approach.[45] However, the Well-Being and Health for People with Dementia (WHELD) programme was successfully implemented in 69 UK care homes and showed a significant effect on reducing agitation possibly through a much higher intensity of external facilitation that may not be sustainable or cost effective.[46] Since we designed our intervention and conducted our pilot trial, knowledge on improving healthcare for NH residents has increased. Key components for resident health care, identified in the 2017 "Optimal" study were GP involvement supported by integrated external services.[47] These findings are reflected by emerging evidence that change to the wider health and social care system may be more effective in reducing the number of NH residents admitted to hospital.[48]

For peer review only - http://bmjopen.bmj.com/site/about/guidelines.xhtml

CONCLUSIONS

Our co-designed complex intervention for early detection, monitoring and communication of change in residents' health does not warrant a future definitive trial because it was not implemented in practice. It was important that this pilot trial was conducted as this has avoided the risk of embarking on a full trial and subsequent waste of resources and time.[49] Our study contributes to learning in the under-researched but vital field of NH research.

DECLARATIONS

Acknowledgements: The authors would like to thank the members of our carer reference panels and the Alzheimer's Society research network volunteers. We are grateful to the nursing homes that supported our feasibility study. We are grateful to the Clinical research Network staff; Mary-Jo Doyle, Deborah Cooper, Kim Williams, Sally Gordon and Helen Permain and others who assisted with data collection Justin Chan, Ayesha Dar, Luiza Grycuk, Dr Adam Hughes, Dr Maria Ivanov and Angela Richardson. We would like to thank the study steering group (Dr Najma Siddiqi (Chair), Prof Tom Dening, Bert Green, Mike Bradburn, Laura Mandefield and members of the international advisory group (Prof Finbarr Martin (Chair), Prof Barbara Bowers, Prof John Gladman, Prof Claire Goodman, Prof Martin Green, Prof Raymond Koopmans, Prof Julienne Meyer, Prof Des O'Neill, Prof Jo Ouslander, Dr Hilary Rhoden, Jenny Adams, Prof Lynn Chenoweth, Prof Graham Stokes, Louise Taylor, Gavin Terry, Steve Williams). .

Competing interests statement: none declared

Funding: This work was supported by the UK NIHR grant number RP-PG-0612-20010. The views expressed are those of the author(s) and not necessarily those of the NHS, the NIHR or

the Department of Health and Social Care. The funding body had no role in the design of the study, collection, analysis, and interpretation of data and in writing the paper.

Author contributions: ELS, AF, AB, KF, RH, LM, BMcC, SN, CP, MP, GR, LR, BW-C, JY, MD were involved in development of the intervention and made substantial contributions to the concept and design of the study and protocol. LM, MP and RH analysed the data. ELS and AF wrote the paper with assistance from CP, AF, AB, KF, RH, LM, BMcC, SN, MP, GR, LR, BW-C, JY, MD critically revised the manuscript and approved the final version.

Provenance and peer review: not commissioned; externally peer reviewed

Data sharing: The datasets generated and/or analysed during the current study are not publicly available, the small number of NHs means that data may be identifiable. Data are available from the corresponding author on reasonable request.

For peer review only

REFERENCES

1. Laing and Buisson. Care of Older People UK Market Report. 28th ed, 2017.
2. Tian Y, Dixon A, Gao H. Emergency hospital admissions for ambulatory care-sensitive conditions: identifying the potential for reductions: King's Fund, 2012.
3. British Geriatric Society. Quest for quality in care homes: British Geriatrics Society Joint Working Party Inquiry into the Quality of Healthcare Support for Older People in Care Homes: A Call for Leadership, Partnership and Quality Improvement. London: British Geriatric Society, 2011.
4. Bardsley M, Blunt I, Davies S. Is secondary preventive care improving? Observational study of 10-year trends in emergency admissions for conditions amenable to ambulatory care. *BMJ Open* 2013;3(1) doi: 10.1136/bmjopen-2012- 002007
5. Kings Fund. Data briefing: emergency hospital admissions for ambulatory care sensitive conditions 2012 [Available from: https://www.kingsfund.org.uk/sites/default/files/field/field_publication_file/data-briefing-emergency-hospital-admissions-for-ambulatory-care-sensitive-conditions-apr-2012.pdf accessed 20/02/2020.
6. Mukadam N, Sampson EL. A systematic review of the prevalence, associations and outcomes of dementia in older general hospital inpatients. *IntPsychogeriatr* 2010:1-12.
7. Watkin L, Blanchard MR, Tookman A, et al. Prospective cohort study of adverse events in older people admitted to the acute general hospital: risk factors and the impact of dementia. *IntJGeriatrPsychiatry* 2012;27(1):76-82. doi: 10.1002/gps.2693 [doi]
8. Carter MW, Porell FW. Vulnerable populations at risk of potentially avoidable hospitalizations: the case of nursing home residents with Alzheimer's disease. *American journal of Alzheimer's disease and other dementias* 2005;20(6):349-58. [published Online First: 2006/01/07]
9. Grabowski DC, O'Malley AJ, Barhydt NR. The costs and potential savings associated with nursing home hospitalizations. *Health affairs (Project Hope)* 2007;26(6):1753-61. doi: 10.1377/hlthaff.26.6.1753 [published Online First: 2007/11/06]
10. Purdy S, Griffin T, Salisbury C, et al. Ambulatory care sensitive conditions: terminology and disease coding need to be more specific to aid policy makers and clinicians. *Public Health* 2009;123:169-73.
11. Walsh E, Wiener J, Haber S. Potentially avoidable hospitalizations of dually eligible Medicare and Medicaid beneficiaries from nursing facility and Home- and Community-Based Services waiver programs. *Journal of the American Geriatric Society* 2012;60(5):821-9. doi: 10.1111/j.1532-5415.2012.03920.x
12. Carter M, Porell F. Vulnerable populations at risk of potentially avoidable hospitalizations: the case of nursing home residents with Alzheimer's disease. *American Journal of Alzheimer's Disease and Other Dementias* 2005;20(6):349-58.
13. Krüger K, Jansen K, Grimsmo A. Hospital admissions from nursing homes: Rates and reasons. *Nursing Research and Practice* 2011 doi: 10.1155/2011/247623
14. Ouslander J, Diaz S, Hain D. Frequency and diagnoses associated with 7- and 30- day readmission of skilled nursing facility patients to a nonteaching community hospital. *Journal of the American Medical Directors Association* 2011;12(3):195-203. doi: 10.1016/j.jamda.2010.02.015
15. Ouslander J, Lamb G, Tappen R. Interventions to reduce hospitalizations from nursing homes: evaluation of the INTERACT II collaborative quality improvement project. *Journal of the American Geriatric Society* 2011;59(4):745-53. doi: 10.1111/j.1532-5415.2011.03333.x

For peer review only - http://bmjopen.bmj.com/site/about/guidelines.xhtml

16. Alessi C, Harker J. A prospective study of acute illness in the nursing home. *Aging* 1998;10(6):479-89.
17. Mor V, Intrator O, Fries B. Changes in hospitalization associated with introducing the Resident Assessment Instrument. *Journal of the American Geriatric Society* 1997;45(8):1002-10.
18. Young Y, Inamdar S, Dichter B. Clinical and nonclinical factors associated with potentially preventable hospitalizations among nursing home residents in New York State. *Journal of the American Medical Directors Association* 2011;12(5):364-71. doi: 10.1016/j.jamda.2010.03.006
19. Ouslander J, Perloe M, Givens J. Reducing potentially avoidable hospitalizations of nursing home residents: results of a pilot quality improvement project. *Journal of the American Medical Directors Association* 2009;10(9):644-52. doi: 10.1016/j.jamda.2009.07.001
20. Olsan T. Reducing avoidable Rehospitalisations by Improving Residents' Transitions to the Nursing Home. 2011:B22.
21. Sampson EL, Feast A, Blighe A, et al. Evidence-based intervention to reduce avoidable hospital admissions in care home residents (the Better Health in Residents in Care Homes (BHiRCH) study): protocol for a pilot cluster randomised trial. *BMJ open* 2019;9(5):e026510. doi: 10.1136/bmjopen-2018-026510 [published Online First: 2019/05/28]
22. Kitson AL, Rycroft-Malone J, Harvey G, et al. Evaluating the successful implementation of evidence into practice using the PARiHS framework: theoretical and practical challenges. *ImplementSci* 2008;3:1.
23. Schulz KF, Altman DG, Moher D, et al. CONSORT 2010 statement: updated guidelines for reporting parallel group randomised trials. *BMJ* 2010;340:c332. doi: 10.1136/bmj.c332 [published Online First: 2010/03/25]
24. Dewing J, McCormack B, Titchen A. Practice Development Workbook for Nursing, Health and Social Care Teams. West Sussex: John Wiley and Sons 2014.
25. Rycroft-Malone J, Harvey G, Seers K, et al. An exploration of the factors that influence the implementation of evidence into practice. *JClinNurs* 2004;13(8):913-24.
26. University. FA. Stop and Watch Early Warning Tool 2018 [Available from: http://www.pathway-interact.com/wp-content/uploads/2017/04/148604-Stop-and-Watch-v4_0.pdf accessed 20/02/2020.
27. Mahoney FI, Barthel DW. Functional Evaluation: The Barthel Index. *Md State Med J* 1965;14:61-5. [published Online First: 1965/02/01]
28. Edvardsson D, Fetherstonhaugh D, Nay R, et al. Development and initial testing of the Person-centered Care Assessment Tool (P-CAT). *International psychogeriatrics / IPA* 2010;22(1):101-8. doi: 10.1017/S1041610209990688 [published Online First: 2009/07/28]
29. Tjia J, Mazor KM, Field T, et al. Nurse-physician communication in the long-term care setting: perceived barriers and impact on patient safety. *J Patient Saf* 2009;5(3):145-52. doi: 10.1097/PTS.0b013e3181b53f9b [published Online First: 2009/11/21]
30. Saliba D, Kington R, Buchanan J, et al. Appropriateness of the decision to transfer nursing facility residents to the hospital. *J Am Geriatr Soc* 2000;48(2):154-63. [published Online First: 2000/02/22]
31. Beecham J, Knapp M. Costing psychiatric interventions. In: Thornicroft G, ed. Measuring Mental Helath Needs. London: Gaskell 2001:200-24.
32. Herdman M, Gudex C, Lloyd A, et al. Development and preliminary testing of the new five-level version of EQ-5D (EQ-5D-5L). *Qual Life Res* 2011;20(10):1727-36. doi: 10.1007/s11136-011-9903-x [published Online First: 2011/04/12]
33. Curtis L, Burns A. Unit Costs of Health and Social Care 2017 2017 [updated 2017. Available from: http://www.pssru.ac.uk/project-pages/unit-costs/unit-costs-2017/2018.

For peer review only - http://bmjopen.bmj.com/site/about/guidelines.xhtml

34. Joint Formulary Committee. British National Formulary. London: BMJ Group and Pharmaceutical Press 2018.
35. Department of Health. National Schedule of Reference Costs- Year 2016-2017- NHS trusts and NHS foundation trusts: NHS own cost. London, 2016.
36. Devlin NJ, Shah KK, Feng Y, et al. Valuing health-related quality of life: An EQ-5D-5L value set for England. *Health Econ* 2017 doi: 10.1002/hec.3564 [published Online First: 2017/08/24]
37. Devlin NJ, Shah K, Feng Y, et al. Valuing health-related quality of life: an EQ-5D-5L value set for England. Research Paper 16/01. London, 2016.
38. van Hout B, Janssen MF, Feng YS, et al. Interim scoring for the EQ-5D-5L: mapping the EQ-5D-5L to EQ-5D-3L value sets. *Value Health* 2012;15(5):708-15. doi: 10.1016/j.jval.2012.02.008 [published Online First: 2012/08/08]
39. National Institute for Clinical Excellence. Position statement on use of the EQ-5D-5L valuation set, 2017.
40. Briggs AH, Gray AM. Handling uncertainty when performing economic evaluation of healthcare interventions. *Health technology assessment* 1999;3(2):1-134. [published Online First: 1999/08/17]
41. Kane RL, Huckfeldt P, Tappen R, et al. Effects of an Intervention to Reduce Hospitalizations From Nursing Homes: A Randomized Implementation Trial of the INTERACT Program. *JAMA Intern Med* 2017;177(9):1257-64. doi: 10.1001/jamainternmed.2017.2657 [published Online First: 2017/07/04]
42. Gordon AL, Franklin M, Bradshaw L, et al. Health status of UK care home residents: a cohort study. *Age Ageing* 2014;43(1):97-103. doi: 10.1093/ageing/aft077
43. Saliba D, Kington R, Buchanan J, et al. Appropriateness of the decision to transfer nursing facility residents to the hospital. *Journal of the American Geriatrics Society* 2000;2000/02/22:154-63.
44. Romoren M, Gjelstad S, Lindbaek M. A structured training program for health workers in intravenous treatment with fluids and antibiotics in nursing homes: A modified stepped-wedge cluster-randomised trial to reduce hospital admissions. *PLoS One* 2017;12(9):e0182619. doi: 10.1371/journal.pone.0182619 [published Online First: 2017/09/08]
45. Van den Block L, Honinx E, Pivodic L, et al. Evaluation of a Palliative Care Program for Nursing Homes in 7 Countries: The PACE Cluster-Randomized Clinical Trial. *JAMA Intern Med* 2019:1-10. doi: 10.1001/jamainternmed.2019.5349 [published Online First: 2019/11/12]
46. Ballard C, Corbett A, Orrell M, et al. Impact of person-centred care training and person-centred activities on quality of life, agitation, and antipsychotic use in people with dementia living in nursing homes: A cluster-randomised controlled trial. *PLoS Med* 2018;15(2):e1002500. doi: 10.1371/journal.pmed.1002500 [published Online First: 2018/02/07]
47. Gordon AL, Goodman C, Davies SL, et al. Optimal healthcare delivery to care homes in the UK: a realist evaluation of what supports effective working to improve healthcare outcomes. *Age Ageing* 2018;47(4):595-603. doi: 10.1093/ageing/afx195 [published Online First: 2018/01/10]
48. Sherlaw-Johnson C, Crump H, Curry N, et al. Transforming health care in nursing homes: An evaluation of a dedicated primary care service in outer east London. London, 2018.
49. Morgan B, Hejdenberg J, Hinrichs-Krapels S, et al. Do feasibility studies contribute to, or avoid, waste in research? *PLoS One* 2018;13(4):e0195951. doi: 10.1371/journal.pone.0195951 [published Online First: 2018/04/24]

For peer review only - http://bmjopen.bmj.com/site/about/guidelines.xhtml

Figure title and legend

Figure 1 title: Resident recruitment flowchart

For peer review only

For peer review only - http://bmjopen.bmj.com/site/about/guidelines.xhtml

Figure 1. Resident recruitment flowchart

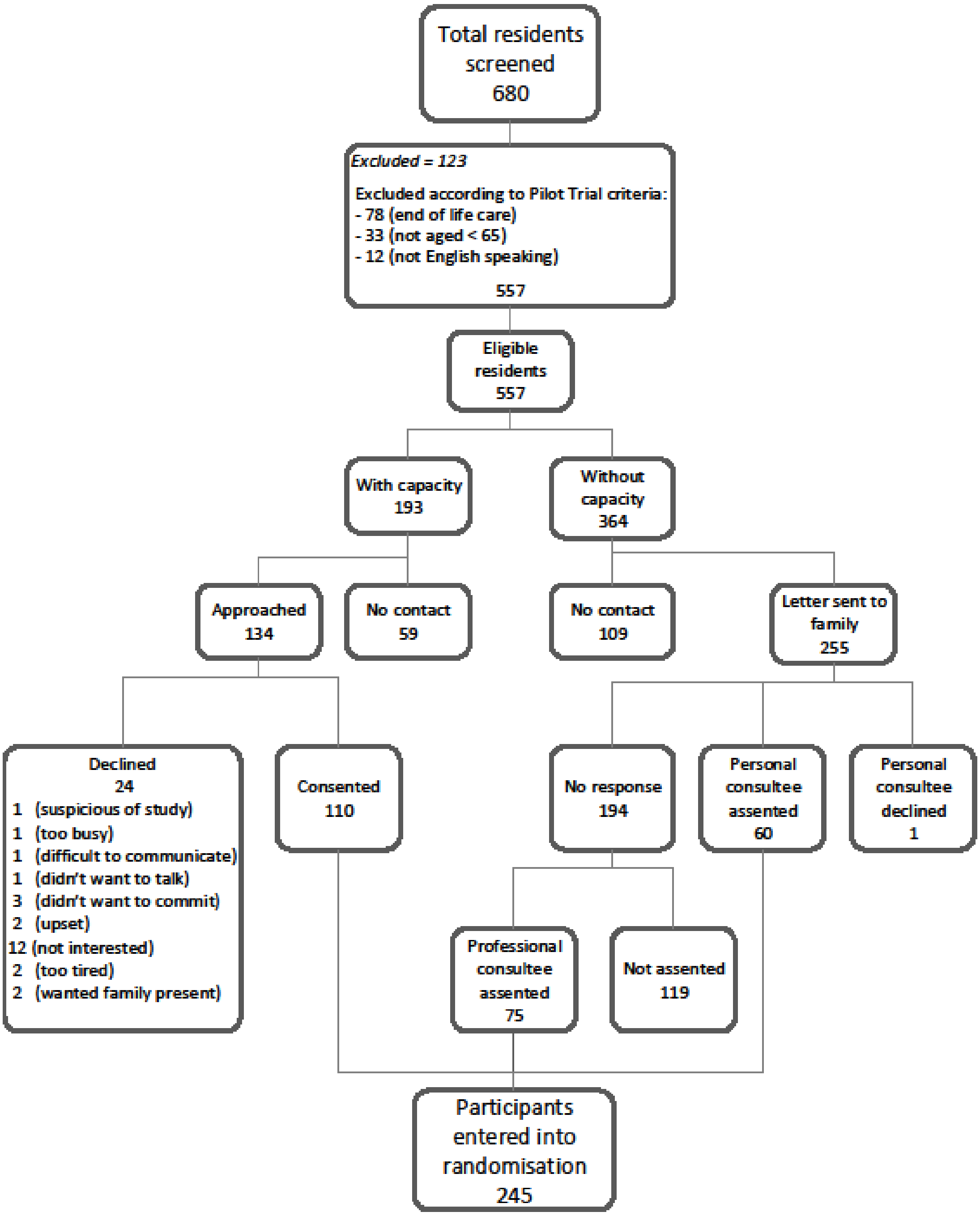

Better Health in Residents of Care Homes with Nursing - BHiRCH-NH study

Appendix 1: Pre-intervention characteristics of participating nursing homes

Characteristic	All care homes	TAU	BHiRCH-NH
	n/N or median (% or IQR)		
**Residents and beds**			
Beds available to new residents	1 (1,3)	2 (0, 2)	1 (1, 3)
Resident places	60 (36, 71)	67 (47, 84)	49 (28, 64)
Number of residents present in home	50 (34, 68)	50 (47, 83)	46 (25, 63)
Number of residents with dementia	20 (15, 33)	33 (15, 44)	20 (13, 23)
Number of residents in hospital	0 (0, 1)	1 (0, 2)	0 (0, 1)
**Medical attendances**			
Number hospital admissions	3 (2, 5)	4 (2, 7)	3 (2, 4)
Number of ambulances called	3 (2, 6)	4 (2, 9)	3 (2, 6)
Unscheduled (out of hours) GP visits or telephone contacts	1 (1, 3)	2 (1, 3)	1 (1, 3)
Accident and Emergency attendances	3 (2, 5)	3 (1, 4)	3 (2, 6)
**Staffing**			
Qualified nursing staff rostered on during the day	3 (2, 5)	3 (2, 5)	3 (2, 5)
Care staff rostered on during the day	11 (9, 13)	11 (9, 14)	12 (6, 13)
Qualified nursing staff rostered on during the night	2 (1, 3)	2 (1, 3)	2 (1, 3)
Care staff rostered on during the night	3 (3, 7)	3 (3, 7)	4 (2, 7)
Number of agency/ bank staff in 24 hour period	1 (0, 3)	1 (0, 3)	1 (0, 3)
Number of permanent registered nursing staff (including those on sick/carer/compassionate leave)	10 (8, 13)	11 (10, 13)	8 (7, 15)
Number of permanent other care staff (including those on sick/carer/compassionate leave)	26 (24, 57)	26 (24, 70)	33 (23, 57)
Number of registered nursing staff from those above on sick/carer/ compassionate leave	0 (0, 1)	0 (0, 1)	0 (0, 1)
Number of other care staff from those above on sick/carer/ compassionate leave	0 (0, 2)	1 (0, 6)	0 (0, 2)
**Nursing home**			
Privately managed	12/13 (92)	6/7 (86)	6/6 (100)
Local Authority managed	1/13 (8)	1/7 (14)	0/6 (0)
Nursing	6/13 (46)	4/7 (57)	2/6 (33)
Nursing and personal care	7/13 (54)	3/7 (43)	4/6 (67)
Dementia registered	8/11 (73)	5/6 (83)	3/5 (60)
Dementia specialist	2/12 (17)	2/6 (33)	0/6 (0)
**Regular nursing home attendance from**			
Physiotherapist	7/12* (58)	4/7 (57)	3/5 (60)
Geriatrician	3/12 (25)	2/7 (29)	1/5 (20)
District Nurse	5/13 (38)	3/7 (43)	2/6 (33)
Tissue viability nurses	7/12 (58)	3/7 (43)	4/5 (80)
Dietitian	9/13 (69)	4/7 (57)	5/6 (83)
Speech and language therapist	8/13 (62)	4/7 (57)	4/6 (67)
Optician	11/13 (85)	5/7 (71)	6/6 (100)
Ophthalmologist	4/13 (31)	1/7 (14)	3/6 (50)
Chiropodist	13/13 (100)	7/7 (100)	6/6 (100)
Occupational therapist	7/13 (54)	3/7 (43)	4/6 (67)
Dentist	8/12 (67)	5/7 (71)	3/5 (60)
Audiologist	2/12 (17)	1/7 (14)	1/5 (20)
Dedicated GP for all residents	13/13 (100)	7/7 (100)	6/6 (100)

***for some variables N=12 where this data was not available for a particular home**

For peer review only

Appendix 2. Family carer recruitment and retention

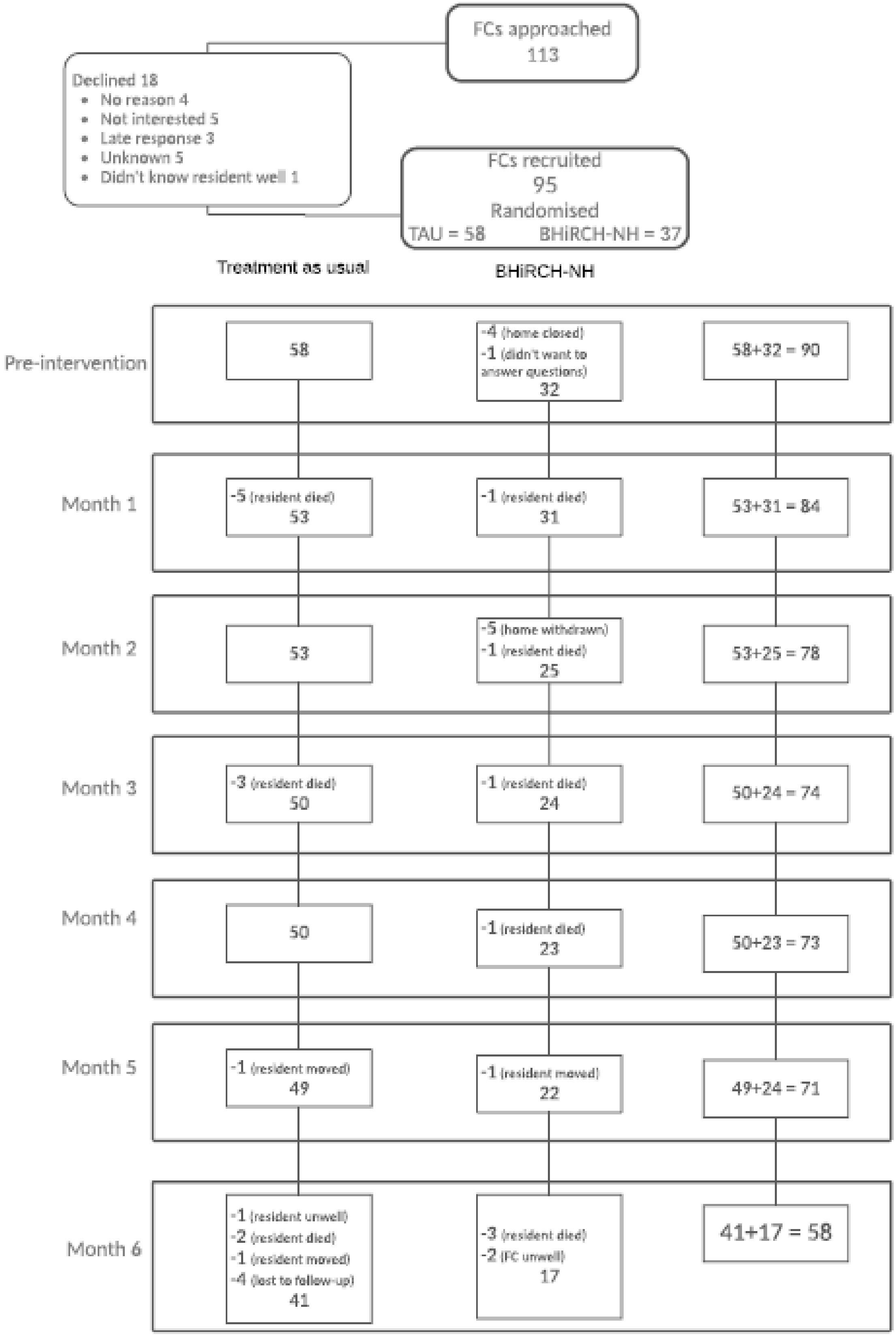

Appendix 3. Staff recruitment and retention flowchart

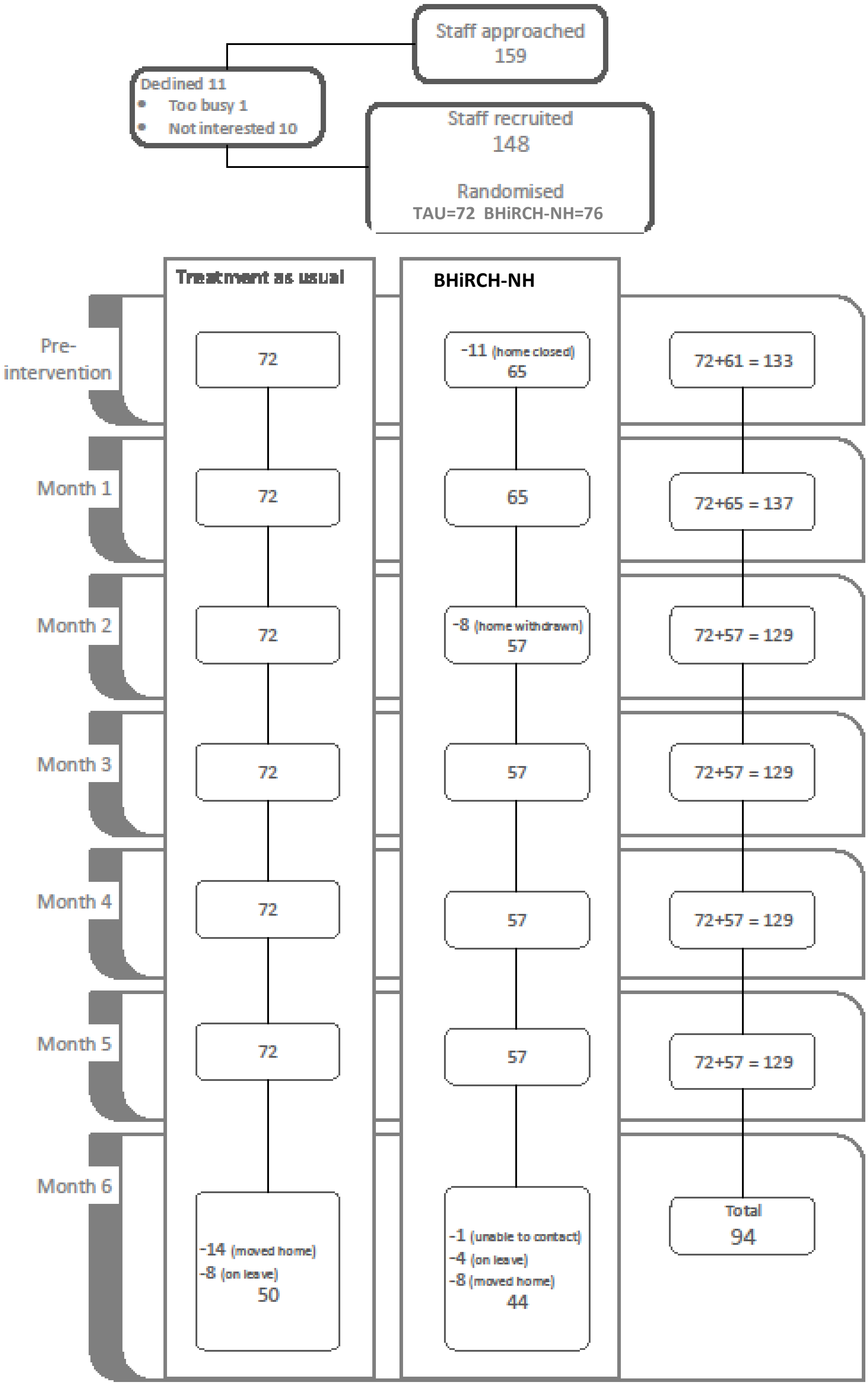

Better Health in Residents of Care Homes with Nursing - BHiRCH-NH study

Appendix 4: Staff participant characteristics pre-intervention

Characteristic	Cohort	TAU	Intervention
	n/N or median (% or IQR)		
**Demographics**	N=130	N=70	N=60
Male	16 (12)	9 (13)	7 (12)
Age	42 (30, 53)	42(32, 53)	39 (27, 50)
**Ethnicity**	N=129	N=69	N=60
White	76 (59)	32 (46)	44 (73)
Black	29 (22)	21 (30)	8 (13)
Asian	16 (12)	9 (13)	7 (12)
Other	8 (16)	7 (10)	1 (2)
**Marital status**	N=129	N=69	N=60
Married or cohabiting	71 (55)	40 (58)	31 (52)
Single	47 (36)	22 (32)	25 (42)
Divorced or widowed	11 (9)	7 (10)	4 (7)
**Education**	N=128	N=69	N=59
Completed years of education	11(11, 12)	12 (11, 12)	11 (11, 12)
No qualifications or GCSE or equivalent	16 (13)	9 (13)	7 (12)
A Level/NVQ/ HNC/ HND or equivalent	46 (36)	18 (26)	28 (47)
Degree or higher degree	49 (38)	28 (41)	21 (36)
Other qualification	17 (13)	14 (20)	3 (5)
**Type of staff**	N=125	N=67	N=58
Registered Nurse	24 (19)	9 (13)	7 (12)
Manager/ Matron/ Charge Nurse	12 (10)	8 (12)	4 (7)
Staff Nurse	2 (2)	5 (7)	3 (5)
Agency Nurse	87 (70)	1 (1)	1 (2)
Not a nurse		44 (66)	43 (74)
**Nurses only**	N=40	N=24	N=16
Works days	28 (70)	16 (67)	12 (75)
Works evenings	2 (5)	1 (4)	1 (6)
Works nights	9 (23)	4 (17)	5 (31)
Works mornings/early 12-hour shifts	12 (30)	5 (21)	7 (44)
Works afternoons/late 12-hour shifts	3 (8)	0 (0)	3 (19)
Works weekends	3 (8)	1 (4)	2 (13)
**Number of hours/week working in this nursing home**	N=40	N=24	N=16
Less than 8	1 (3)	0 (0)	1 (6)
8 to 12	0 (0)	0 (0)	0 (0)
13 to 40	13 (33)	8 (33)	5 (31)
More than 40	26 (65)	16 (67)	10 (63)
**How long have you worked at this nursing home?**	N=40	N=24	N=16
Less than 2 months	1 (3)	1 (4)	0
2 to 6 months	4 (10)	3 (13)	1 (6)
7 to 11 months	7 (18)	4 (17)	3 (19)
1 to 5 years	17 (43)	7 (29)	10 (63)
6 to 10 years	6 (15)	6 (25)	0
More than 10 years	5 (13)	3 (13)	2 (13)
**How long have you worked in nursing homes?**	N=39	N=24	N=15
7 to 11 months	2 (5)	1 (4)	1 (7)
1 to 5 years	14 (36)	6 (25)	8 (53)
6 to 10 years	7 (18)	7 (29)	0
More than 10 years	16 (41)	10 (42)	6 (40)
**How long have you been a registered nurse?**	N=40	N=24	N=16
1 to 5 years	8 (20)	3 (13)	5 (31)
6 to 10 years	10 (25)	7 (29)	3 (19)
More than 10 years	22 (55)	14 (58)	8 (50)

For peer review only

Better Health in Residents of Care Homes with Nursing - BHiRCH-NH study

**English is first language**	20 (50)	12 (50)	8 (50)
**PCAT Score**	49 (I46, 53)	50 (47,54)	48 (44,51)

For peer review only

Appendix 5: Nurse – General Practitioner Communication Tool scores

	Pre-intervention			6 month follow up	
	Cohort	TAU	BHiRCH-NH<br>n/N (%)	TAU	BHiRCH-NH
Have difficulty understanding what GP means due to medical jargon	2/40 (5)	2/24 (8)	0/16 (0)	0/14 (0)	0/7 (0)
A GP's language or accent makes it hard for you to understand what they are saying	2/40 (5)	1/24 (4)	1/16 (16)	0/15 (0)	0/7 (0)
A GP has difficulty understanding what you are saying due to your language or accent	2/40 (5)	2/24 (8)	0/16 (0)	0/15 (0)	0/7 (0)
GPs interrupt you before you have finished reporting on a resident	4/40 (10)	2/24 (8)	2/16 (13)	1/14 (7)	0/7 0)
GPs consider nurses' views when making decisions about residents	29/39 (10)	18/23 (78)	11/16 (69)	12/15 (80)	5/7 (71)
GPs are rude to you when you call them about a resident	2/39 (5)	2/23 (9)	0/16 (0)	1/15 (7)	0/7 (0)
You feel respected after an interaction with a GP?	29/40 (73)	19/24 (79)	10/16 (63)	12/15 (80)	4/7 (57)
You feel frustrated after an interaction with a GP?	4/40 (10)	3/24 (13)	1/16 (6)	1/15 (7)	0/7 (0)
Difficulty reaching the GP	9/40 (23)	5/24 (21)	4/16 (25)	2/15 (13)	2/7 (29)
Uncertainty about what to tell the GP	1/38 (3)	1/23 (4)	0/15 (0)	0/15 (0)	0/7 (0)
Feeling that the GP doesn't want to deal with the problem	3/40 (8)	1/24 (4)	2/16 (13)	1/15 (7)	1/7 (14)
Finding time to make the call	11/40 (28)	8/24 (33)	3/16 (19)	3/15 (20)	2/7 (29)
Finding a quiet place to make the call	10/40 (25)	7/24 (29)	3/16 (19)	4/14 (29)	0/7 (0)
Anticipating that the GP will be rude or unpleasant	1/40 (3)	0/24 (0)	1/16 (6)	1/15 (7)	0/7 (0)
Feeling hurried by the GP	7/40 (18)	4/24 (17)	3/16 (19)	1/15 (7)	0/7 (0)
Feeling that I am bothering the GP	4/39 (10)	2/24 (8)	2/15 (13)	2/15 (13)	0/7 (0)
Worrying that the GP may order something inappropriate or unnecessary	4/40 (10)	2/24 (8)	2/16 (13)	1/15 (7)	0/7 (0)
Feeling that I don't have enough time to say everything that I need to say	3/39 (8)	1/24 (4)	2/15 (13)	1/15 (7)	0/7 (0)
Nurses get good training in this care home	28/39 (72)	16/24 (67)	12/15 (80)	14/15 (93)	5/7 (71)
Nurses in this care home are willing to try new protocols	33/39 (85)	20/24 (83)	13/15 (87)	11/15 (73)	5/7 (71)
Nurses in this care home sometimes have to ignore protocols to get everything done	6/39 (15)	4/24 (17)	2/15 (13)	3/15 (20)	1/7 (14)
When this care home makes changes to improve resident care, they follow up to see if the changes worked	31/39 (79)	18/24 (75)	13/15 (87)	15/15 (100)	6/7 (86)
It is hard to make changes to improve resident care in this care home	4/39 (10)	1/24 (4)	3/15 (20)	2/15 (13)	0/7 (0)
This care home often wants nurses to follow protocols that don't really help residents	3/39 (8)	1/24 (4)	2/15 (13)	2/15 (13)	0/7 (0)
New protocols often make it harder for nurses to do their job	4/39 (10)	2/24 (8)	2/15 (13)	4/15 (27)	0/7 (0)
The communication between nurses and GPs in this care home is open	34/40 (85)	20/24 (83)	14/16 (88)	13/15 (87)	7/7 (100)
Feel comfortable communicating with GPs?	36/40 (90)	23/24 (96)	13/16 (81)	15/15 (100)	7/7 (100)
Feel comfortable communicating with nurse practitioners?	34/40 (85)	23/24 (96)	11/16 (69)	14/15 (93)	6/6 (100)

For peer review only

Better Health in Residents of Care Homes with Nursing - BHiRCH-NH study

Appendix 6: Nurse self- assessed core competencies

Core competencies	Pre-intervention			6 month follow up	
	Cohort	TAU	BHiRCH-NH	TAU	BHiRCH-NH
			n/N (%)		
**I know how acute episodes of chronic heart failure may manifest in older people**					
Disagree	0/34 (0)	0/21 (0)	0/13 (0)	0/15 (0)	0/7 (0)
Neither agree nor disagree	2/34 (6)	0/21 (0)	2/13 (15)	1/15 (7)	0/7 (0)
Agree	18/34 (53)	12/21 (57)	6/13 (46)	7/15 (47)	4/7 (57)
Completely agree	14/34 (41)	9/21 (43)	5/13 (38)	7/15 (47)	3/7 (43)
**I know how respiratory infections may manifest in older people**					
Disagree	0/34 (0)	0/21 (0)	0/13 (0)	0/15 (0)	0/7 (0)
Neither agree nor disagree	0/34 (0)	0/21 (0)	0/13 (0)	0/15 (0)	0/7 (0)
Agree	13/34 (38)	7/21 (33)	6/13 (46)	6/15 (40)	3/7 (43)
Completely agree	21/34 (62)	14/21 (67)	7/13 (54)	9/15 (60)	4/7 (57)
**I know how dehydration may manifest in older people**					
Disagree	0/34 (0)	0/21(0)	0/13 (0)	0/15 (0)	0/7 (0)
Neither agree nor disagree	0/34 (0)	0/21 (0)	0/13 (0)	0/15(0)	0/7 V
Agree	14/34 (41)	7/21 (33)	7/13 (54)	5/15 (33)	1/7 (14)
Completely agree	20/34 (59)	14/21 (67)	6/13 (46)	10/15 (67)	6/7 (86)
**I know how urinary tract infections may manifest in older people**					
Disagree	0/34 (0)	0/21 (0)	0/13 (0)	0/15 (0)	0/7 (0)
Neither agree nor disagree	0/34 (0)	0/21 (0)	0/13 (0)	0/15 (0)	0/7 (0)
Agree	15/3 4 (44)	9/21 (43)	6/13 (46)	5/15	1/7 (14)
Completely agree	19/34 (56)	12/21 (57)	7/13 (54)	10/15 (67)	6/7 (86)
**I am able to identify changes in physiology or behaviour as compared to baseline or normal for that resident**					
Disagree	0/34 (0)	0/21 (0)	0/13 (0)	0/15 (0)	0/7 (0)
Neither agree nor disagree	0/34 (0)	0/21 (0)	0/13 (0)	0/15 (0)	0/7 (0)
Agree	14/34 (41)	8/21(38)	6/13 (46)	5/15 (33)	0/7 (0)
Completely agree	20/34 (59)	13/21 (62)	7/13 (54)	10/15 (67)	7/7 (100)
**I am able to identify changes in physiology or behaviour for people who cannot communicate verbally**					
Disagree	0/34 (0)	0/21	0/13 (0)	0/15 (0)	0/7 (0)
Neither agree nor disagree	1/34 (3)	0/21(0)	1/13 (8)	1/15 (7)	0/7 (0)
Agree	17/34 (50)	11/21 (52)	6/13 (46)	6/15 (40)	2/7 (29)
Completely agree	16/34 (47)	10/21 (48)	6/13 (46)	8/15 (53)	5/7 (71)
**I know each resident's existing medical conditions**					
Disagree	1/34 (3)	0/21 (0)	1/13 (8)	0/15 (0)	1/7 (14)
Neither agree nor disagree	2/34 (6)	1/21 (5)	1/13 (8)	1/15 (7)	0/7 (0)
Agree	22/34 (65)	15/21 (71)	7/13 (54)	8/15 (53)	6/7 (86)
Completely agree	9/34 (26)	5/21 (24)	4/13 (31)	6/15 (40)	0/7 (0)
**I know each resident's care plans with respect to these medical conditions**					
Disagree	1/34 (3)	0/21 (0)	1/13 (8)	0/15 (0)	0/7(0)
Neither agree nor disagree	2/34 (6)	1/21 (5)	1/13 (8)	1/15 (7)	1/7 (14)
Agree	17/34 (50	10/21 (48)	7/13 (54)	8/15 (53)	5/7 (71)

For peer review only

Completely agree	14/34 (41)	10/21 (48)	4/13 (31)	6/15 (40)	1/7 (14)
**I know what is normal (i.e. their baseline) for each resident in terms of physiology, abilities and behaviour**					
Disagree	0/34 (0)	0/21 (0)	0/13 (0)	0/15 (0)	0/7 (0)
Neither agree nor disagree	1/34 (3)	1/21 (5)	0/13 (0)	2/15 (13)	0/7(0)
Agree	19/34 (56)	11/21 (52)	8/13 (62)	7/15 (47)	6/7 (86)
Completely agree	14/34 (41)	9/21 (43)	5/13 (38)	6/15 (40)	1/7 (14)
**I am able to carry out immediate interventions (eg adjusting fluid levels)**					
Disagree	1/33 (3)	1/21 (5)	0/12 (0)	0/15 (0)	0/7 (0)
Neither agree nor disagree	0/33 (0)	0/21 (0)	0/12 (0)	0/15 (0)	1/7 (14)
Agree	21/33 (64)	10/21 (48)	11/12 (92)	6/15 (40)	6/7(86)
Completely agree	11/33 (33)	10/21 (48)	1/12 (8)	9/15 (60)	0/7 (0)
**I am able to recognise verbal and nonverbal signs of acute deterioration**					
Disagree	0/34 (0)	0/21 (0)	0/13 (0)	0/15 (0)	0/7 (0)
Neither agree nor disagree	0/34 (0)	0/21 (0)	0/13 (0)	1/15 (7)	0/7 (0)
Agree	19/34 (56)	10/21 (48)	9/13 (69)	6/15 (40)	4/7 (57)
Completely agree	15/34 (44)	11/21 (52)	4/13 (31)	8/15 (53)	3/7 (43)
**I am able to carry out bedside observations (vital signs etc)**					
Disagree	0/34 (0)	0/21 (0)	0/13 (0)	0/15 (0)	0/7 (0)
Neither agree nor disagree	0/34 (0)	0/21 (0)	0/13 (0)	0/15 (0)	0/7 (0)
Agree	11/34 (32)	6/21 (29)	5/13 (38)	5/15 (33)	4/7 (57)
Completely agree	23/34 (68)	15/21 (71)	8/13 (62)	10/15(67)	3/7 (43)
**I am able to utilise basic clinical skills (use of medication etc)**					
Disagree	0/34 (0)	0/21 (0)	0/13 (0)	0/15 (0)	0/7 (0)
Neither agree nor disagree	0/34 (0)	0/21 (0)	0/13 (0)	0/15 (0)	0/7 (0)
Agree	11/34 (32)	5/21 (24)	6/13	5/15	3/7
Completely agree	23/34 (68)	16/21 (76)	7/13 (54)	10/15 (67)	4/7 (57)
I **know when to seek additional support (eg. asking a nurse, calling a GP or ambulance)**					
Disagree	0/34 (0)	0/21 (0)	0/13 (0)	0/15 (0)	0/7 (0)
Neither agree nor disagree	0/34 (0)	0/21 (0)	0/13 (0)	0/15 (0)	0/7 (0)
Agree	10/34 (29)	4/21 (19)	6/13 (46)	4/15 (27)	2/7 (29)
Completely agree	24/34 (71)	17/21 (81)	7/13 (54)	11/15 (73)	5/7 (71)
**I am able to set and monitor staff work to ensure quality of health care is provided**					
Disagree	1/34 (3)	1/21 (5)	0/13 (0)	1/15 (7)	0/7(0)
Neither agree nor disagree	0/34 (0)	0/21 V	0/13 V	0/15 (0)	0/7 (0)
Agree	14/34 (41)	5/21 (24)	9/13 (69)	4/15 (27)	3/7 (43)
Completely agree	19/34 (56)	15/21 (71)	4/13 (31)	10/15 (67)	4/7 (57)
**I am able to encourage or support other staff in their care for acute deterioration in resident's health**					
Disagree	0/34 (0)	0/21 V	0/13 (0)	0/15 (0)	0/7 (0)
Neither agree nor disagree	2/34 (6)	2/21 (10)	0/13 (0)	0/15 (0)	0/7 (0)
Agree	14/34 (41)	5/21 (24)	9/13 (69)	6/15 (40)	3/ (43)7
Completely agree	18/34 (53)	14/21 (67)	4/13 (31)	9/15 (60)	4/7 (57)
**I am able to negotiate and plan a course of action with other staff**					
Disagree	0/34 (0)	0/21 (0)	0/13 (0)	0/15 (0)	0/7 (0)
Neither agree nor disagree	4/34 (12)	3/21 (14)	1/13 (8)	1/15 (7)	0/7 (0)
Agree	13/34 (38)	6/21 (29)	7/13 (54)	5/15 (33)	5/7 (71)

For peer review only

Completely agree	17/34 (50)	12/21 (57)	5/13 (38)	9/15 (60)	2/7 (29)
**I am able to record a plan of action, e.g. draft and update care plans**					
Disagree	0/34 (0)	0/21 (0)	0/13 (0)	0/15 (0)	0/ (0)7
Neither agree nor disagree	0/34 (0)	0/21 (0)	0/13 (0)	1/15 (7)	0/7 (0)
Agree	16/34 (47)	10/21 (48)	6/13 (46)	4/15 (27)	3/7 (43)
Completely agree	18/34 (53)	11/21 (52)	7/13 (54)	10/15 (67)	4/7 (57)
**I am able to ensure acute changes in a resident's health status are communicated to staff in written or oral form**					
Disagree	0/34 (0)	0/21 (0)	0/13 (0)	0/15 (0)	0/7 (0)
Neither agree nor disagree	0/34 (0)	0/21 (0)	0/13 (0)	1/15 (7)	0/7(0)
Agree	14/34 (41)	7/21 (33)	7/13 (54)	5/15 (33)	3/7 (43)
Completely agree	20/34 (59)	14/21 (67)	6/13 (46)	9/15 (60)	4/7 (57)
**I am able to ensure effectiveness of treatment for acute changes in a resident's health status are communicated to staff and carers in written or oral form**					
Disagree	0/34 (0)	0/21 (0)	0/13 (0)	0/15 (0)	0/7 (0)
Neither agree nor disagree	2/34 (6)	1/21 (5)	1/13 (8)	1/15 (7)	0/7 (0)
Agree	12/34 (35)	6/21 (29)	6/13 (46)	6/15 (40)	2/7 (29)
Completely agree	20/34 (59)	14/21 (67)	6/13 (46)	8/15 (53)	5/7 (71)
**I am able to convey information about changes in health accurately and sensitively to carers**					
Disagree	0/34 (0)	0/21 (0)	0/13 (0)	0/15 (0)	0/6 (0)
Neither agree nor disagree	1/34 (3)	0/21 (0)	1/13 (8)	1/15 (7)	0/6 (0)
Agree	16/34 (47)	10/21 (48)	6/13 (46)	6/15 (40)	2/6 (33)
Completely agree	17/34 (50)	11/21 (52)	6/13 (46)	8/15 (53)	4/6 (67)
**I am able to adapt assessment and communication for people with dementia or communication difficulties**					
Disagree	1/34 (3)	1/21 (5)	0/13 (0)	0/15 (0)	0/7 (0)
Neither agree nor disagree	1/34 (3)	1/21 (5)	0/13 (0)	1/15 (7)	0/7 (0)
Agree	17/34 (50)	11/21 (52)	6/13 (460	6/15 (40)	3/7 (43)
Completely agree	15/34 (44)	8/21 (38)	7/13 (54)	8/15 (53)	4/7 (57)
**I am able to communicate the nature of change in a resident's health status**					
Disagree	0/34 (0)	0/21 (0)	0/13 (0)	0/15 (0)	0/7 (0)
Neither agree nor disagree	0/34 (0)	0/21 (0)	0/13 (0)	1/15 (7)	0/7 (0)
Agree	17/34 (50)	12/21 (57)	5/13 (38)	5/15 (33)	3/7 (43)
Completely agree	17/34 (50)	9/21 (43)	8/13 (62)	9/15 (60)	4/7 (57)

Better Health in Residents of Care Homes with Nursing - BHiRCH-NH study

Appendix 7. Serious adverse events categorised by type and potential relationship to the intervention

Type of serious adverse event (SAE)	TAU N=55* n/N (%)	Intervention N=41* n/N (%)
**Type of SAE**		
Death	17 (31)	14 (34)
Requires hospitalisation or prolonged hospital stay	37 (67)	25 (61)
Other medically significant	1 (2)	2 (5)
**"Expectedness" of SAE**		
SAE is expected side effect or possible outcome of the intervention as defined in the protocol (for example admission to hospital)	55 (100)	39 (95)
SAE is not expected	0 (0)	
Hernia		1 (2)
Choking		1 (2)
**Cause of SAE**		
SAE found by independent trial steering group not directly related to the intervention	55 (100)	41 (100)

Table footnote:

*denominator is number of serious adverse events not residents

†Serious adverse event is defined as per the UK Health Research Authority standard definition "an untoward occurrence that:

(a) results in death;

(b) is life-threatening;

(c) requires hospitalisation or prolongation of existing hospitalisation;

(d) results in persistent or significant disability or incapacity; and

(e) consists of a congenital anomaly or birth defect.

Appendix 8. Resident retention flowchart

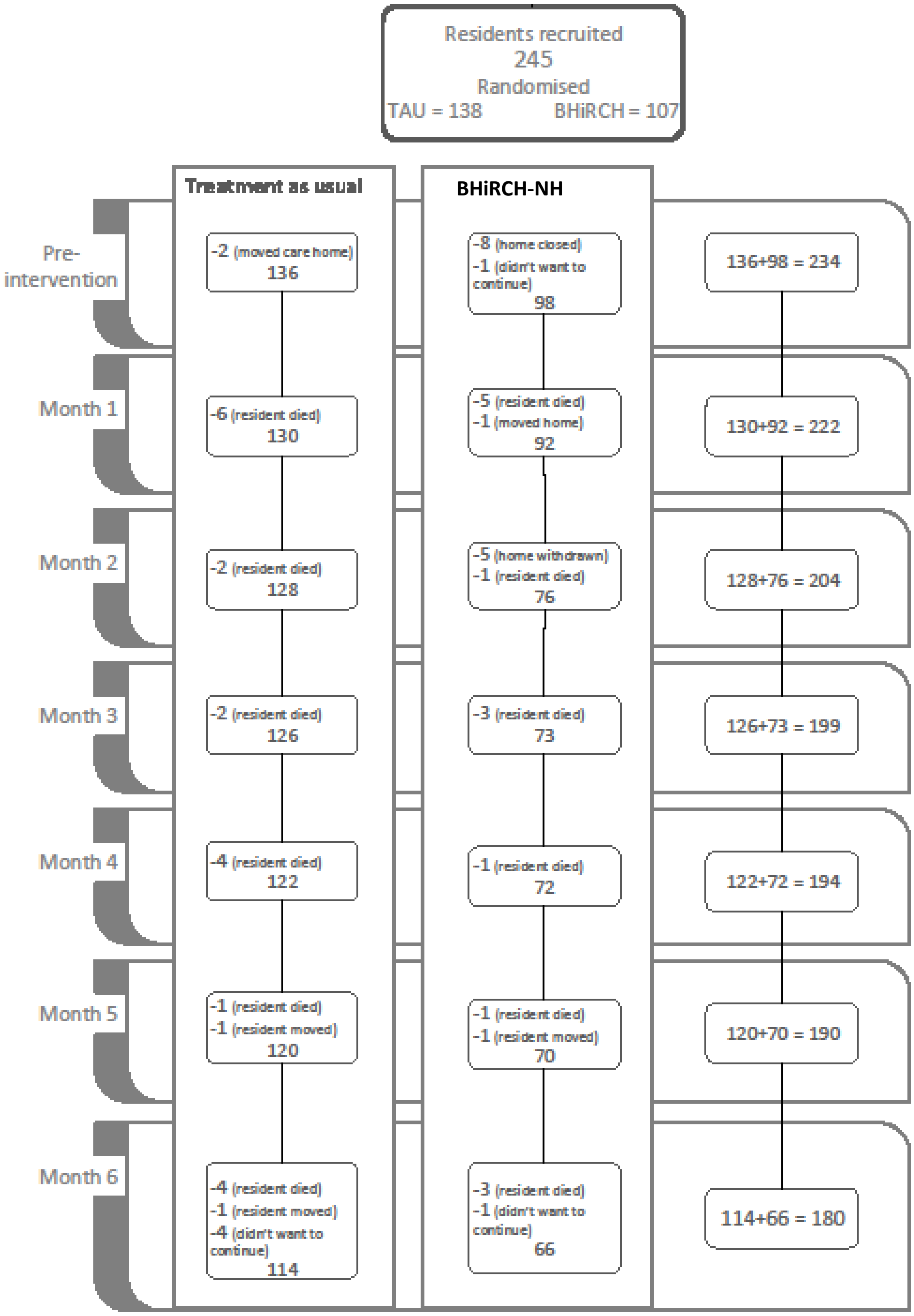

Better Health in Residents of Care Homes with Nursing - BHiRCH-NH study

Appendix 9. Mean healthcare resource use ± standard deviation (SD) and cost (unit costs at 2016/2017 values) per resident over the 6 months, complete data

Type of resource use (unit)	TAU	BHiRCH-NH	Difference (BHiRCH-NH vs TAU)		TAU	BHiRCH-NH	Incremental difference (BHiRCH-NH vs TAU)
	Mean ± SD	Mean ± SD	Mean	p-value	Mean cost	Mean cost	Mean cost (95% CI)
**Primary care, community health or emergency services (visits)**	8.3 ± 6.1 *(n=114)*	9.6 ± 7.6 *(n=71)*	1.3	0.20	£491.3	£694.9	£203.6 (-£67.4 to £474.6)
**Outpatient appointments (visits)**	0.4 ± 1.0 *(n=114)*	0.7 ± 1.5 *(n=68)*	0.3	0.11	£22.6	£49.9	£27.4 (£1.2 to £53.5)
**Inpatient services (admissions)**	0.2 ± 0.5 *(n=114)*	0.2 ± 0.6 *(n=68)*	0.001	0.99	£356.9	£426.6	£69.6 (-£333.8 to £472.9)
**Ambulance service (calls)**	0.2 ± 0.6 *(n=114)*	0.2 ± 0.5 *(n=68)*	0.04	0.61	£1.3	£1.6	£0.3 (-£0.9 to £1.4)
**Accident and Emergency (attendances)**	0.1 ± 0.5 *(n=114)*	0.3 ± 0.6 *(n=68)*	0.1	0.21	£23.1	£38.7	£15.6 (-£8.9 to £40.1)
**Prescriptions (packages)**	54.1 ± 23.8 *(n=114)*	60.5 ± 26.6 *(n=68)*	6.33	0.10	£325.7	£381.6	£55.9 (-£43.8 to £155.6)
**Length of stay in hospital (days)**	0.8 ± 3.9 *(n=114)*	0.9 ± 3.9 *(n=68)*	0.2	0.76			

For peer review only

Better Health in Residents of Care Homes with Nursing - BHiRCH-NH study

Appendix 10. Mean utility values and QALYs per resident (based on resident self-completed EQ-5D-5L questionnaire)

Utility values	TAU		BHiRCH-NH		Incremental difference	
	Mean	(95%CI)	Mean	(95%CI)	Mean	p-value
**Complete cases**						
Baseline	0.610 *(n=69)*	0.540 to 0.680	0.487 *(n=58)*	0.403 to 0.570	-0.123	0.02
6 months	0.685 *(n=59)*	0.610 to 0.759	0.614 *(n=32)*	0.501 to 0.727	-0.071	0.28
**QALYs**	0.337 *(n=46)*	0.297 to 0.377	0.259 *(n=26)*	0.196 to 0.321	-0.078	0.03
**With imputations**						
Baseline	0.617	0.607 to 0.628	0.504	0.491 to 0.518	-0.113	<0.001
6 months	0.652	0.641 to 0.662	0.649	0.637 to 0.661	-0.003	0.73
**QALYs**	0.317	0.312 to 0.322	0.289	0.283 to 0.294	-0.029	<0.001

For peer review only

Appendix 11. Mean cost ± standard deviation (SD) of BHiRCH-NH and TAU of resource use per resident over 6 months

Type of costs	TAU	BHiRCH-NH	BHiRCH-NH vs. TAU
**BHiRCH-NH intervention costs per resident**			
Training	£0 ± £0	£39 ± £28	£39
Materials used in training	£0 ± £0	£0.20 ± £0.10	£0.20
Delivery of the intervention	£0 ± £0	£34 ± £32	£34
**Total costs (95%CI) per resident of healthcare resource use over 6 months**			
Complete cases	£1,250 (£900 to £1,599) *(n=105)*	£1,560 (£912 to £2,209) *(n=66)*	£310 (-£361 to £981) *(n=171)*
With imputation	£1,233 (£1,171 to £1,295)	£1,458 (£1,351 to £1,566)	£225 (£108 to £342)
**Total costs (95%CI) per resident of BHiRCH-NH intervention cost + healthcare resource use over 6 months**			
Complete cases	£1,250 (£900 to £1,599) *(n=105)*	£1,640 (£989 to £2,291) *(n=66)*	£390 (-£283 to £1,063) *(n=171)*
With imputation	£1,233 (£1,171 to £1,295)	£1,532 (£1,424 to £1,640)	£299 (£182 to £417)

For peer review only

Appendix 12. Cost-effectiveness acceptability curve showing the probability that BHiRCH-NH intervention is cost-effective versus TAU at different values of the WTP for a QALY, N=237

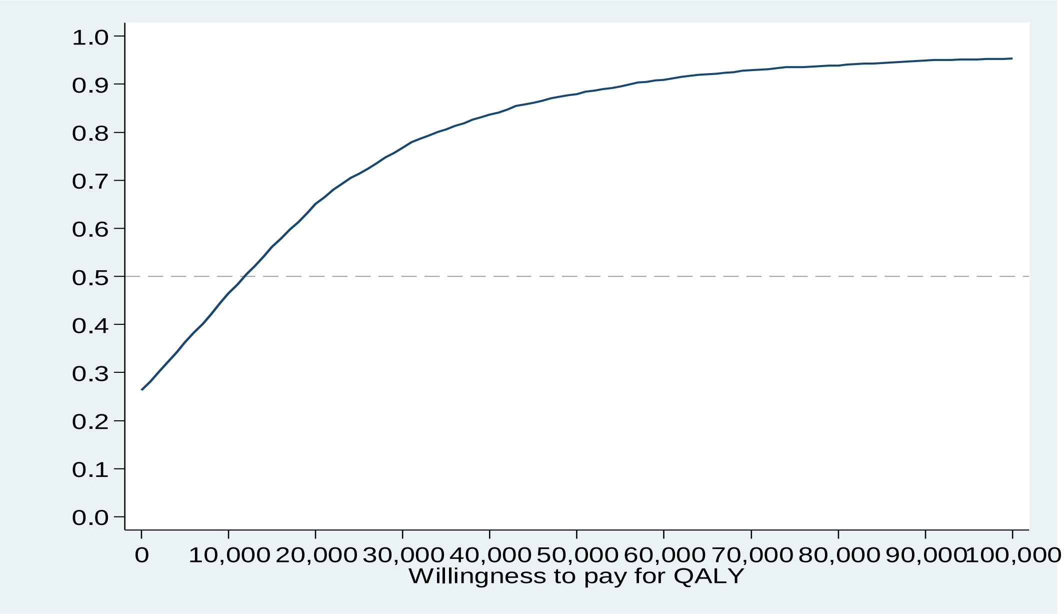

Figure 5 legend: Abbreviations: BHiRCH-NH-Better Health in Residents of Care Homes with Nursing study, QALY- Quality Adjusted Life year, TAU-Treatment as usual, WTP-Willingness to Pay

review only

For peer review only - http://bmjopen.bmj.com/site/about/guidelines.xhtml

CONSORT 2010 checklist of information to include when reporting a randomised trial*

Section/Topic	Item No	Checklist item	Reported on page No
**Title and abstract**			
	1a	Identification as a randomised trial in the title	Title page
	1b	Structured summary of trial design, methods, results, and conclusions (for specific guidance see CONSORT for abstracts)	Page 1
**Introduction**			
Background and objectives	2a	Scientific background and explanation of rationale	Page 4-5
	2b	Specific objectives or hypotheses	Page 5
**Methods**			
Trial design	3a	Description of trial design (such as parallel, factorial) including allocation ratio	Page 5
	3b	Important changes to methods after trial commencement (such as eligibility criteria), with reasons	Page 8
Participants	4a	Eligibility criteria for participants	Pages 6-7
	4b	Settings and locations where the data were collected	Pages 6-7
Interventions	5	The interventions for each group with sufficient details to allow replication, including how and when they were actually administered	Pages 8-9
Outcomes	6a	Completely defined pre-specified primary and secondary outcome measures, including how and when they were assessed	Pages 12-13
	6b	Any changes to trial outcomes after the trial commenced, with reasons	Page 8
Sample size	7a	How sample size was determined	Page 14
	7b	When applicable, explanation of any interim analyses and stopping guidelines	N/A
Randomisation:			
Sequence generation	8a	Method used to generate the random allocation sequence	Page 14
	8b	Type of randomisation; details of any restriction (such as blocking and block size)	Page 14
Allocation concealment mechanism	9	Mechanism used to implement the random allocation sequence (such as sequentially numbered containers), describing any steps taken to conceal the sequence until interventions were assigned	Page 14
Implementation	10	Who generated the random allocation sequence, who enrolled participants, and who assigned participants to interventions	Page 14
Blinding	11a	If done, who was blinded after assignment to interventions (for example, participants, care providers, those	Page 14

For peer review only

		assessing outcomes) and how	
	11b	If relevant, description of the similarity of interventions	N/A
Statistical methods	12a	Statistical methods used to compare groups for primary and secondary outcomes	Page 14-15
	12b	Methods for additional analyses, such as subgroup analyses and adjusted analyses	Page 15
**Results**			
Participant flow (a diagram is strongly recommended)	13a	For each group, the numbers of participants who were randomly assigned, received intended treatment, and were analysed for the primary outcome	Figure 1 Appendices 2,3,8
	13b	For each group, losses and exclusions after randomisation, together with reasons	Figures 1 Appendices 2,3,8
Recruitment	14a	Dates defining the periods of recruitment and follow-up	Page 7
	14b	Why the trial ended or was stopped	Page 7
Baseline data	15	A table showing baseline demographic and clinical characteristics for each group	Table 1
Numbers analysed	16	For each group, number of participants (denominator) included in each analysis and whether the analysis was by original assigned groups	Table 1
Outcomes and estimation	17a	For each primary and secondary outcome, results for each group, and the estimated effect size and its precision (such as 95% confidence interval)	Tables 1-4
	17b	For binary outcomes, presentation of both absolute and relative effect sizes is recommended	N/A
Ancillary analyses	18	Results of any other analyses performed, including subgroup analyses and adjusted analyses, distinguishing pre-specified from exploratory	N/A
Harms	19	All important harms or unintended effects in each group (for specific guidance see CONSORT for harms)	Appendix 7
**Discussion**			
Limitations	20	Trial limitations, addressing sources of potential bias, imprecision, and, if relevant, multiplicity of analyses	Page 24
Generalisability	21	Generalisability (external validity, applicability) of the trial findings	Pages 24
Interpretation	22	Interpretation consistent with results, balancing benefits and harms, and considering other relevant evidence	Pages 23-26
**Other information**			
Registration	23	Registration number and name of trial registry	Page 3
Protocol	24	Where the full trial protocol can be accessed, if available	Page 3
Funding	25	Sources of funding and other support (such as supply of drugs), role of funders	Page 26

For peer review only

For peer review only - http://bmjopen.bmj.com/site/about/guidelines.xhtml

*We strongly recommend reading this statement in conjunction with the CONSORT 2010 Explanation and Elaboration for important clarifications on all the items. If relevant, we also recommend reading CONSORT extensions for cluster randomised trials, non-inferiority and equivalence trials, non-pharmacological treatments, herbal interventions, and pragmatic trials. Additional extensions are forthcoming: for those and for up to date references relevant to this checklist, see www.consort-statement.org.

For peer review only
